# Gynecological malignancy signal triage: A two-dimensional framework integrating US-derived FAERS data with comparative US–China GBD trajectories

**DOI:** 10.1371/journal.pone.0361817

**Published:** 2026-10-29

**Authors:** Ying Ma, Dandan Li, Jieru Duan, Zhuowen Sun, Qirong Dou, Tao Wu, Na Gao

**Affiliations:** 1 Department of Obstetrics and Gynecology, The First Affiliated Hospital of Dalian Medical University, Dalian, Liaoning, China; 2 Department of Medical Oncology, The Second Affiliated Hospital of Dalian Medical University, Dalian, Liaoning, China; University of Science and Technology of Fujairah, YEMEN

## Abstract

**Background:**

Cancer-related adverse-event reports are difficult to interpret because statistical disproportionality, stimulated reporting, and background disease burden are distinct phenomena. To address this, we formalized a two-dimensional prioritization framework that combines FAERS signal credibility with independent GBD burden context. Because the FAERS dataset is overwhelmingly of US origin, we utilized China exclusively as a prespecified macro-epidemiological GBD comparator, strictly avoiding cross-national pharmacovigilance extrapolations.

**Methods:**

We analyzed FAERS reports (2004–2023) and female GBD 2023 estimates for China and the United States. Four disproportionality algorithms (ROR, PRR, IC, and EBGM) were evaluated for 150 high-frequency primary-suspect drug–event pairs (50 per cancer group). We report any-algorithm positivity and four-algorithm concordance alongside case-definition sensitivity analyses. GBD trends, age–period–cohort models, and exploratory projections to 2053 were integrated to provide population-level context, explicitly not as exposure denominators or for causal validation.

**Results:**

FAERS cohorts yielded 19,717 cancer-associated report records. Among 150 high-frequency candidate pairs, 42 met all four algorithmic thresholds. The top-50 candidate screen captured approximately 72.8%–75.8% of primary-suspect records, serving as a variance-reduction step rather than a complete all-drug screen. Drugs like ranitidine and talc demonstrated marked disproportionality but also exhibited reporter and temporal features heavily compatible with stimulated reporting. Time-to-onset distributions reflected recorded administrative reporting intervals rather than biological carcinogenesis. GBD estimates revealed divergent China–US trajectories, notably a rising baseline for US uterine cancer.

**Conclusions:**

The formalized F × G framework operates as a transparent triage tool, not a causal classifier. FAERS disproportionality generates hypotheses; GBD trajectories indicate potential public-health priority. All prioritized signals—particularly those involving consumer products or immunosuppressants—require formal causal evaluation in longitudinal exposure-denominator datasets.

## 1. Introduction

Although grouped under the broad umbrella of gynaecological malignancies, cervical, ovarian, and uterine cancers represent fundamentally distinct clinical and biological entities, characterized by divergent etiologies, molecular pathogenesis, and clinical trajectories [1,2]. While global estimates demonstrate that these three malignancies collectively contribute to substantial morbidity and mortality, their epidemiological burden exhibits marked geographic and temporal heterogeneity [3]. These dynamic population profiles are continuously shaped by demographic shifts, particularly population aging and evolving reproductive trends [4,5]. For instance, despite being largely preventable through human papillomavirus vaccination and structured screening programs, cervical cancer continues to cause disproportionate disease burden in regions with constrained healthcare infrastructure [6,7]. Ovarian cancer, conversely, maintains a persistently dismal mortality-to-incidence ratio—a clinical reality exacerbated by the inability of large-scale screening trials to confer a significant survival benefit [2,3,8]. Meanwhile, uterine cancer incidence has surged continuously across the United States, spanning multiple age, period, and birth-cohort strata [9,10]. These contrasting population baselines underscore that gynaecological malignancies cannot be evaluated as a monolithic endpoint when screening for potential treatment-related safety signals. Interpreting any reported drug-cancer association requires explicit alignment with the specific disease biology, the reporting behavior within the database, and the background epidemiological trajectory of the exposed population.

Superimposed on this clinical heterogeneity, evaluating suspected drug-induced malignancies within spontaneous reporting systems (SRSs) presents formidable analytical challenges. An adverse event report capturing a malignancy following drug exposure may represent a true biological hazard; alternatively, it may reflect severe confounding by indication, underlying disease progression, heightened diagnostic vigilance, or stimulated reporting driven by media coverage and mass litigation. Although large databases such as the FDA Adverse Event Reporting System (FAERS) offer high statistical power to detect unusual reporting clusters, standard quantitative signal detection algorithms (e.g., ROR, PRR, EBGM, and IC) merely measure relative disproportionality within the database. They mathematically cannot represent clinical incidence or relative risk, and are inherently vulnerable to distortion from masking effects and notoriety bias [11–14]. Therefore, utilizing spontaneous reporting databases for safety hypothesis generation is valuable only when these structural constraints are systematically integrated into signal interpretation [15].

To operationalize effective safety-signal triage, it is imperative to separate statistical disproportionality from public-health priority. To this end, we constructed a two-dimensional prioritization matrix (F × G) that cross-links micro-level drug safety signals (the FAERS dimension) with macro-level epidemiological trajectories (the GBD dimension). Recognizing the inherent limitations of spontaneous reporting, our framework avoids generating a flawed “causal score” and instead functions strictly as a structured triage matrix. It evaluates algorithmic concordance and explicitly penalizes known litigation artifacts on the FAERS axis, while independently assessing the context of the disease burden on the GBD axis. Ultimately, this framework does not alter or prove the causal credibility of a signal; rather, it dictates validation priority. It ensures that robust signals emerging within worsening epidemiological contexts (such as the surging uterine cancer baseline in the US) are prioritized for immediate pharmacoepidemiological attention.

## 2. Methods

### 2.1. Study design and data sources

This retrospective, observational pharmacovigilance study used the provided processed FAERS quarterly exports spanning the first quarter of 2004 through the fourth quarter of 2023. The exports link demographic (DEMO), drug (DRUG), reaction (REAC), outcome (OUTC), report-source (RPSR), indication (INDI), and therapy (THER) modules using PRIMARYID and CASEID. Female GBD 2023 estimates for China and the United States were downloaded from the IHME/GBD Results Tool on 15 June 2026. The FAERS component is therefore interpreted as an analysis of the provided reporting extracts, while GBD supplies independent population-level context. The FAERS case-selection and analytical workflow is summarized in Fig 1.

**Fig 1:**
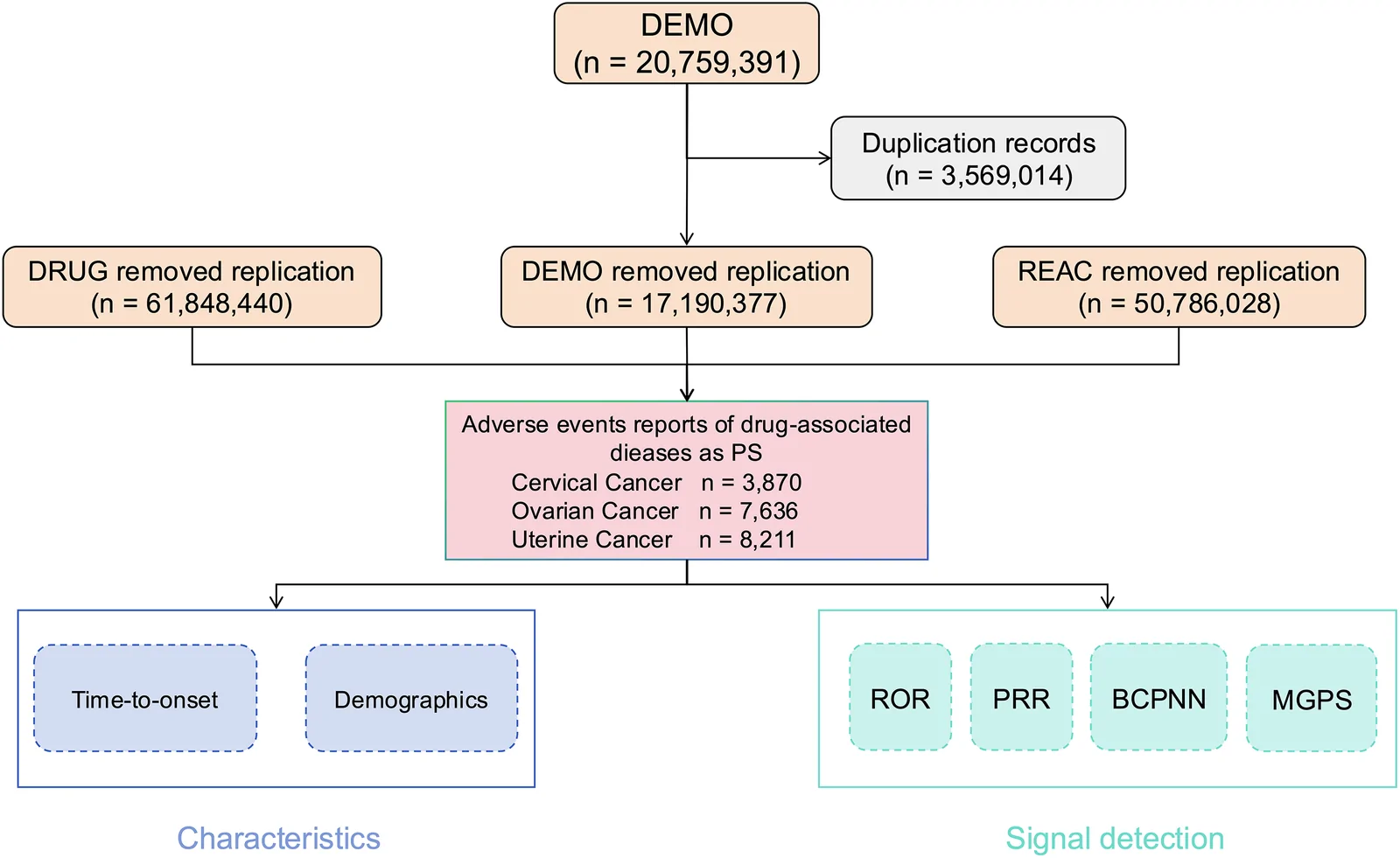
FAERS case selection and analytical workflow, 2004-2023. Quarterly demographic (DEMO), drug (DRUG) and reaction (REAC) files were deduplicated and linked before characterisation, time-to-onset analysis and four-algorithm disproportionality screening. The flow diagram reports the source-export totals for cervical, ovarian and uterine cancer. PS primary suspect, ROR reporting odds ratio, PRR proportional reporting ratio, BCPNN Bayesian confidence propagation neural network, MGPS multi-item gamma Poisson shrinker.

### 2.2. Case definition and deduplication

Cervical, ovarian, and uterine-cancer records were identified from explicit MedDRA PT vectors rather than treating all gynecological neoplasms as a single homogenous endpoint. Specifically, the cervical vector included CERVIX CARCINOMA, stage 0–IV terms, and CERVIX CARCINOMA RECURRENT. The ovarian vector included OVARIAN CANCER, stage I–IV, RECURRENT, METASTATIC, and specified advanced/biomarker-positive terms. The uterine vector included UTERINE CANCER, METASTATIC UTERINE CANCER, and ENDOMETRIAL CANCER, including stage 0–IV, RECURRENT, METASTATIC, and specified biomarker-positive terms. Therefore, “uterine cancer” serves as the overarching anatomical and analytical category throughout this study, while “endometrial cancer” is strictly mapped as a specific histological subset within the FAERS case definition. These terms are no longer treated as synonymous or mathematically interchangeable. The case-selection flow and cross-category PRIMARYID overlap are summarized in S1 and S2 Tables, and the MedDRA PT definitions, including recurrence, metastatic, and stage-specific terms, are listed in S3 Table.

To ensure strict reproducibility of the signal detection analysis, the data processing and deduplication workflow was executed in the following explicit sequence: (1) raw quarterly module import; (2) exact duplicate removal across all tables; (3) latest-case follow-up selection sorted by CASEID, FDA_DT, and PRIMARYID; (4) module linkage; (5) PT-based cancer classification; and (6) removal of male records or those with missing/implausible demographic data; and (7) role-based primary-suspect drug selection. Regarding Step 6, because FAERS is a spontaneous system susceptible to coding omissions, retaining records with “unknown” or “missing” sex for female-specific malignancies risks exposure misclassification. We adopted a strict complete-case approach, categorically excluding any record lacking explicit female demographic coding to ensure biological plausibility. The archived global flow file separately reports module-level deduplication totals prior to cancer-specific extraction.

It is necessary to distinguish between the analytical units used in different phases of this study. The processed cancer-specific DEMO exports contain one rigorously deduplicated row per patient (unique PRIMARYID), which were utilized to characterize cohort-level demographic patterns. In contrast, the contingency tables supplied for disproportionality screening were purposefully constructed using drug–event pair units. These pairs were generated immediately following the primary-suspect restriction. Regarding cross-category contribution, a single deduplicated patient report containing multiple distinct malignancy PTs can act as a case in more than one cancer-specific contingency table. This strategy ensures that the multi-algorithm matrix captures the specific reporting background without artificial truncation. Consequently, these signal tables should not be interpreted as independent cancer incidence counts, but rather as matrices for hypothesis prioritization.

### 2.3. Drug normalization and report characterization

Drug names were normalized with MedEx UIMA version 1.3.8, utilizing the generic, brand, alias, and combination-product mappings available in the project files. The resulting ingredient strings were mapped to ATC codes using the ATC search platform; the ATC level used for macro-level visualization was the first ATC character (anatomical main group). Reporter type and peak-year concentration for selected product groups are summarized in S4 Table. Recent pharmacovigilance work also recommends linking spontaneous-reporting signals to longitudinal healthcare data during signal management [16–18].

Primary-suspect (PS) status was strictly required for drug-level screening. Combination products were retained as their mapped ingredient strings when the mapping was available, whereas unresolved product-family strings were not treated as equivalent to a single prescription ingredient. These mapping rules and any remaining product-family ambiguity are explicitly reported so that the scope of the signal estimates remains transparent. Importantly, while restricting the analysis to primary-suspect records isolates the reporting behavior most directly attributed to the drug by the submitter, it does not emulate a controlled epidemiological design. This restriction does not eliminate confounding by indication, the effects of concomitant treatments, reverse causality, or the broader selection biases inherent to spontaneous pharmacovigilance databases.

### 2.4. Disproportionality algorithms and the two-dimensional prioritization matrix (F × G)

To mitigate the statistical noise and inflated false-discovery rates characteristic of the low-frequency “long tail” in FAERS, our primary disproportionality analysis was restricted to a prespecified high-frequency candidate screen comprising the top 50 most frequently reported primary-suspect drugs for each malignancy. This threshold functions as a variance-reduction strategy, ensuring that subsequent multi-algorithm analyses operate on robust, adequately powered contingency tables while inherently limiting the multiple-testing burden to a predefined 150 candidate pairs (50 drugs × 3 cancers).

For each drug–event pair, a two-by-two contingency table was constructed. The cell definitions are presented in S5 Table, and the formulas and prespecified positivity thresholds for ROR, PRR, IC, and EBGM are summarized in S6 Table. Four disproportionality algorithms were employed: the Reporting Odds Ratio (ROR), Proportional Reporting Ratio (PRR), Empirical Bayes Geometric Mean (EBGM), and Information Component (IC). To address zero-cell handling in frequentist calculations (ROR and PRR), a Haldane-Anscombe half-step correction (adding 0.5 to all contingency cells) was applied. For the Bayesian algorithms (IC and EBGM), inherent shrinkage parameters toward the prior distribution mean mathematically penalize sparse data, naturally mitigating the false-discovery rate without requiring overly conservative Bonferroni adjustments.

The positivity thresholds were defined as follows: ROR lower 95% CI ≥ 1 with a ≥ 3; PRR ≥ 2, χ2 ≥ 4, with a ≥ 3; EBGM05 ≥ 2; and IC025 ≥ 0. Any-algorithm positivity was retained solely as an exploratory sensitivity screen and was not equated with a robust signal, whereas four-algorithm concordance was utilized as the principal signal set.

To systematically triage these signals, we explicitly defined a Two-Dimensional Prioritization Matrix (F × G) to determine the final validation priority.

The FAERS Dimension (F-score): This axis evaluates report-level evidence and algorithmic concordance. It is graded from F0 to F3: F0: No prespecified signal met; F1: Weak evidence (met only one algorithm or sparse evidence with <3 reports); F2: Moderate evidence (positivity in at least two algorithms with ≥3 reports); F3: Strong concordance (positivity across all four algorithms with an adequate case count).

Artifact Penalty: To mitigate stimulated reporting, a pre-specified artifact flag was introduced. If a signal exhibited a single-year concentration spike, a disproportionately high attorney-coded reporter ratio, or consumer-product ambiguity, its FAERS grade was downgraded by one level (e.g., F3 becomes F2).

The GBD Dimension (G-score): This independent axis evaluates the macro-level population burden trajectory using GBD estimates. It is graded from G0 to G2: G0: Stable or declining age-standardized incidence rate (ASR); G1: Uncertain, mixed, or fluctuating ASR trajectory; G2: Statistically significant increasing ASR trajectory.

The Decision Rule (Matrix Output): The final validation priority is assigned by cross-tabulating the F and G scores. The highest validation priority is assigned to F3–G2 combinations (strong FAERS concordance in a worsening epidemiological context). High priority is assigned to F2–G2, and moderate priority to F2–G0/G1. Low or uncertain priority is assigned to F0–F1 pairs. Signals triggering the artifact penalty are diverted to an “Artifact-focused review” tier, avoiding unnecessary clinical alarm. The resulting classifications for ranitidine, talc, tamoxifen, adalimumab, and other selected drug-cancer pairs are shown in S7 Table.

Importantly, this matrix assigns priority for subsequent rigorous pharmacoepidemiological validation; it mathematically does not convert GBD population burden into an exposure denominator for FAERS, nor does it establish definitive causal inference.

### 2.5. Recorded time to onset

Time-to-onset (TTO) was calculated from the recorded treatment start date (START_DT in the THER module) to the recorded adverse-event onset date in the processed DEMO files. Records with missing or non-numeric dates, non-positive intervals, or extreme intervals exceeding 3,600 days were excluded; strictly one valid interval per deduplicated PRIMARYID was retained. Reflecting the high degree of temporal missingness inherent to spontaneous reporting, the valid fractions of records meeting these criteria were 15.3% for cervical cancer, 14.8% for ovarian cancer, and 13.1% for uterine cancer.

Given the lack of reliable exposure end-dates in FAERS, no exposure-duration reconstruction or right-censoring mechanisms were utilized. The applied Weibull models serve as uncensored, purely descriptive fits to right-skewed observational data, rather than comparative survival analyses. Consequently, we explicitly define these metrics as administrative treatment-to-event reporting intervals. The medians, > 360-day proportions, and distributional shapes characterize reporting patterns and diagnostic timelines; they do not demonstrate pharmacological latency, carcinogenic induction, or a true clinical hazard function. The Weibull shape parameter (β) is reported strictly as a mathematical descriptor of the data distribution.

### 2.6. GBD trend, age-period-cohort, decomposition and forecast analyses

Female GBD 2023 estimates were queried for China and the United States (GBD cause hierarchies: B.4.3 Cervical cancer, B.4.4 Ovarian cancer, B.4.5 Uterine cancer) for four measures (Deaths, DALYs, Prevalence, and Incidence). Queries extracted both absolute Numbers and Rates per 100,000 for 1990–2023. Age-standardized rates (ASR) were computed using the WHO World Standard Population, with uncertainty intervals reflecting the GBD database-provided 95% UIs. The GBD 2023 source and disease-burden context are documented in the published GBD analysis [19]. To ensure robust comparability and code verification across structural disparities, our data extraction and processing strictly adhered to reproducible tool-based workflows [20].

Joinpoint input files were generated in rates per 100,000. The archived output utilized 0–5 candidate joinpoints, evaluating structural segments via the Grid Search method and a permutation test (4,500 permutations) applying familywise error rate control. A recent population-level Joinpoint analysis provides a methodological precedent for this segmented-trend approach [21]. The China-specific burden estimates are also considered alongside published GBD analyses [22].

To resolve the non-identifiability problem inherent to Age-Period-Cohort (APC) models, we constrained the reference parameters to the median age group, period, and birth cohort. Analyses utilized five-year groupings. For the Bayesian age-period-cohort (BAPC) implementation, we integrated historical data (1990–2023) with UN 2024–2053 population projections. Model smoothing and overdispersion handling were achieved using a first-order random walk (RW1) prior for age, period, and cohort components (gf = 5, secondDiff = TRUE). Recent age-period-cohort applications provide a comparable public-health trend-analysis context [23].

Changes in absolute disease burden were quantified using Das Gupta’s decomposition method, which we interpret strictly as the arithmetic attribution of population growth, ageing, and epidemiological rate changes, not as causal mediation or propagated probabilistic uncertainty. For long-term BAPC projections, models fitted to 1990–2013 data were compared with observed 2014–2023 rates to assess directional performance rather than provide independent validation. Forecasts for 2024–2053 remain purely exploratory scenarios characterized by visibly widening 95% UIs; they are not deterministic predictions. Related burden-projection work likewise treats long-horizon estimates as modelled scenarios rather than observed future outcomes [24].

Descriptive statistics summarized the FAERS exports. Continuous TTO variables are reported as medians and IQRs; categorical variables are reported as counts and percentages. Signal estimates are reported with 95% CIs where retained in the archived output. In addition to the prespecified algorithm thresholds, we calculated BH-FDR and 150-comparison Bonferroni sensitivity counts. The revised text distinguishes report frequency, disproportionality, signal prioritization, and causal validation. Statistical significance is not interpreted as evidence of causality.

All data wrangling, statistical modeling, and graphical visualizations were executed using R software, version 4.4.3 (R Foundation for Statistical Computing, Vienna, Austria), operated through the RStudio integrated development environment, version 2024.12.1 + 563 (Posit Software, PBC, Boston, MA, USA) on a macOS platform. Supplementary data extraction and structural checks were conducted using Python. The methodological workflow and manuscript preparation strictly adhered to the Reporting and Evaluation Guidelines for Pharmacovigilance Studies in Spontaneous Reporting Systems (READUS-PV) statement [11].

From an ethical perspective, both the FAERS database and the GBD 2023 dataset comprise exclusively publicly accessible, fully de-identified, and aggregated secondary data, which were accessed for research purposes on 15 Jun 2026. Because no individually identifiable private health information was accessed or processed by the authors during or after data collection, this study did not meet the criteria for human subject research. Consequently, the requirement for institutional review board (IRB) approval and documented informed consent was formally waived in accordance with standard ethical guidelines for secondary database analyses.

## 3. Results

### 3.1. Descriptive characteristics of adverse event reports

Following the cancer-specific extraction and deduplication workflow, the FAERS exports contained 19,717 deduplicated patient records: 3,870 CC, 7,636 OC, and 8,211 UC (Table 1). Because these cancer categories are not mutually exclusive at the PRIMARYID level (a patient may have multiple concurrent primary malignancies), their sum represents a category-specific record total rather than an absolute count of mutually exclusive individuals. The United States contributed the majority of reports, while China contributed only 11–27 reports per cancer category (≤0.3%).

**Table 1:** Baseline characteristics of drug-associated cervical, ovarian, and uterine cancer adverse event reports in the FAERS database (2004–2023).

Characteristics	CC cases, n (%)	OC cases, n (%)	UC cases, n (%)
Total	3,870 (100.0)	7,636 (100.0)	8,211 (100.0)
Weight, kg			
< 50	89 (2.3)	174 (2.3)	99 (1.2)
50–100	488 (12.6)	1,118 (14.6)	944 (11.5)
> 100	54 (1.4)	86 (1.1)	259 (3.2)
Unknown	3,239 (83.7)	6,258 (82.0)	6,909 (84.1)
Age, years			
< 18	17 (0.4)	30 (0.4)	11 (0.1)
18–64	2,050 (53.0)	3,006 (39.4)	3,498 (42.6)
65–85	247 (6.4)	1,220 (16.0)	1,430 (17.4)
> 85	19 (0.5)	58 (0.8)	59 (0.7)
Unknown	1,537 (39.7)	3,322 (43.5)	3,213 (39.1)
Reporter country^a^			
United States (US)	2,156 (55.7)	4,877 (63.9)	5,309 (64.7)
Japan (JP)	201 (5.2)	391 (5.1)	329 (4.0)
Canada (CA)	152 (3.9)	328 (4.3)	308 (3.7)
Germany (DE)	186 (4.8)	155 (2.0)	164 (2.0)
United Kingdom (GB)	139 (3.6)	212 (2.7)	153 (1.9)
China (CN)	11 (0.3)	27 (0.3)	22 (0.2)
Other	1,025 (26.5)	1,646 (21.7)	1,926 (23.5)
Reporter qualification			
Consumer	1,366 (35.3)	2,842 (37.2)	3,098 (37.7)
Physician	1,195 (30.9)	2,168 (28.4)	2,530 (30.8)
Health professional	145 (3.7)	274 (3.6)	234 (2.8)
Pharmacist	60 (1.6)	124 (1.6)	122 (1.5)
Unknown	1,104 (28.5)	2,228 (29.2)	2,227 (27.1)
Clinical outcome^b^			
OT	2,603 (67.3)	4,149 (54.3)	5,469 (66.6)
Death	286 (7.4)	1,000 (13.1)	538 (6.6)
HO	389 (10.1)	739 (9.7)	859 (10.5)
LT	64 (1.7)	143 (1.9)	118 (1.4)
DS	17 (0.4)	26 (0.3)	34 (0.4)
RI	1 (<0.1)	0 (0.0)	0 (0.0)
Unknown	510 (13.2)	1,579 (20.7)	1,193 (14.5)

Notes: CC: Cervical cancer; OC: Ovarian cancer; UC: Uterine cancer; FAERS: FDA Adverse Event Reporting System.

^a^Top five countries plus China are listed.

^b^OT: Other medically important conditions; Death: Death outcome (spelled out to distinguish from Germany’s ISO code DE); HO: Hospitalization (initial or prolonged); LT: Life-threatening; DS: Disability; RI: Required intervention to prevent permanent impairment/damage.

### 3.2. Drug classifications, temporal trends, and reporting patterns

Primary-suspect drug reporting was concentrated in alimentary-tract/metabolism and antineoplastic/immunomodulating products (Fig 2A). Annual reporting increased gradually through 2020 and showed a sharp 2021 peak, with ranitidine accounting for most peak-year records (Fig 2B). The United States was the leading reporting country in all three cancer groups (Fig 2C). Attorney-coded reports constituted a substantial fraction of ranitidine records during this period, consistent with litigation-related stimulated reporting (S4 Table). FAERS does not formally encode litigation status, however, and attorney coding is not equivalent to confirmed active litigation. Reporter type therefore serves as an indirect indicator, and media attention or notoriety effects may also have contributed to the reporting surge.

**Fig 2:**
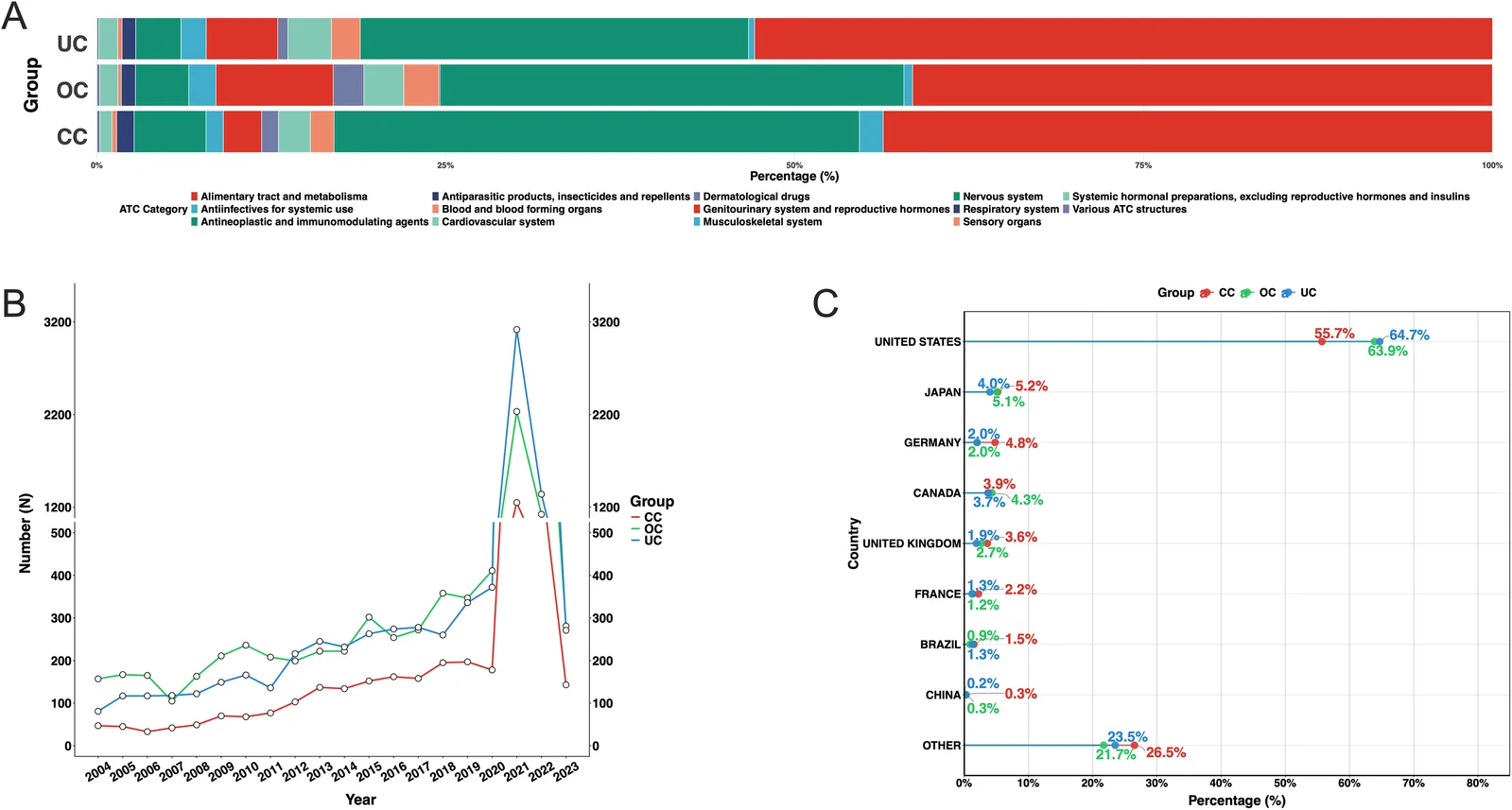
Reporting patterns and temporal trends of gynaecological cancer adverse events in the FAERS database. (A) Proportion of primary-suspect drugs categorized by Anatomical Therapeutic Chemical (ATC) classes across the cervical (CC), ovarian (OC), and uterine cancer (UC) cohorts. (B) Longitudinal trends of annual report counts from 2004 to 2023, with the 2021 peak shown explicitly. (C) Geographic distribution of the primary reporting countries. These curves describe spontaneous-reporting activity and should not be interpreted as population incidence or baseline medicine exposure.

### 3.3. Frequently reported specific drugs and reporting profiles

The top-50 lists for cervical, ovarian, and uterine cancer (Fig 3A-C, respectively) were used as a prespecified high-frequency candidate screen because the study objective was to identify interpretable, multi-algorithm signals rather than estimate exhaustive exposure risks for every recorded product. The top 50 drugs retained 72.8%–75.8% of all primary-suspect report-drug units. An additional 108–230 drugs with at least three primary-suspect reports remained outside the top 50, but these formed a fragmented, low-frequency tail susceptible to unstable estimates. Expanding the screen to 100 drugs increased coverage to 80.6%–83.3% (S8 Table). These estimates define a focused candidate screen and are not comparative exposure measures across all recorded products.

**Fig 3:**
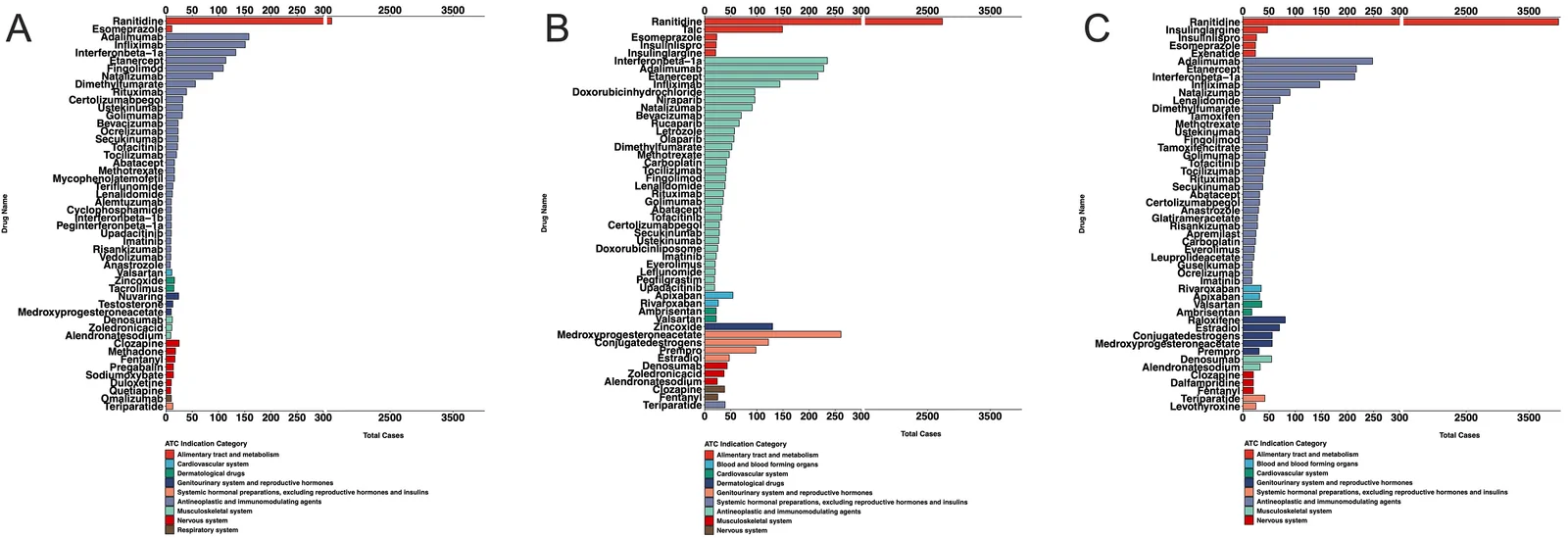
Top 50 most frequently reported primary-suspect drugs across gynaecological malignancies. Bar charts illustrate the distribution of the 50 most common primary-suspect drugs for (A) cervical cancer, (B) ovarian cancer, and (C) uterine cancer. Drugs are color-coded according to their Anatomical Therapeutic Chemical (ATC) indication categories. The x-axis uses a scale break because ranitidine contributes a large concentration of reports. Absolute case counts describe reporting frequency and do not imply causal epidemiological effect size.

### 3.4. Disproportionality analysis and signal generation

The top-50 screen generated 63 exploratory positive drug-event pairs that met at least one algorithmic threshold; 42 met all four thresholds and are displayed in Fig 4. Four-algorithm concordance was observed for 14 cervical, 16 ovarian, and 12 uterine cancer pairs, while BH-FDR and Bonferroni retained 27–32 and 21–26 pairs, respectively (S9 Table). Ranitidine, talc, zinc oxide, and tamoxifen produced extreme reporting disproportionality, while adalimumab and other immunosuppressants remain hypotheses for pharmacoepidemiologic validation. These findings prioritize candidates for follow-up but do not establish a novel adverse drug reaction or a causal effect.

**Fig 4:**
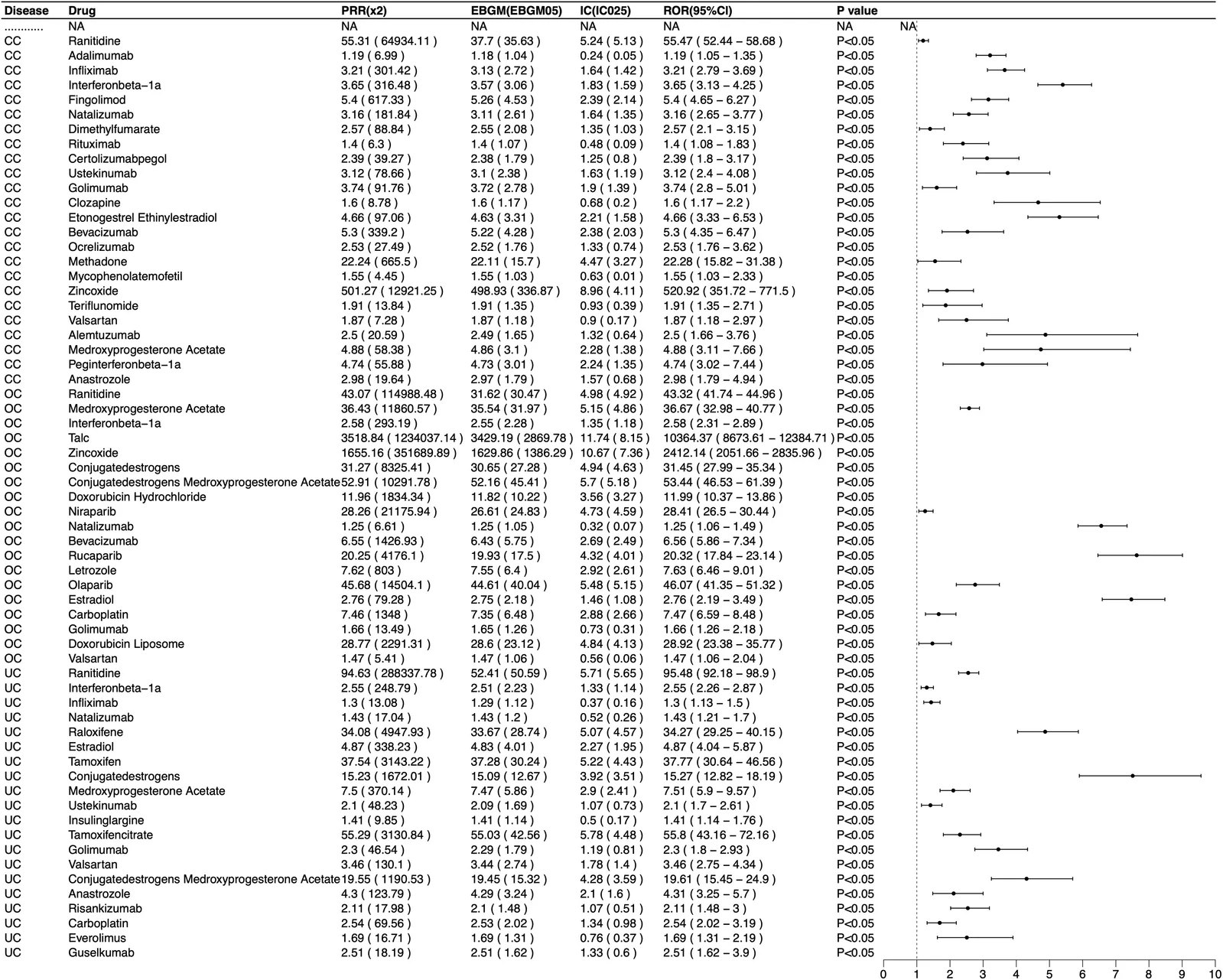
Forest plot of positive disproportionality signals across gynaecological malignancies. The plot illustrates the 42 drug–event pairs that met the positivity thresholds across all four algorithms (PRR, EBGM, IC, and ROR). Data are stratified by cervical cancer (CC), ovarian cancer (OC), and uterine cancer (UC). Reporting Odds Ratios (ROR) and 95% Confidence Intervals (CI) are visualized on a logarithmic scale to accommodate the wide range of estimates. Signal values indicate reporting disproportionality within FAERS and do not equate to clinical incidence rates or causal relative risks.

### 3.5. Time-to-onset distributions and parametric survival modeling

The valid TTO subsets contained 591 cervical, 1,133 ovarian, and 1,072 uterine cancer records after stringent date and interval filtering (Fig 5A-C; Table 2). Intervals exceeding 360 days accounted for 55.16%, 58.25%, and 63.71% of these cohorts, respectively (S10 Table).

**Fig 5:**
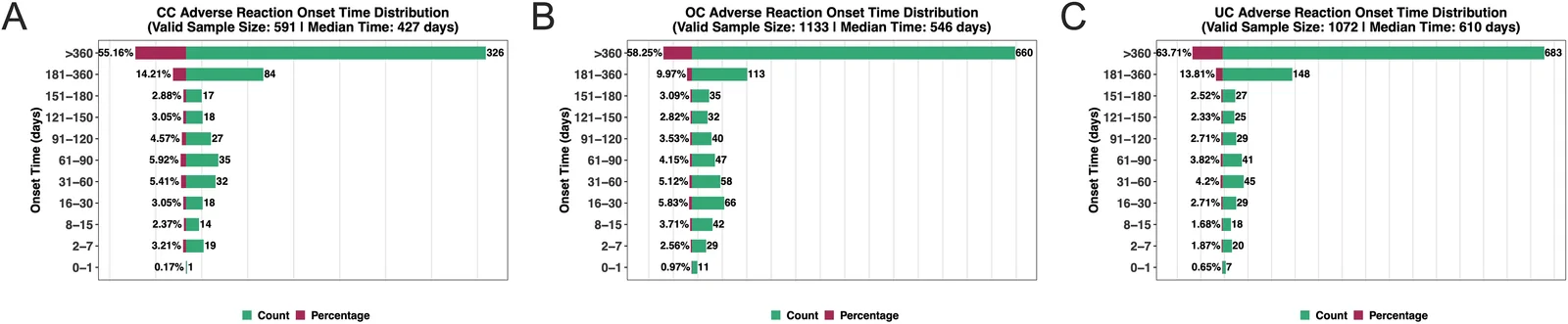
Time-to-onset distributions for drug-associated gynaecological cancer reports. Bar charts display reported treatment-to-event intervals for (A) cervical cancer (N = 591), (B) ovarian cancer (N = 1133), and (C) uterine cancer (N = 1072). These intervals are administrative timing measures calculated from recorded treatment start to recorded event onset. They characterize spontaneous reporting behaviors and structural database biases; they do not represent biological cancer latency or a clinical hazard function.

**Table 2:** Weibull parametric survival modeling of time-to-onset for gynaecological cancer adverse events.

Cohort	Valid reports (N)	Median (IQR), days	Range, days	Scale parameter αα (95% CI)	Shape parameter ββ (95% CI)
Cervical cancer (CC)	591	427 (122.5–1096.0)	1–3597	675.36 (609.40–748.45)	0.82 (0.77–0.88)
Ovarian cancer (OC)	1133	546 (111.0–1461.0)	1–3592	766.17 (705.89–831.60)	0.75 (0.71–0.78)
Uterine cancer (UC)	1072	610 (207.0–1453.5)	1–3577	867.86 (808.85–931.18)	0.89 (0.85–0.94)

Abbreviations: IQR, Interquartile Range; CI, Confidence Interval.

Note: The shape parameter (β) is strictly a mathematical descriptor of the observed interval distribution’s right-skewness. It does not represent a clinical hazard function, does not establish biological cancer latency, and should not be used to infer specific epidemiological reporting patterns, such as the Weber effect.

Rather than reflecting biological latency, these right-skewed distributions represent reported treatment-to-event intervals that are heavily shaped by structural factors in spontaneous reporting. As observed in the early incidence peaks, very short intervals frequently reflect left truncation, the unmasking of pre-existing undetected disease (protopathic bias), or immediate reporting following a delayed diagnosis. FAERS data lack the clinical granularity to distinguish these mechanisms from true acute events.

Subsequent Weibull modeling yielded shape parameters (β) below 1.0 across all cohorts (0.75–0.89). In the context of our analysis, a β < 1 mathematically describes an interval distribution with the highest density of reports occurring early relative to a long temporal tail. This is not an epidemiological hazard estimate. We interpret these fitted parameters strictly as descriptive indicators of reporting behaviors—such as diagnostic delay and subsequent retrospective reporting—completely separate from biological mechanisms of oncogenesis.

### 3.6. Spatiotemporal trends and Joinpoint analysis of gynaecological cancers (1990–2023)

Joinpoint analysis revealed divergent population-level trends across the three cancers (Fig 6A-C). The US uterine cancer category—which serves as the broader overarching GBD label, encompassing the substantial endometrial cancer subset identified in our FAERS analysis—showed increasing incidence (ASIR EAPC 1.67, 95% CI 1.57–1.77; Table 3). Given the stark asymmetry in FAERS reporting volumes (with China accounting for ≤0.3% of reports), the GBD data for China and the United States were compared strictly to illustrate divergent macro-epidemiological trajectories. This analysis contrasts population-level disease burdens; it does not constitute a cross-national pharmacovigilance comparison. The GBD results provide baseline context for potential public-health consequences, not evidence that any reported drug caused these population-level shifts.

**Fig 6:**
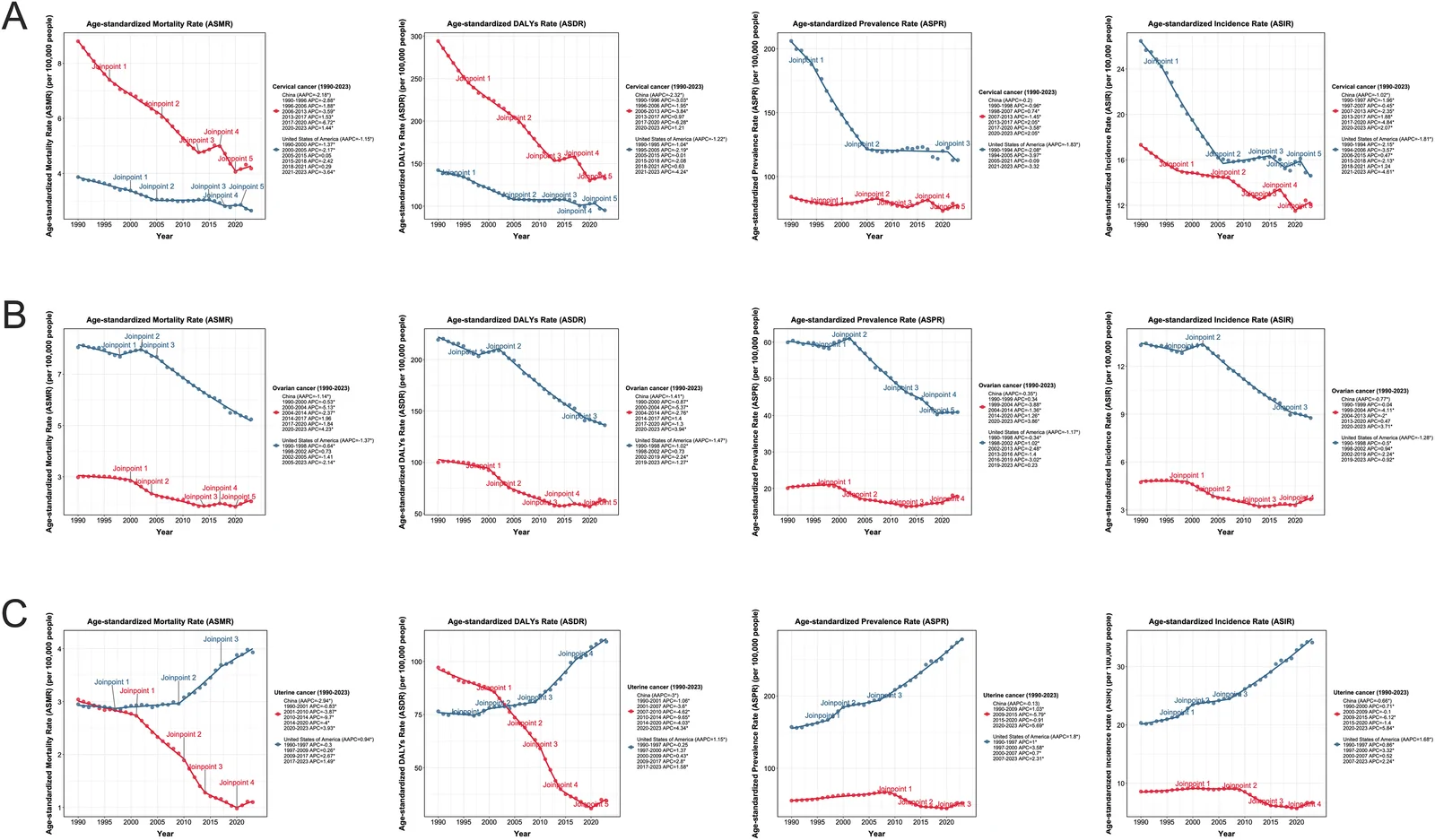
Joinpoint regression analysis of spatiotemporal trends in age-standardized epidemiological metrics for gynaecological cancers in China and the United States (1990–2023). Trajectories are stratified by cancer type: (A) Cervical cancer; (B) Ovarian cancer; and (C) Uterine cancer. Within each panel, the four subplots (from left to right) illustrate the Age-standardized Mortality Rate (ASMR), Age-standardized DALYs Rate (ASDR), Age-standardized Prevalence Rate (ASPR), and Age-standardized Incidence Rate (ASIR), all expressed per 100,000 population. Red markers and lines represent data for China, while blue markers and lines denote data for the United States. Individual dots indicate annual observational estimates derived from the Global Burden of Disease (GBD) 2023 framework. Solid lines depict the continuous modeled trend segments fitted by the Joinpoint software. Labeled “Joinpoints” (1, 2, 3…) mark the specific inflection years where statistically significant shifts in the temporal slope occurred. The embedded text boxes detail the Average Annual Percent Change (AAPC) for the entire observation period and the localized Annual Percent Change (APC) for each specific segment. An asterisk (*) denotes that the corresponding APC or AAPC is statistically significantly different from zero (P < 0.05).

**Table 3:** Absolute cases, age-standardized rates, and temporal trends of gynaecological cancer burden in China and the United States (1990–2023).

Cancer Type & Metric	Country	1990		2023		1990–2023
		Cases (95% UI)	ASR (95% UI)	Cases (95% UI)	ASR (95% UI)	EAPC (95% CI)
**Cervical cancer**						
ASMR	China	41,076 (30,145–59,131)	8.80 (6.45–12.75)	47,758 (29,981–62,579)	4.18 (2.66–5.46)	−2.27 (−2.39 to −2.16)
	USA	6,349 (5,548–7,311)	3.87 (3.42–4.43)	7,094 (6,102–8,009)	2.65 (2.28–2.99)	−0.99 (−1.10 to −0.89)
ASDR	China	1,457,115 (1,070,025–2,064,940)	294.14 (215.88–420.48)	1,430,054 (896,628–1,874,757)	132.72 (83.76–173.11)	−2.43 (−2.54 to −2.33)
	USA	215,089 (189,829–245,972)	142.37 (125.78–162.68)	218,681 (189,182–246,951)	95.20 (82.19–107.47)	−1.09 (−1.22 to −0.96)
ASPR	China	445,795 (301,749–642,609)	84.26 (57.89–122.19)	752,306 (481,273–1,011,544)	76.52 (48.77–103.69)	−0.17 (−0.26 to −0.07)
	USA	303,246 (237,021–381,385)	206.07 (161.30–259.10)	229,807 (180,217–287,321)	112.91 (88.31–141.31)	−1.75 (−2.06 to −1.44)
ASIR	China	86,808 (62,053–125,564)	17.32 (12.51–25.01)	124,686 (77,638–165,722)	12.06 (7.44–15.96)	−1.04 (−1.14 to −0.94)
	USA	39,632 (31,933–49,991)	26.46 (21.25–33.45)	31,260 (24,979–37,880)	14.60 (11.64–17.88)	−1.67 (−1.95 to −1.39)
**Ovarian cancer**						
ASMR	China	13,997 (10,410–18,305)	2.98 (2.22–3.90)	23,670 (19,076–28,752)	2.04 (1.67–2.48)	−1.79 (−2.03 to −1.54)
	USA	14,355 (12,931–15,680)	8.05 (7.32–8.76)	16,576 (14,716–18,420)	5.24 (4.69–5.81)	−1.39 (−1.56 to −1.22)
ASDR	China	501,308 (370,954–644,605)	99.83 (74.23–129.35)	686,054 (559,503–829,349)	62.35 (51.81–75.23)	−2.09 (−2.35 to −1.83)
	USA	356,040 (327,265–386,577)	219.24 (202.46–237.53)	381,320 (344,452–420,746)	136.14 (122.96–150.64)	−1.56 (−1.70 to −1.42)
ASPR	China	108,716 (77,311–138,937)	20.08 (14.51–26.01)	176,260 (144,556–215,050)	17.77 (14.46–21.77)	−0.98 (−1.24 to −0.73)
	USA	92,890 (80,275–106,657)	59.92 (51.58–69.02)	101,620 (85,957–117,588)	40.87 (34.77–47.55)	−1.43 (−1.61 to −1.26)
ASIR	China	24,091 (18,197–30,774)	4.73 (3.58–6.07)	38,969 (31,914–46,805)	3.66 (3.05–4.38)	−1.41 (−1.66 to −1.16)
	USA	22,103 (20,036–24,633)	13.32 (12.08–14.90)	24,496 (21,553–27,486)	8.76 (7.69–9.86)	−1.43 (−1.61 to −1.26)
**Uterine cancer**						
ASMR	China	13,940 (8,373–18,256)	3.04 (1.84–4.00)	12,941 (9,722–19,847)	1.10 (0.83–1.69)	−3.82 (−4.23 to −3.42)
	USA	5,672 (4,746–6,584)	2.95 (2.48–3.43)	12,951 (10,773–15,110)	3.93 (3.32–4.58)	1.01 (0.81 to 1.21)
ASDR	China	471,077 (284,518–612,099)	97.12 (58.59–125.92)	386,294 (290,505–590,688)	34.52 (26.00–52.72)	−3.90 (−4.30 to −3.50)
	USA	134,415 (113,766–156,811)	76.51 (64.57–89.44)	325,921 (275,088–384,885)	109.31 (93.11–129.23)	1.24 (1.07 to 1.42)
ASPR	China	269,517 (162,098–360,168)	55.32 (33.45–74.62)	580,165 (427,088–909,237)	51.92 (38.30–81.39)	−0.72 (−1.10 to −0.34)
	USA	262,100 (208,285–317,089)	157.11 (124.38–190.66)	790,039 (660,165–964,748)	278.43 (233.54–339.31)	1.80 (1.71 to 1.89)
ASIR	China	40,984 (24,818–53,697)	8.58 (5.18–11.28)	74,990 (53,630–119,885)	6.66 (4.75–10.71)	−1.37 (−1.73 to −1.00)
	USA	34,742 (27,745–41,176)	20.35 (16.20–24.23)	98,981 (83,315–122,581)	34.14 (28.81–42.25)	1.67 (1.57 to 1.77)

Abbreviations: USA, United States of America; ASR, Age-standardized rate (expressed per 100,000 population); ASMR, Age-standardized mortality rate; ASDR, Age-standardized DALYs (disability-adjusted life years) rate; ASPR, Age-standardized prevalence rate; ASIR, Age-standardized incidence rate; UI, Uncertainty interval (generated by the GBD modeling process); EAPC, Estimated annual percentage change; CI, Confidence interval.

Note: Absolute incident, prevalent, death, and DALYs cases were rounded to the nearest whole integer. Confidence intervals for EAPC that do not cross zero indicate a statistically significant trend (P < 0.05).

Joinpoint analysis showed distinct population-level trajectories. The United States generally showed smooth trends, including an increasing uterine cancer trajectory (ASDR APC approximately 2.8 in the highlighted segment), whereas China showed larger segment-to-segment changes, including declines in uterine cancer ASDR during 2010–2014 and increases in selected ovarian and uterine metrics after 2020. These differences are descriptive population patterns that may reflect demographic, diagnostic, and health-system factors; they are not attributed to a specific exposure in this study. The interpretation of these spatiotemporal segments is constrained to macro-level epidemiological shifts.

### 3.7. Age-period-cohort (APC) analysis of disease trajectories

The age-period-cohort analysis separated age, period, and cohort patterns for the three cancer groups. Cervical-cancer burden was concentrated in midlife in China, while several US incidence and prevalence measures peaked earlier and mortality remained higher at older ages. Period and cohort patterns generally moved in a favorable direction for cervical cancer in both settings (Fig 7). These patterns describe population heterogeneity and provide context for the design of future validation studies.

**Fig 7:**
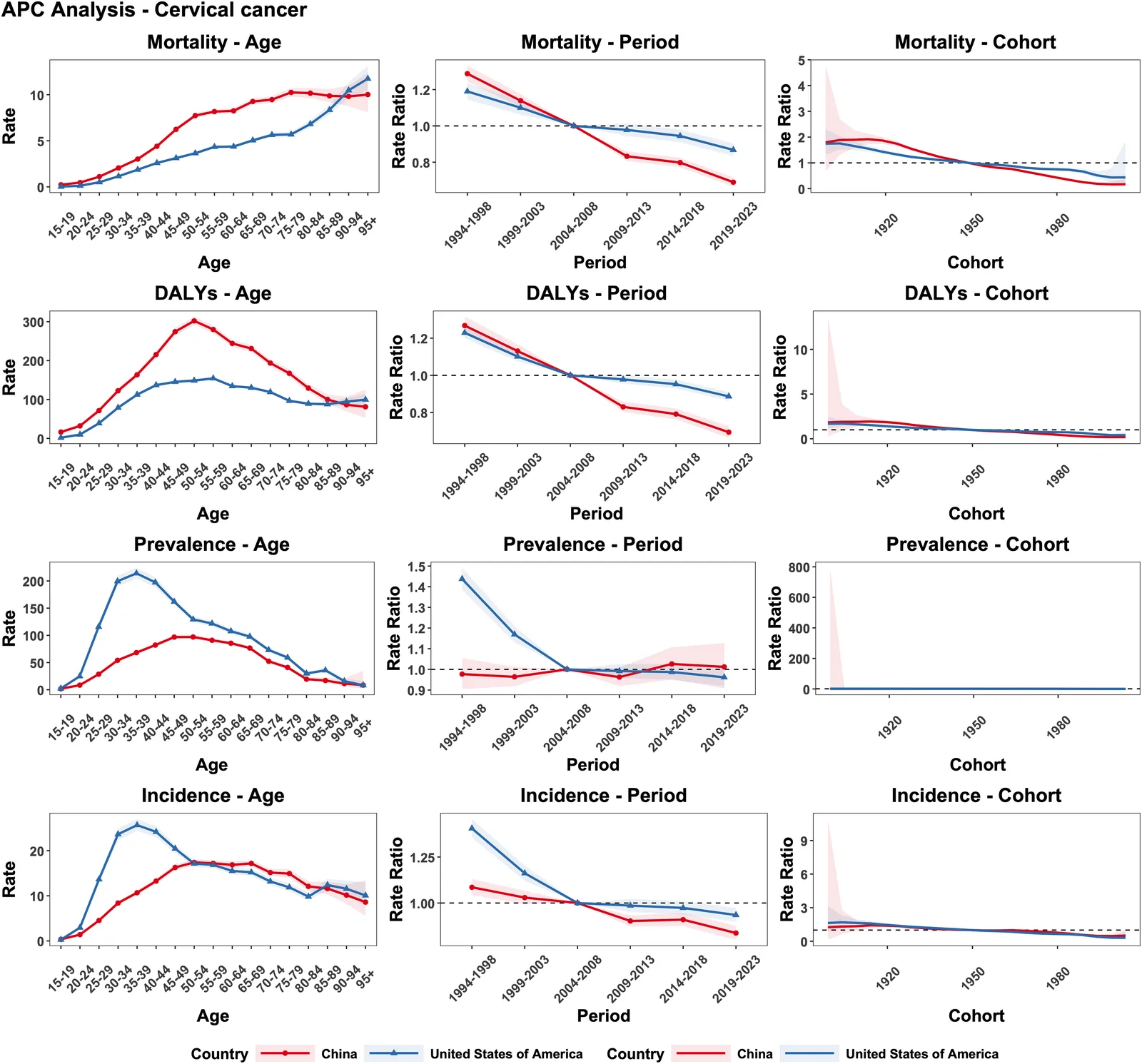
Age-period-cohort (APC) model estimates for cervical cancer burden in China and the United States. The panel is organized into a 4 × 3 grid. Rows (from top to bottom) represent four distinct epidemiological metrics: Mortality, Disability-Adjusted Life Years (DALYs), Prevalence, and Incidence. Columns (from left to right) depict the three temporal dimensions extracted from the APC framework: Age effects, Period effects, and Cohort effects. Red solid lines represent modeled estimates for China, while blue solid lines denote the United States. Shaded bands surrounding the estimation lines indicate the corresponding 95% confidence intervals (CIs). For the Age effect (left column), the y-axis displays the age-specific absolute rate (expressed per 100,000 population). For the Period (middle column) and Cohort (right column) effects, the y-axis displays the estimated Rate Ratio (RR). The black dashed horizontal line at RR = 1.0 serves as the reference risk level (representing the baseline reference period and reference birth cohort, respectively). Corresponding APC results for ovarian and uterine cancers are shown in S1 and S2 Figs, respectively.

Ovarian-cancer profiles were dominated by later-life age effects, with older age peaks in the United States and a more midlife distribution in China. Period effects declined in the United States, whereas the Chinese series showed a flatter or modestly increasing pattern in the most recent years (S1 Fig). The analysis is descriptive and does not identify the mechanisms underlying these differences.

Uterine-cancer patterns differed between the two countries. China showed midlife age peaks and declining period and cohort effects, whereas the United States showed increasing age-specific risk at older ages and stronger positive period effects after 2009 (S2 Fig). The cohort pattern is compatible with changing population risk across generations, but these aggregate data do not identify a single cause or permit attribution to obesity or another individual-level exposure. The extracted reference parameters serve strictly to baseline these descriptive models.

### 3.8. Age-specific demographic and epidemiological landscape in 2023

To describe the contemporary population distribution of disease burden, we evaluated age-specific estimates for the three gynaecological cancers in 2023 (Fig 8), separating absolute case numbers from rates per 100,000 population.

**Fig 8:**
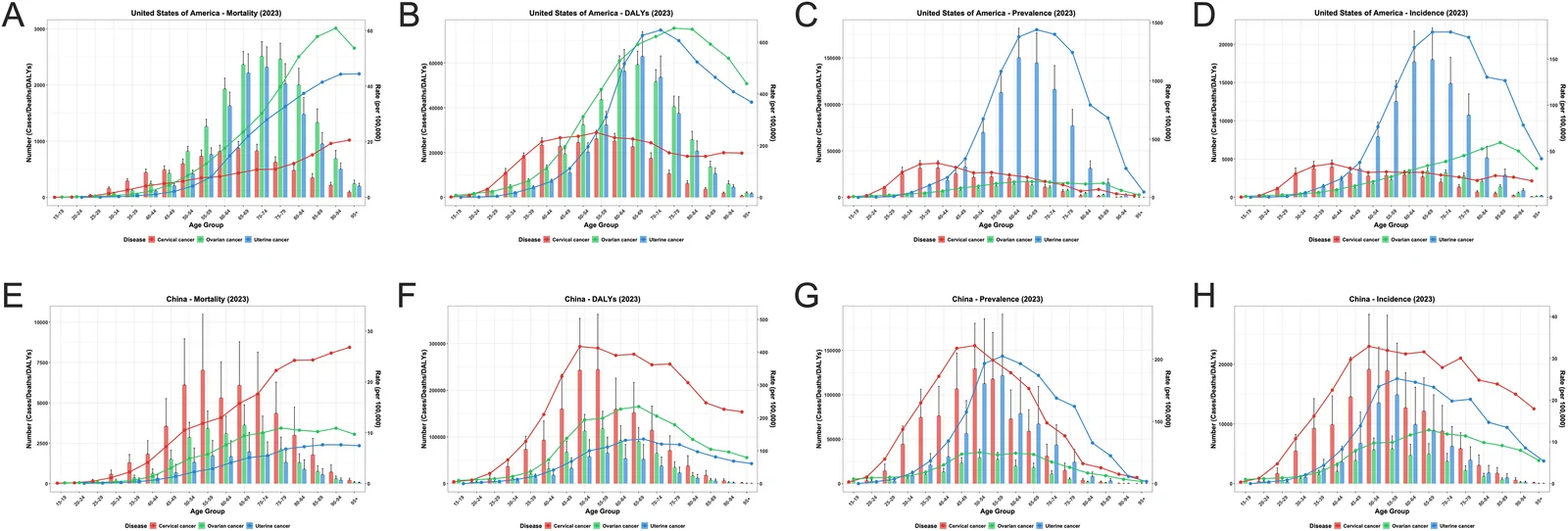
Age-specific demographic and epidemiological burden of gynaecological cancers in 2023. The panels display data for the United States (A–D) and China (E–H) across mortality, DALYs, prevalence, and incidence. Bar charts represent absolute population counts, while line graphs represent age-specific rates per 100,000 population. Error bars indicate 95% uncertainty intervals. Note: These data represent female GBD 2023 population estimates and do not attribute any cases to specific pharmacological exposures or FAERS reports.

In the United States (Fig 8A–D), absolute case numbers varied with the underlying population structure, whereas rates per 100,000 were highest at older ages for uterine and ovarian cancer. Cervical-cancer rates were concentrated earlier in adulthood, with mortality remaining elevated at older ages. These are population-level rate patterns rather than individual exposure risks.

In China (Fig 8E–H), cervical-cancer rates were concentrated in midlife, while ovarian and uterine rates were lower across most age groups and generally declined at older ages. The contrast with the United States is a population-level difference in age-specific burden and should not be interpreted as evidence for a particular drug or mechanism.

Together, the 2023 age-specific estimates provide ecological context for interpreting FAERS signals. They identify where a validated drug-associated signal could have greater population consequence, while preserving the distinction between background burden and medicine-attributable risk.

### 3.9. Decomposition of absolute burden changes (1990–2023)

We decomposed changes in absolute burden between 1990 and 2023 into population growth, population ageing, and epidemiological-rate change (Fig 9A-D). Population growth contributed positively in both countries, while the net direction depended on the balance between demographic components and rate change.

**Fig 9:**
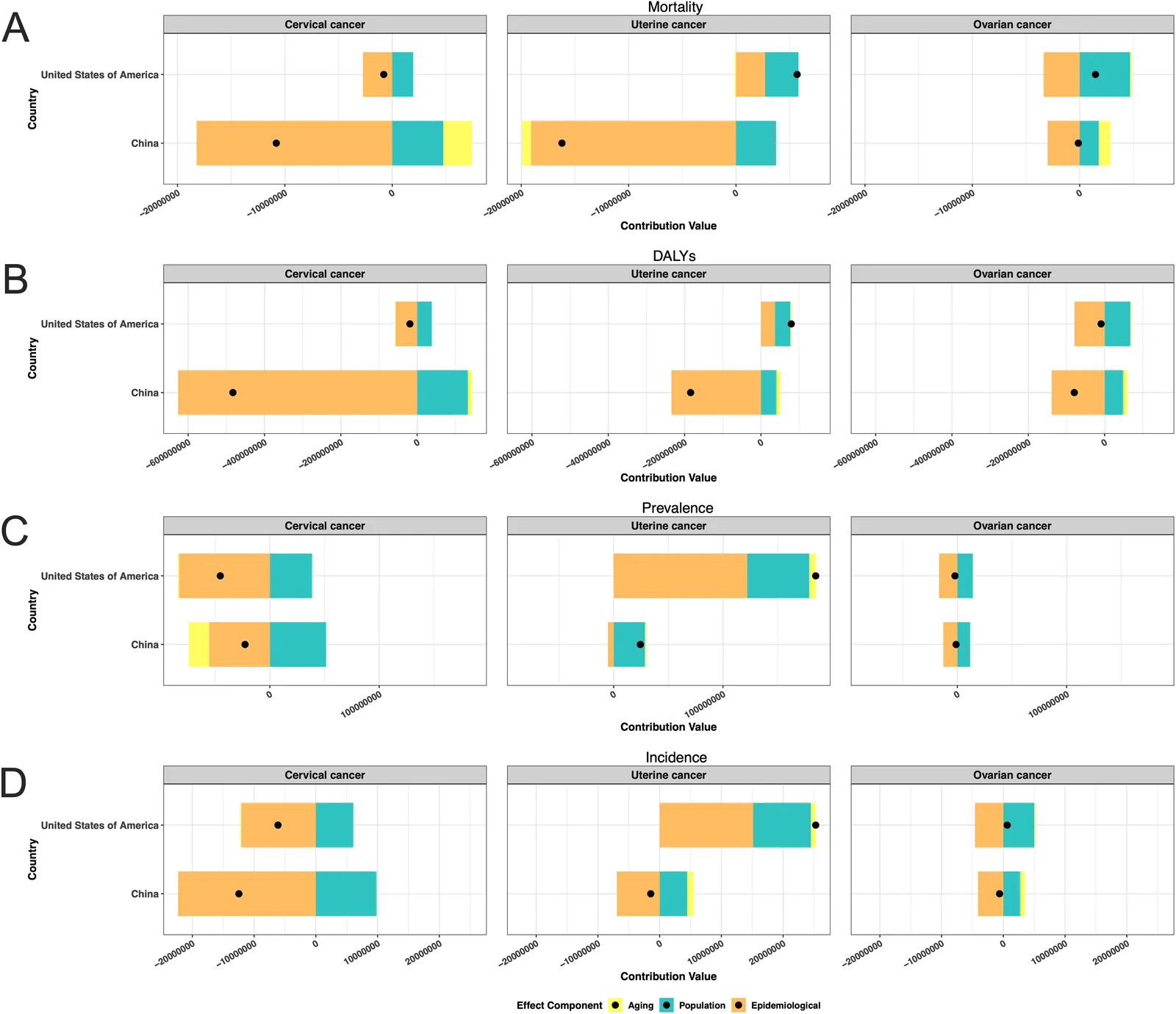
Decomposition of absolute changes in gynaecological cancer burden between 1990 and 2023. The panels illustrate the attribution of net absolute changes in (A) Mortality, (B) Disability-adjusted life years (DALYs), (C) Prevalence, and (D) Incidence for cervical, uterine, and ovarian cancers in China and the United States. Colored horizontal bars quantify the absolute contribution of population ageing, population growth, and epidemiological change. Bars extending to the right of the zero-axis indicate a positive contribution, whereas bars extending to the left indicate a negative contribution. The black dots represent the net overall change. U.S. uterine cancer illustrates how demographic and epidemiological components can point in the same direction.

In China, negative contributions from epidemiological-rate change offset the upward demographic components for most outcomes. Uterine-cancer prevalence was the main exception in the displayed estimates, with a small net increase associated with demographic change.

In the United States, cervical and ovarian outcomes showed combinations of demographic growth and favorable rate changes, whereas uterine cancer showed positive contributions from both population growth and epidemiological-rate change. Following Das Gupta’s framework, the decomposition describes arithmetic contributions to burden change; it does not establish the causes of the underlying rate trends nor propagate individual-level uncertainty.

The decomposition results provide independent population-level context for prioritization: they quantify the arithmetic contributions of population growth, ageing, and epidemiological-rate change to absolute burden differences, without attributing those changes to FAERS exposures.

### 3.10. Long-term forecasting of disease burden (2024–2053)

Bayesian age-period-cohort models were used to project 2024–2053 trajectories from the stated population projections, age weights, priors, and model structure. In the 2014–2023 holdout comparison, mean absolute percentage error ranged from 2.67% to 36.18% across the 24 location-cancer-measure series (S11 Table). The projections remain exploratory scenario estimates, and uncertainty widens over the 30-year horizon.

In China, projected age-standardized rates may decline while absolute burden remains substantial because of demographic structure (Fig 10A-L). The widening 95% uncertainty intervals over the 30-year horizon emphasize model and population-projection uncertainty rather than a deterministic future trajectory.

**Fig 10:**
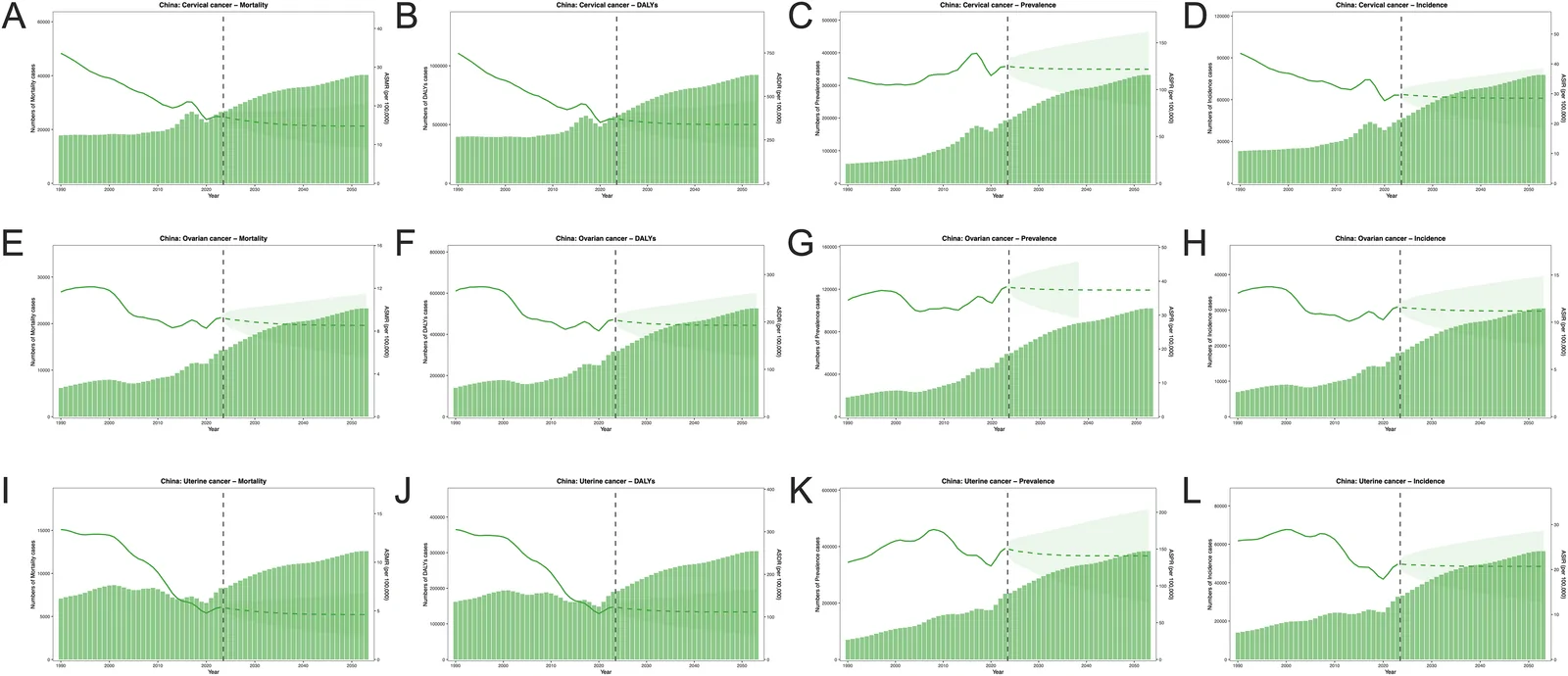
Exploratory Bayesian age-period-cohort projections of gynaecological cancer burden in China (2024–2053). Forecasts are presented for cervical cancer (A–D), ovarian cancer (E–H), and uterine cancer (I–L) across four metrics. Solid lines represent historical female GBD 2023 estimates from 1990–2023, dashed lines represent exploratory projections for 2024–2053, and shaded regions represent model-based 95% uncertainty intervals. The vertical line marks the 2023 boundary.

The United States presents a high projected uterine-cancer burden (S3 Fig), but the apparent smoothness of a modelled curve should not be interpreted as certainty. Long-horizon extrapolation is sensitive to population projections, age-period-cohort constraints, prior settings, and structural breaks. The forecast is most useful as a planning scenario that identifies where a validated signal could have greater public-health consequence, rather than a deterministic trajectory.

## 4. Discussion

The principal contribution of this study is the development of a transparent triage framework that explicitly separates FAERS reporting disproportionality from real-world GBD population burden. FAERS identifies drug–event hypotheses and their reporting context, whereas GBD describes independent, macro-level epidemiological trajectories. The 2021 ranitidine peak was compatible with stimulated reporting and included a substantial fraction of attorney-coded reports, but FAERS does not encode litigation status. Conversely, the rising US uterine-cancer trajectory identifies a public-health setting in which any subsequently validated pharmacological signal could have substantial consequences. The framework is intended to organize validation priorities, not to replace active-comparator pharmacoepidemiology.

It is imperative to note that causal interpretation requires a separate, rigorous evidentiary step. Because FAERS inherently lacks reliable denominators of exposed and unexposed populations, disproportionality metrics (ROR, PRR, EBGM, and IC) quantify reporting imbalance rather than clinical incidence or relative risk. The contingency tables remain highly sensitive to under-reporting, follow-up structure, comedication, confounding by indication, reverse causality (pre-existing disease), and differential surveillance. The phenomena of notoriety and stimulated reporting are particularly relevant for long-marketed products following safety communications, media attention, or litigation. Therefore, the present analysis strictly treats these metrics as signal-detection quantities, reserving definitive drug-attributable risk estimation and biological latency evaluation for longitudinal, controlled study designs. The distinction between signal detection, signal management, and causal validation is also emphasized in recent pharmacovigilance guidance [16–18].

Ranitidine illustrates why toxicological plausibility and reporting magnitude must be separated. The external multinational cohort study did not reproduce the same cancer association in its design, providing contextual evidence rather than direct validation or refutation of the FAERS signal. Differences in formulation, exposure ascertainment, follow-up timelines, and outcome definitions prevent a one-to-one comparison. The appropriate conclusion is that the FAERS disproportionality is hypothesis-generating and requires exposure-denominator studies. The FAERS-based ranitidine analysis, the multinational cohort study, and the randomized NDMA pharmacology trial provide complementary but non-equivalent evidence [25–27].

Tamoxifen provides a robust positive-control context because its specific endometrial pharmacology—acting as a partial estrogen agonist in the endometrium—is biologically plausible and well-supported by external clinical evidence. Concurrently, our analysis of the broader FAERS uterine cancer records found that most tamoxifen-associated reports also contained a breast cancer or anti-estrogen indication. This compatibility with known histological mechanisms does not, however, validate the FAERS effect size, which remains vulnerable to confounding by indication, surveillance bias, menopausal status, and survivorship [28].

Talc and zinc oxide illustrate product and reporting-channel ambiguity. Among ovarian-cancer reports, the talc group included 147 unique primary-suspect reports, with 59.9% consumer-coded and 14.3% lawyer-coded records; the zinc-oxide group included 152 reports and was predominantly consumer-coded. These consumer products involve inherent product-family misclassification and inconsistent exposure definitions; therefore, they should not be placed on the same causal interpretive level as prescription medicines. The extreme RORs are best treated as reporting-distortion hypotheses requiring independent exposure assessment. The epidemiological literature on talc and cancer remains sensitive to exposure definition and study design [29,30].

Findings for adalimumab and other immunosuppressants are presented exclusively as hypotheses requiring an external validation pathway. Given the complex clinical background of these patients, future validation studies must rigorously stratify by indication, combination therapy, prior cancer, reporter type, and calendar time to isolate potential pharmacological risks from underlying disease progression.

The FAERS–GBD juxtaposition contributes to prioritization in two ways. First, the sharp 2021 FAERS peak is not mirrored by a comparable abrupt change in the macro-level cancer series, which argues against interpreting the peak as a simple population-incidence surge and supports review of reporting dynamics, particularly late-stage stimulated reporting distinct from classical post-marketing trends. Second, the increasing US uterine-cancer trajectory indicates a setting in which a subsequently validated risk factor could have greater public-health consequence. The GBD layer changes the consequence of validation, not the causal credibility of the initial FAERS association. The broader gynecological-cancer burden literature documents substantial geographic heterogeneity and forward-looking burden differences [31,32].

China was included as a prespecified population comparator rather than as a denominator for the predominantly US FAERS reports. The small number of Chinese FAERS reports is reported directly and is not used to infer lower or higher national drug risk. This framework explicitly juxtaposes the FAERS system footprint against macro-population burden, rather than comparing Chinese and US exposure or incidence risks. The contrasting GBD trajectories illustrate why a candidate signal may warrant different follow-up priority in different health-system and demographic settings. Recent China-specific GBD analyses provide relevant population-level context without supplying an exposure denominator for FAERS [22].

The age-period-cohort (APC) and decomposition analyses further contextualize this population framework. The concentration of ovarian and uterine burdens in later life dictates that downstream validation studies must rigorously account for cumulative environmental exposures, polypharmacy, competing mortality risks, and age-dependent diagnostic intensity. Demographic transitions—specifically population growth and aging—can drive absolute case numbers upward even when age-standardized rates decline. For uterine cancer in the United States, the positive contributions of epidemiological and population-growth components, coupled with escalating period effects, demonstrate that demographic shifts alone are insufficient to account for the overall increase [9,23]. While such forecasts are essential for health service planning, their expanding confidence intervals and inherent sensitivity to model specifications render them the most uncertain analytical layer. Consequently, they should inform scenario planning rather than dictate definitive pharmacovigilance conclusions [24].

Methodological strengths include the 20-year observation window, explicit documentation of the deduplication workflow, explicit PT definitions, restriction of drug-level screening to primary-suspect records, four complementary signal algorithms, multiplicity sensitivity counts, reporter and product summaries, and preservation of the processed source outputs. The revised framework follows the central READUS-PV principle that disproportionality findings should be reported with their analytical unit, thresholds, uncertainty, and external-context limitations [11].

Several methodological constraints remain. FAERS lacks reliable exposure denominators and comprehensive longitudinal histories; the prespecified high-frequency top-50 design creates a deliberate selection boundary, which inherently limits our ability to detect rare signals associated with low-frequency or newly marketed medications; the signal tables use record-level drug–event units generated before the final clinical-consistency review; and residual confounding, product-family misclassification, stimulated reporting, and ecological fallacy remain possible. GBD estimates describe population trajectories rather than drug-exposed cohorts. TTO values are administratively reported intervals, and BAPC projections are scenario estimates. These boundaries define the appropriate next step—validation in longitudinal EHR or claims data—while preserving the value of the FAERS–GBD framework for structured hypothesis prioritization. Recent pharmacovigilance position statements and EHR signal-identification reviews support this staged validation pathway [16–18].

## 5. Conclusion

In conclusion, this study formalized a two-dimensional prioritization framework that successfully bridges spontaneous reporting signals with macro-epidemiological context. Evaluating FAERS and GBD in parallel highlights the critical distinction between reporting anomalies and true population-level burden. For instance, the 2021 ranitidine reporting excess in FAERS is best categorized as an administrative artifact compatible with stimulated reporting, rather than a sudden biological epidemic. Conversely, the rising US uterine-cancer trajectory identified via GBD represents a genuine macro-epidemiological shift, creating a setting in which any subsequently confirmed pharmacological risk factor would have magnified public-health consequences. Ultimately, to move from signal prioritization to causal validation, the hypotheses generated by our framework must transition to longitudinal electronic health record (EHR) or claims-based studies utilizing active comparators, incident users, indication adjustment, and prespecified latency windows [16–18].

## Supporting information

S1 Fig Age-period-cohort (APC) model estimates for ovarian cancer burden in China and the United States. Rows depict four epidemiological outcomes: Mortality, Disability-Adjusted Life Years (DALYs), Prevalence, and Incidence. Columns illustrate the distinct Age, Period, and Cohort effects derived from the APC framework. Red lines denote estimates for China, and blue lines denote the United States, with respective shaded areas indicating 95% confidence intervals (CIs). The y-axis for the age effect represents the absolute rate (per 100,000 population), whereas the y-axes for period and cohort effects represent the estimated Rate Ratio (RR). The horizontal dashed line at RR = 1.0 indicates the baseline reference risk level.
(TIFF)

S2 Fig Age-period-cohort (APC) model estimates for uterine cancer burden in China and the United States. Rows depict four epidemiological outcomes: Mortality, Disability-Adjusted Life Years (DALYs), Prevalence, and Incidence. Columns illustrate the distinct Age, Period, and Cohort effects derived from the APC framework. Red lines denote estimates for China, and blue lines denote the United States, with respective shaded areas indicating 95% confidence intervals (CIs). The y-axis for the age effect represents the absolute rate (per 100,000 population), whereas the y-axes for period and cohort effects represent the estimated Rate Ratio (RR). The horizontal dashed line at RR = 1.0 indicates the baseline reference risk level. Noticeable exponential increases in the U.S. period and cohort Rate Ratios (blue lines, middle and right columns) contrast sharply with the trends observed in China.
(TIFF)

S3 Fig Exploratory Bayesian age-period-cohort projections of gynaecological cancer burden in the United States (2024–2053). Forecasts are presented for cervical cancer (A-D), ovarian cancer (E-H), and uterine cancer (I-L) across four metrics: mortality, disability-adjusted life years (DALYs), prevalence, and incidence. Within each panel, solid green lines represent historical age-standardized rates (ASRs), while dashed green lines denote projected ASRs (right y-axis). Green bars indicate the absolute numbers of cases/events (left y-axis). The light green shaded areas represent the 95% uncertainty intervals for the projections. The vertical dashed black line marks the boundary between historical estimates (up to 2023) and future projections. Notably, projection uncertainty intervals are substantially narrower compared to the Chinese cohort.
(TIFF)

S1 Table FAERS records included in the analysis by cancer type. (DOCX)

S2 Table Shared FAERS reports across the cancer-specific datasets. (DOCX)

S3 Table MedDRA preferred terms and codes used to identify the cancer-specific reports. (DOCX)

S4 Table Peak reporting year and reporter type for selected product groups. (DOCX)

S5 Table Two-by-two contingency table used for disproportionality signal detection in FAERS. (DOCX)

S6 Table Formulas and prespecified thresholds for the four disproportionality algorithms. (DOCX)

S7 Table Classification of selected drug-cancer pairs using the F × G framework. (DOCX)

S8 Table Coverage of primary-suspect report-drug pairs by the 50 and 100 most frequently reported drugs. (DOCX)

S9 Table Drug-cancer pairs meeting the disproportionality and multiplicity criteria. (DOCX)

S10 Table Recorded treatment-to-event intervals by cancer type. (DOCX)

S11 Table Performance of log-linear projections in the 2014–2023 holdout period. (DOCX)
