# Supplementary material for: Gynecological malignancy signal triage: A two-dimensional framework integrating US-derived FAERS data with comparative US–China GBD trajectories
Source: PLoS One. 2026 Oct 29;21(10):e0361817. doi: 10.1371/journal.pone.0361817 (PMC13718977; doi:10.1371/journal.pone.0361817)
Supplement: Supplementary file 1 [file pone.0361817.s004.docx]

**S1 Table. FAERS records included in the analysis by cancer type.**

*Counts are shown for source records, unique report identifiers, primary-suspect drugs, reactions, and indications. Abbreviations: CC, cervical cancer; OC, ovarian cancer; UC, uterine cancer; PS, primary suspect.*

| **Cancer group** | **DEMO rows** | **Unique PRIMARYIDs** | **Unique CASEIDs** | **Duplicate PRIMARYID rows** | **Duplicate CASEID rows** | **PS drug rows** | **Unique PS report-drug pairs** | **Reports with a PS drug** | **Reports with multiple PS drugs** | **Unique PS generic names** | **REAC rows** | **INDI rows** |
| --- | --- | --- | --- | --- | --- | --- | --- | --- | --- | --- | --- | --- |
| CC | 3870 | 3870 | 3870 | 0 | 0 | 3871 | 3870 | 3870 | 0 | 526 | 20024 | 17974 |
| OC | 7636 | 7636 | 7636 | 0 | 0 | 7643 | 7636 | 7636 | 0 | 796 | 36515 | 32431 |
| UC | 8211 | 8211 | 8211 | 0 | 0 | 8216 | 8211 | 8211 | 0 | 799 | 37585 | 34736 |
