# Supplementary material for: Gynecological malignancy signal triage: A two-dimensional framework integrating US-derived FAERS data with comparative US–China GBD trajectories
Source: PLoS One. 2026 Oct 29;21(10):e0361817. doi: 10.1371/journal.pone.0361817 (PMC13718977; doi:10.1371/journal.pone.0361817)
Supplement: Supplementary file 2 [file pone.0361817.s005.docx]

**S2 Table. Shared FAERS reports across the cancer-specific datasets.**

*Reports meeting more than one cancer-specific preferred-term definition are counted in each applicable dataset. Abbreviations: CC, cervical cancer; OC, ovarian cancer; UC, uterine cancer.*

| **Comparison** | **Overlapping PRIMARYIDs** |
| --- | --- |
| CC-OC | 310 |
| CC-UC | 406 |
| OC-UC | 575 |
| CC-OC-UC | 105 |
