# Supplementary material for: Gynecological malignancy signal triage: A two-dimensional framework integrating US-derived FAERS data with comparative US–China GBD trajectories
Source: PLoS One. 2026 Oct 29;21(10):e0361817. doi: 10.1371/journal.pone.0361817 (PMC13718977; doi:10.1371/journal.pone.0361817)
Supplement: Supplementary file 3 [file pone.0361817.s006.docx]

**S3 Table. MedDRA preferred terms and codes used to identify the cancer-specific reports.**

*The uterine cancer definition includes uterine and endometrial cancer terms. MedDRA codes are retained from the original submission; recurrence, metastatic, and stage-specific terms are identified separately. Abbreviations: CC, cervical cancer; OC, ovarian cancer; UC, uterine cancer.*

| **Cancer group** | **MedDRA preferred term** | **MedDRA code** | **Unique reports** | **REAC rows** | **Recurrence term** | **Metastatic term** | **Stage-specific term** | **Scope note** |
| --- | --- | --- | --- | --- | --- | --- | --- | --- |
| CC | CERVIX CARCINOMA STAGE IV | 10008348 | 112 | 129 | No | No | Yes | Site-specific PT set |
| CC | CERVIX CARCINOMA STAGE III | 10008347 | 110 | 127 | No | No | Yes | Site-specific PT set |
| CC | CERVIX CARCINOMA STAGE II | 10008346 | 91 | 107 | No | No | Yes | Site-specific PT set |
| CC | CERVIX CARCINOMA STAGE I | 10008345 | 142 | 169 | No | No | Yes | Site-specific PT set |
| CC | CERVIX CARCINOMA STAGE 0 | 10061809 | 244 | 315 | No | No | Yes | Site-specific PT set |
| CC | CERVIX CARCINOMA RECURRENT | 10008344 | 55 | 81 | Yes | No | No | Site-specific PT set |
| CC | CERVIX CARCINOMA | 10008342 | 3125 | 4021 | No | No | No | Site-specific PT set |
| OC | OVARIAN CANCER STAGE IV | 10070908 | 332 | 393 | No | No | Yes | Site-specific PT set |
| OC | OVARIAN CANCER STAGE III | 10070907 | 264 | 326 | No | No | Yes | Site-specific PT set |
| OC | OVARIAN CANCER STAGE II | 10070906 | 100 | 107 | No | No | Yes | Site-specific PT set |
| OC | OVARIAN CANCER STAGE I | 10070905 | 190 | 217 | No | No | Yes | Site-specific PT set |
| OC | OVARIAN CANCER RECURRENT | 10066697 | 383 | 500 | Yes | No | No | Site-specific PT set |
| OC | OVARIAN CANCER METASTATIC | 10057529 | 502 | 669 | No | Yes | No | Site-specific PT set |
| OC | OVARIAN CANCER | 10033128 | 5921 | 7526 | No | No | No | Site-specific PT set |
| OC | HOMOLOGOUS RECOMBINATION DEFICIENCY POSITIVE ADVANCED OVARIAN CANCER | 10084789 | 1 | 1 | No | No | No | Site-specific PT set |
| OC | HER2 POSITIVE OVARIAN CANCER | 10089166 | 0 | 0 | No | No | No | Site-specific PT set |
| UC | UTERINE CANCER | 10046766 | 6279 | 7607 | No | No | No | Uterine category includes endometrial terms |
| UC | METASTATIC UTERINE CANCER | 10068116 | 81 | 122 | No | Yes | No | Uterine category includes endometrial terms |
| UC | HER2 POSITIVE ENDOMETRIAL CANCER | 10089167 | 0 | 0 | No | No | No | Uterine category includes endometrial terms |
| UC | ENDOMETRIAL CANCER STAGE IV | 10014741 | 19 | 25 | No | No | Yes | Uterine category includes endometrial terms |
| UC | ENDOMETRIAL CANCER STAGE III | 10014740 | 57 | 68 | No | No | Yes | Uterine category includes endometrial terms |
| UC | ENDOMETRIAL CANCER STAGE II | 10014739 | 35 | 40 | No | No | Yes | Uterine category includes endometrial terms |
| UC | ENDOMETRIAL CANCER STAGE I | 10014738 | 136 | 159 | No | No | Yes | Uterine category includes endometrial terms |
| UC | ENDOMETRIAL CANCER STAGE 0 | 10014737 | 4 | 6 | No | No | Yes | Uterine category includes endometrial terms |
| UC | ENDOMETRIAL CANCER RECURRENT | 10014736 | 45 | 63 | Yes | No | No | Uterine category includes endometrial terms |
| UC | ENDOMETRIAL CANCER METASTATIC | 10014734 | 105 | 145 | No | Yes | No | Uterine category includes endometrial terms |
| UC | ENDOMETRIAL CANCER | 10014733 | 1550 | 2056 | No | No | No | Uterine category includes endometrial terms |
