# Supplementary material for: Gynecological malignancy signal triage: A two-dimensional framework integrating US-derived FAERS data with comparative US–China GBD trajectories
Source: PLoS One. 2026 Oct 29;21(10):e0361817. doi: 10.1371/journal.pone.0361817 (PMC13718977; doi:10.1371/journal.pone.0361817)
Supplement: Supplementary file 4 [file pone.0361817.s007.docx]

**S4 Table. Peak reporting year and reporter type for selected product groups.**

*Peak-year shares and reporter-type proportions are calculated among unique reports for each cancer and product group. FAERS does not include a litigation-status field. Abbreviations: CC, cervical cancer; OC, ovarian cancer; UC, uterine cancer.*

| **Cancer group** | **Product family** | **Unique reports** | **Peak year** | **Peak-year reports** | **Peak-year share (%)** | **Attorney-coded reports** | **Attorney-coded share (%)** | **Consumer-coded share (%)** |
| --- | --- | --- | --- | --- | --- | --- | --- | --- |
| CC | ranitidine | 1573 | 2021 | 1093 | 69.4851 | 659 | 41.8945 | 32.1043 |
| CC | talc | 1 | 2018 | 1 | 100 | 0 | 0 | 100 |
| CC | zinc oxide | 21 | 2020 | 7 | 33.3333 | 0 | 0 | 100 |
| CC | tamoxifen | 4 | 2018 | 1 | 25 | 1 | 25 | 25 |
| CC | adalimumab | 107 | 2017 | 18 | 16.8224 | 0 | 0 | 65.4206 |
| CC | infliximab | 83 | 2020 | 11 | 13.253 | 0 | 0 | 21.6867 |
| OC | ranitidine | 2735 | 2021 | 1911 | 69.872 | 990 | 36.1974 | 35.5759 |
| OC | talc | 147 | 2018 | 49 | 33.3333 | 21 | 14.2857 | 59.8639 |
| OC | zinc oxide | 152 | 2020 | 54 | 35.5263 | 2 | 1.31579 | 96.7105 |
| OC | tamoxifen | 19 | 2010 | 4 | 21.0526 | 0 | 0 | 21.0526 |
| OC | adalimumab | 148 | 2018 | 21 | 14.1892 | 0 | 0 | 55.4054 |
| OC | infliximab | 65 | 2020 | 7 | 10.7692 | 0 | 0 | 32.3077 |
| UC | ranitidine | 3970 | 2021 | 2852 | 71.8388 | 1437 | 36.1965 | 35.4912 |
| UC | talc | 6 | 2014 | 2 | 33.3333 | 0 | 0 | 66.6667 |
| UC | zinc oxide | 18 | 2020 | 7 | 38.8889 | 0 | 0 | 100 |
| UC | tamoxifen | 92 | 2007 | 15 | 16.3043 | 0 | 0 | 16.3043 |
| UC | adalimumab | 167 | 2020 | 25 | 14.9701 | 0 | 0 | 73.0539 |
| UC | infliximab | 86 | 2022 | 12 | 13.9535 | 0 | 0 | 26.7442 |
