# Supplementary material for: Gynecological malignancy signal triage: A two-dimensional framework integrating US-derived FAERS data with comparative US–China GBD trajectories
Source: PLoS One. 2026 Oct 29;21(10):e0361817. doi: 10.1371/journal.pone.0361817 (PMC13718977; doi:10.1371/journal.pone.0361817)
Supplement: Supplementary file 5 [file pone.0361817.s008.docx]

**S5 Table. Two-by-two contingency table used for disproportionality signal detection in FAERS.**

*The symbols a, b, c, and d define the report counts used by the four disproportionality algorithms in S6 Table.*

| **Target Adverse Events (Cervical, Uterine, and Ovarian Cancers)** | **All Other Adverse Events** | **Total** |  |
| --- | --- | --- | --- |
| Target Drug | a | b | a + b |
| All Other Drugs | c | d | c + d |
| Total | a + c | b + d | a + b + c + d |

a: Number of reports for the target drug associated with the target cancer event.

b: Number of reports for the target drug associated with all other adverse events.

c: Number of reports for all other drugs associated with the target cancer event.

d: Number of reports for all other drugs associated with all other adverse events.
