# Supplementary material for: Gynecological malignancy signal triage: A two-dimensional framework integrating US-derived FAERS data with comparative US–China GBD trajectories
Source: PLoS One. 2026 Oct 29;21(10):e0361817. doi: 10.1371/journal.pone.0361817 (PMC13718977; doi:10.1371/journal.pone.0361817)
Supplement: Supplementary file 6 [file pone.0361817.s009.docx]

**S6 Table. Formulas and prespecified thresholds for the four disproportionality algorithms.**

*Threshold operators match the prespecified criteria in Section 2.4. For ROR and PRR, 0.5 was added to all four cells when any contingency-table cell was zero. IC025 and EBGM05 denote the lower 95% credibility bounds.*

| **Method** | **Formula** | **Threshold Criteria** |
| --- | --- | --- |
| ROR | $ROR=\frac{a/c}{b/d}=\frac{ad}{bc}$ $SE\left( \ln ROR \right)=\sqrt{\frac{1}{a}+\frac{1}{b}+\frac{1}{c}+\frac{1}{d}}$ $95\%CI=e^{\ln\left( ROR \right)\pm1.96\sqrt{\frac{1}{a}+\frac{1}{b}+\frac{1}{c}+\frac{1}{d}}}$ | $a\geq3$ and Lower limit of 95% CI ≥ 1 |
| PRR | $PRR=\frac{a/\left( a+b \right)}{c/\left( c+d \right)}$ $\chi^{2}=\sum\frac{\left( O-E \right)^{2}}{E}$ $E=\frac{\left( a+b \right)\left( a+c \right)}{\left( a+b+c+d \right)}$; $O=a$ | $a\geq3$ and PRR $\geq$ 2 and $\chi^{2}\geq4$ |
| IC (BCPNN) | $IC=\log_{2}\frac{a\left( a+b+c+d \right)}{\left( a+b \right)\left( a+c \right)}$ $IC_{025}=E\left( IC \right)-2SD$ | $IC_{025}\geq0$ |
| EBGM | $EBGM=\frac{a/\left( a+b+c+d \right)}{\left( a+c \right)\left( a+b \right)}$ $95\%CI=e^{\ln\left( EBGM \right)\pm1.96\sqrt{\frac{1}{a}+\frac{1}{b}+\frac{1}{c}+\frac{1}{d}}}$ | $EBGM_{05}\geq2$ |

Abbreviations: BCPNN, Bayesian confidence propagation neural network; CI, confidence interval; EBGM, empirical Bayes geometric mean; IC, information component; PRR, proportional reporting ratio; ROR, reporting odds ratio; SD, standard deviation.
