# Supplementary material for: Gynecological malignancy signal triage: A two-dimensional framework integrating US-derived FAERS data with comparative US–China GBD trajectories
Source: PLoS One. 2026 Oct 29;21(10):e0361817. doi: 10.1371/journal.pone.0361817 (PMC13718977; doi:10.1371/journal.pone.0361817)
Supplement: Supplementary file 7 [file pone.0361817.s010.docx]

**S7 Table. Classification of selected drug-cancer pairs using the F×G framework.**

*F scores summarize FAERS signal characteristics, and G scores summarize the corresponding US incidence trajectory. Priority categories follow the definitions in Section 2.4. Abbreviations: CC, cervical cancer; OC, ovarian cancer; UC, uterine cancer.*

| **Cancer group** | **Drug** | **Target reports (a)** | **ROR** | **ROR lower 95% CI** | **ROR upper 95% CI** | **PRR** | **PRR lower 95% CI** | **PRR upper 95% CI** | **EBGM** | **EBGM05** | **IC** | **IC025** | **P value** | **BH-FDR q value** | **Bonferroni-adjusted P** | **Positive algorithms (n)** | **Artifact flag** | **Artifact rationale** | **FAERS score** | **GBD score** | **US incidence EAPC (%)** | **US incidence ASR, 2023** |
| --- | --- | --- | --- | --- | --- | --- | --- | --- | --- | --- | --- | --- | --- | --- | --- | --- | --- | --- | --- | --- | --- | --- |
| CC | Ranitidine | 1802 | 55.47 | 52.44 | 58.68 | 55.31 | 52.3 | 58.51 | 37.7 | 35.63 | 5.24 | 5.13 | <1e-300 | 1.15385e-299 | <1e-300 | 4 | Yes | single-year concentration >=50%; attorney-coded share >=40% | F2 | G0 | -1.67283 | 14.604 |
| OC | Talc | 360 | 10364.4 | 8673.61 | 12384.7 | 3518.84 | 3305.78 | 3745.64 | 3429.19 | 2869.78 | 11.74 | 8.15 | <1e-300 | 1.15385e-299 | <1e-300 | 4 | Yes | consumer-product/product-family ambiguity | F2 | G0 | -1.43476 | 8.76319 |
| OC | Zinc oxide | 216 | 2412.14 | 2051.66 | 2835.96 | 1655.16 | 1480.22 | 1850.76 | 1629.86 | 1386.29 | 10.67 | 7.36 | <1e-300 | 1.15385e-299 | <1e-300 | 4 | No |  | F3 | G0 | -1.43476 | 8.76319 |
| UC | Tamoxifen | 89 | 37.77 | 30.64 | 46.56 | 37.54 | 30.49 | 46.21 | 37.28 | 30.24 | 5.22 | 4.43 | 1.41159e-104 | 1.11441e-103 | 2.11739e-102 | 4 | No |  | F3 | G2 | 1.66974 | 34.1417 |
| CC | Adalimumab | 242 | 1.19 | 1.05 | 1.35 | 1.19 | 1.05 | 1.35 | 1.18 | 1.04 | 0.24 | 0.05 | 0.00909405 | 0.016435 | 1 | 2 | No |  | F2 | G0 | -1.67283 | 14.604 |
| OC | Adalimumab | 322 | 0.61 | 0.55 | 0.68 | 0.61 | 0.55 | 0.68 | 0.62 | 0.55 | -0.69 | -0.85 | 7.88173e-19 | 3.03144e-18 | 1.18226e-16 | 0 | No |  | F0 | G0 | -1.43476 | 8.76319 |
| UC | Adalimumab | 371 | 0.79 | 0.72 | 0.88 | 0.79 | 0.72 | 0.88 | 0.8 | 0.72 | -0.32 | -0.47 | 1.33475e-05 | 3.64022e-05 | 0.00200212 | 0 | No |  | F0 | G2 | 1.66974 | 34.1417 |
