# Supplementary material for: Gynecological malignancy signal triage: A two-dimensional framework integrating US-derived FAERS data with comparative US–China GBD trajectories
Source: PLoS One. 2026 Oct 29;21(10):e0361817. doi: 10.1371/journal.pone.0361817 (PMC13718977; doi:10.1371/journal.pone.0361817)
Supplement: Supplementary file 8 [file pone.0361817.s011.docx]

**S8 Table. Coverage of primary-suspect report-drug pairs by the 50 and 100 most frequently reported drugs.**

*Coverage is the percentage of all primary-suspect report-drug pairs represented by the 50 or 100 most frequently reported drugs. Abbreviations: CC, cervical cancer; OC, ovarian cancer; UC, uterine cancer; PS, primary suspect.*

| **Cancer group** | **Unique PS drugs** | **Eligible drugs (>=3 reports)** | **Top-50 cutoff (reports)** | **Top-50 coverage (%)** | **Top-100 coverage (%)** | **Eligible drugs outside top 50** |
| --- | --- | --- | --- | --- | --- | --- |
| CC | 526 | 158 | 9 | 75.7881 | 83.3075 | 108 |
| OC | 796 | 280 | 18 | 72.8261 | 80.5657 | 230 |
| UC | 799 | 275 | 19 | 75.1309 | 82.5478 | 225 |
