# Supplementary material for: Gynecological malignancy signal triage: A two-dimensional framework integrating US-derived FAERS data with comparative US–China GBD trajectories
Source: PLoS One. 2026 Oct 29;21(10):e0361817. doi: 10.1371/journal.pone.0361817 (PMC13718977; doi:10.1371/journal.pone.0361817)
Supplement: Supplementary file 9 [file pone.0361817.s012.docx]

**S9 Table. Drug-cancer pairs meeting the disproportionality and multiplicity criteria.**

*Counts are reported for the four disproportionality algorithms and for Benjamini-Hochberg and Bonferroni multiplicity adjustments. Abbreviations: CC, cervical cancer; OC, ovarian cancer; UC, uterine cancer.*

| **Cancer group** | **Tested pairs** | **Positive by >=1 algorithm** | **Positive by >=2 algorithms** | **Positive by >=3 algorithms** | **Positive by all 4 algorithms** | **BH-FDR q<0.05** | **Bonferroni-adjusted P<0.05** | **Pairs with a zero cell** |
| --- | --- | --- | --- | --- | --- | --- | --- | --- |
| CC | 50 | 24 | 24 | 18 | 14 | 27 | 21 | 0 |
| OC | 50 | 19 | 19 | 16 | 16 | 32 | 26 | 0 |
| UC | 50 | 20 | 20 | 16 | 12 | 29 | 22 | 0 |
