# Supplementary material for: Gynecological malignancy signal triage: A two-dimensional framework integrating US-derived FAERS data with comparative US–China GBD trajectories
Source: PLoS One. 2026 Oct 29;21(10):e0361817. doi: 10.1371/journal.pone.0361817 (PMC13718977; doi:10.1371/journal.pone.0361817)
Supplement: Supplementary file 10 [file pone.0361817.s013.docx]

**S10 Table. Recorded treatment-to-event intervals by cancer type.**

*Intervals were calculated from recorded treatment start and event dates. Missing, non-positive, and intervals exceeding 3,600 days were excluded. Abbreviations: CC, cervical cancer; OC, ovarian cancer; UC, uterine cancer.*

| **Cancer group** | **Source rows** | **Unique source PRIMARYIDs** | **Missing/non-numeric intervals** | **Non-positive intervals** | **Intervals >3,600 days** | **Valid unique reports** | **Valid fraction of cohort (%)** | **Median (days)** | **Q1 (days)** | **Q3 (days)** | **Intervals >360 days (%)** | **Minimum (days)** | **Maximum (days)** |
| --- | --- | --- | --- | --- | --- | --- | --- | --- | --- | --- | --- | --- | --- |
| CC | 618 | 618 | 0 | 0 | 27 | 591 | 15.2713 | 427 | 122.5 | 1096 | 55.1607 | 1 | 3597 |
| OC | 1321 | 1321 | 0 | 0 | 188 | 1133 | 14.8376 | 546 | 111 | 1461 | 58.2524 | 1 | 3592 |
| UC | 1175 | 1175 | 0 | 0 | 103 | 1072 | 13.0557 | 610 | 207 | 1453.5 | 63.7127 | 1 | 3577 |
