# Supplementary material for: Gynecological malignancy signal triage: A two-dimensional framework integrating US-derived FAERS data with comparative US–China GBD trajectories
Source: PLoS One. 2026 Oct 29;21(10):e0361817. doi: 10.1371/journal.pone.0361817 (PMC13718977; doi:10.1371/journal.pone.0361817)
Supplement: Supplementary file 11 [file pone.0361817.s014.docx]

**S11 Table. Performance of log-linear projections in the 2014-2023 holdout period.**

*Models were fitted to data from 1990-2013 and evaluated against observed age-standardized rates from 2014-2023.*

| **Location** | **Cancer** | **Measure** | **Training period** | **Holdout period** | **Log-linear MAE (ASR)** | **Log-linear MAPE (%)** |
| --- | --- | --- | --- | --- | --- | --- |
| China | Cervical cancer | DALYs (Disability-Adjusted Life Years) | 1990-2013 | 2014-2023 | 6.64575 | 4.54883 |
| China | Cervical cancer | Deaths | 1990-2013 | 2014-2023 | 0.244599 | 5.29488 |
| China | Cervical cancer | Incidence | 1990-2013 | 2014-2023 | 0.465926 | 3.66832 |
| China | Cervical cancer | Prevalence | 1990-2013 | 2014-2023 | 2.06649 | 2.67095 |
| China | Ovarian cancer | DALYs (Disability-Adjusted Life Years) | 1990-2013 | 2014-2023 | 7.33469 | 12.1224 |
| China | Ovarian cancer | Deaths | 1990-2013 | 2014-2023 | 0.229139 | 11.6391 |
| China | Ovarian cancer | Incidence | 1990-2013 | 2014-2023 | 0.433124 | 12.3539 |
| China | Ovarian cancer | Prevalence | 1990-2013 | 2014-2023 | 2.12591 | 12.5601 |
| China | Uterine cancer | DALYs (Disability-Adjusted Life Years) | 1990-2013 | 2014-2023 | 11.7534 | 33.9156 |
| China | Uterine cancer | Deaths | 1990-2013 | 2014-2023 | 0.382196 | 34.3282 |
| China | Uterine cancer | Incidence | 1990-2013 | 2014-2023 | 2.03058 | 33.3186 |
| China | Uterine cancer | Prevalence | 1990-2013 | 2014-2023 | 17.1034 | 36.1816 |
| United States of America | Cervical cancer | DALYs (Disability-Adjusted Life Years) | 1990-2013 | 2014-2023 | 9.41273 | 9.1353 |
| United States of America | Cervical cancer | Deaths | 1990-2013 | 2014-2023 | 0.18421 | 6.37336 |
| United States of America | Cervical cancer | Incidence | 1990-2013 | 2014-2023 | 3.14493 | 20.16 |
| United States of America | Cervical cancer | Prevalence | 1990-2013 | 2014-2023 | 26.1717 | 22.102 |
| United States of America | Ovarian cancer | DALYs (Disability-Adjusted Life Years) | 1990-2013 | 2014-2023 | 15.4855 | 10.6955 |
| United States of America | Ovarian cancer | Deaths | 1990-2013 | 2014-2023 | 0.792571 | 14.1379 |
| United States of America | Ovarian cancer | Incidence | 1990-2013 | 2014-2023 | 1.18714 | 12.8143 |
| United States of America | Ovarian cancer | Prevalence | 1990-2013 | 2014-2023 | 5.00384 | 11.9086 |
| United States of America | Uterine cancer | DALYs (Disability-Adjusted Life Years) | 1990-2013 | 2014-2023 | 14.7032 | 14.1289 |
| United States of America | Uterine cancer | Deaths | 1990-2013 | 2014-2023 | 0.588644 | 15.6085 |
| United States of America | Uterine cancer | Incidence | 1990-2013 | 2014-2023 | 2.26419 | 7.02395 |
| United States of America | Uterine cancer | Prevalence | 1990-2013 | 2014-2023 | 15.6908 | 6.07436 |
