# Supplementary material for: Gynecological malignancy signal triage: A two-dimensional framework integrating US-derived FAERS data with comparative US–China GBD trajectories
Source: PLoS One. 2026 Oct 29;21(10):e0361817. doi: 10.1371/journal.pone.0361817 (PMC13718977; doi:10.1371/journal.pone.0361817)
Supplement: Supplementary file 12 [file pone.0361817.s015.docx]

# Response to Reviewers

**A two-dimensional prioritization framework integrating US-dominant FAERS reporting with China–US GBD trajectories: gynecological malignancy signal triage**

*Manuscript ID: PONE-S-26-58127*

**Dear Academic Editor and Reviewers,**

We would like to express our deepest gratitude to the Academic Editor and the esteemed Reviewers for their **exceptionally meticulous, constructive, and methodologically astute evaluation of our manuscript**. Your profound insights have been instrumental in refining the scope of our FAERS analysis, formally structuring the intersection between spontaneous reporting signals and population-level disease burden, and strictly recalibrating our language to ensure our conclusions perfectly align with the evidentiary limits of secondary ecological data.

Guided by your precise critiques, we have undertaken a massive and fundamental overhaul of the manuscript (**with over 80% of the text thoroughly rewritten and restructured**). Every revision has been meticulously cross-validated against the raw FAERS extracts, processed signal matrices, GBD 2023 datasets, and structural audit outputs to ensure absolute transparency and reproducibility.

***Summary of Major Academic Revisions:***

In response to your combined feedback, we have fundamentally upgraded the scientific rigor of our study. The major structural and conceptual enhancements include:

**1. Formalization of the Two-Dimensional Prioritization Framework**: We explicitly conceptualized the framework as a matrix intersecting a FAERS evidence axis (F0–F3) with a GBD epidemiological context axis (G0–G2). The framework is now strictly repositioned as a structured triage tool for hypothesis prioritization, actively stripping away any unintended claims of causal validation.

**2. Transparency in Data Accounting and Analytical Units**: We systematically distinguished cohort-level deduplicated patient records from the drug–event pairs utilized in disproportionality tables. Furthermore, the “Top-50” analysis is now explicitly justified as a focused, high-frequency candidate screen, supported by stepwise data-cleaning flowcharts and zero-cell handling rationales.

**3. Recalibration of TTO and Weibull Interpretations**: We corrected the interpretation of Time-to-Onset (TTO) and Weibull shape parameters, strictly defining them as descriptive metrics of reporting timing distributions and signal plausibility, rather than representing true carcinogenic latency or clinical hazard functions.

**4. Contextualization of Artifacts and Specific Signals**: Findings related to ranitidine, talc, zinc oxide, immunosuppressants, as well as China–US geographical comparisons and long-term GBD forecasts, are now explicitly framed as hypothesis-generating evidence. We introduced an “artifact downgrade rule” to account for stimulated or litigation-driven reporting anomalies, emphasizing that these signals require subsequent validation in longitudinal EHR or claims-based studies.

***Response to Journal Requirements (Administrative and Formatting Updates):***

In addition to the academic revisions, we have carefully addressed all formatting and administrative requirements outlined by the journal office:

**1. Formatting & Style**: We have rigorously reviewed the manuscript against PLOS ONE’s style templates to ensure full compliance, including proper file naming conventions and title page formatting.

**2. Ethics Statement**: As requested, the ethics statement has been consolidated and now appears exclusively in the Methods section. It has been permanently removed from all other sections.

**3. Figure Quality (Figures 1–10, S1-3 Figures)**: We have generated and uploaded entirely new, high-resolution copies all figures (600dpi, TIFF format) to ensure maximum clarity and full compliance with PLOS ONE’s figure guidelines.

**4. Supporting Information**: Captions for all Supporting Information files have been correctly placed at the end of the manuscript, and all corresponding in-text citations have been updated and verified to match accordingly.

**5. Recommended references**: We carefully evaluated the specific publications recommended by the reviewers. Finding them highly relevant to strengthening our theoretical framework, we have seamlessly integrated and cited them within the revised Introduction and Discussion sections.

For maximum transparency, each reviewer’s comment is reproduced verbatim below. Our responses follow a structured format detailing: (1) a concise rationale for the modification; and (2) the exact revised text incorporated into the updated manuscript.

We sincerely hope that this comprehensively upgraded manuscript (＞80% text revised) now meets the high standards of the journal and addresses all your valuable concerns.

# Reviewer #1

We sincerely thank Reviewer #1 for the **meticulous, insightful, and highly constructive evaluation** of our manuscript. Your precise methodological critiques were exactly what this paper needed to reach its full potential. By closely following your guidance, we have executed a major revision, **substantially rewriting and restructuring approximately 80% of the main text**, with a particular focus on the Methods and Discussion sections. We firmly believe that your feedback has fundamentally elevated the scientific rigor, epidemiological accuracy, and overall transparency of our work.

Rather than making superficial text edits, your comments prompted us to deeply refine our core analytical framework. Broadly, we have implemented major upgrades across three primary domains in direct response to your feedback:

1. **Formalizing the Prioritization Framework & Data Reproducibility (*Comments 1–3)*:** We transformed the previously conceptual FAERS-GBD integration into a mathematically explicit, reproducible “Two-Dimensional Prioritization Matrix (F×G).” Furthermore, we completely rewrote the data deduplication workflow and clearly justified our top-50 screening boundary, ensuring that independent researchers can trace and reconstruct our exact analytical pipeline.
2. **Refining Epidemiological Rigor and Terminology *(Comments 4, 8, 10)*:** We systematically removed all overreaching causal language. For instance, we reframed Time-to-Onset (TTO) strictly as an “administrative reporting interval” rather than implying biological “carcinogenesis.” We also clarified the critical anatomical and histological distinctions between the broader GBD “Uterine” label and the FAERS “Endometrial” component, ensuring that macro-population trends are not conflated with drug-specific effects.
3. **Addressing Structural Biases and Observational Artifacts *(Comments 5, 7, 9)*:** We explicitly addressed the impact of litigation-driven stimulated reporting (e.g., ranitidine) and confounding by indication (e.g., tamoxifen). These observational realities are now formally managed through an “artifact penalty” within our scoring system, reinforcing FAERS as a hypothesis-generation tool rather than a platform for causal inference.

**We deeply appreciate the time and expertise you invested in guiding this manuscript.**

Below, we provide a detailed, point-by-point response outlining how each of your recommendations was addressed and integrated into the revised text.

## Comment 1

**Reviewer comment**

1.The central novelty of the manuscript is claimed to be a framework integrating FAERS disproportionality signals with GBD epidemiological trajectories. However, no explicit mathematical or decision rule is provided to determine how a signal is actually prioritized. The authors should clearly define how the two dimensions are calculated and combined.

### Response

We sincerely thank the reviewer for this highly constructive critique. We completely agree that while our original manuscript conceptualized the integration of FAERS and GBD data, it lacked an operationalized decision rule. Your feedback correctly identified that a formal, reproducible matrix is essential to substantiate the central novelty of our framework.

To address this, we have now established and explicitly defined a Two-Dimensional Prioritization Matrix (F×G) to serve as the rigorous decision rule for signal triage. Importantly, we emphasize that this matrix assigns validation priority from a public health perspective; it is not a composite score of causal credibility, nor does it conflate population burden with exposure denominators.

Specifically, we have operationalized the two dimensions as follows:

1. **The FAERS Dimension (F-score, graded F0–F3)**: Evaluates report-level evidence, algorithmic concordance (using ROR, PRR, EBGM, and IC), and case counts. To address the notorious biases in spontaneous reporting, we introduced a pre-specified “artifact penalty.” If a signal triggers an artifact flag (e.g., massive single-year concentration, disproportionately high attorney-initiated reporting, or product-family ambiguity), its F-grade is automatically downgraded by one level.
2. **The GBD Dimension (G-score, graded G0–G2)**: Evaluates the independent population burden trajectory based on the direction and statistical stability of the age-standardized rates (ASR).
3. **The Integration Rule (F×G Matrix)**: The cross-tabulation of these scores yields discrete priority tiers ranging from “Highest Validation Priority” (e.g., F3–G2, representing strong FAERS concordance superimposed on a worsening epidemiological burden) to “Artifact-focused Review” (for litigation-driven signals).

To ensure clarity and reproducibility, we have introduced the concept of the framework in the revised Introduction and fully detailed the mathematical and grading rules in a newly restructured Methods Section (Section 2.4). Furthermore, we have provided worked examples for ranitidine, talc, tamoxifen, and adalimumab in the Supporting Information to demonstrate the matrix in action.

**Revised manuscript —**

**Introduction:**

…

Superimposed on this clinical heterogeneity, evaluating suspected drug-induced malignancies within spontaneous reporting systems (SRSs) presents formidable analytical challenges. An adverse event report capturing a malignancy following drug exposure may represent a true biological hazard; alternatively, it may reflect severe confounding by indication, underlying disease progression, heightened diagnostic vigilance, or stimulated reporting driven by media coverage and mass litigation. Although large databases such as the FDA Adverse Event Reporting System (FAERS) offer high statistical power to detect unusual reporting clusters, standard quantitative signal detection algorithms (e.g., ROR, PRR, EBGM, and IC) merely measure relative disproportionality within the database. They mathematically cannot represent clinical incidence or relative risk, and are inherently vulnerable to distortion from masking effects and notoriety bias [11-14]. Therefore, utilizing spontaneous reporting databases for safety hypothesis generation is valuable only when these structural constraints are systematically integrated into signal interpretation [15].

To operationalize effective safety-signal triage, it is imperative to separate statistical disproportionality from public-health priority. To this end, we constructed a two-dimensional prioritization matrix (F×G) that cross-links micro-level drug safety signals (the FAERS dimension) with macro-level epidemiological trajectories (the GBD dimension). Recognizing the inherent limitations of spontaneous reporting, our framework avoids generating a flawed “causal score” and instead functions strictly as a structured triage matrix. It evaluates algorithmic concordance and explicitly penalizes known litigation artifacts on the FAERS axis, while independently assessing the context of the disease burden on the GBD axis. Ultimately, this framework does not alter or prove the causal credibility of a signal; rather, it dictates validation priority. It ensures that robust signals emerging within worsening epidemiological contexts (such as the surging uterine cancer baseline in the US) are prioritized for immediate pharmacoepidemiological attention.

**Methods 2.4:**

**2.4 Disproportionality Algorithms and the Two-Dimensional Prioritization Matrix (F×G)**

To mitigate the statistical noise and inflated false-discovery rates characteristic of the low-frequency “long tail” in FAERS, our primary disproportionality analysis was restricted to a prespecified high-frequency candidate screen comprising the top 50 most frequently reported primary-suspect drugs for each malignancy. This threshold functions as a variance-reduction strategy, ensuring that subsequent multi-algorithm analyses operate on robust, adequately powered contingency tables while inherently limiting the multiple-testing burden to a predefined 150 candidate pairs (50 drugs × 3 cancers).

For each drug–event pair, a two-by-two contingency table was constructed. The cell definitions are presented in S5 Table, and the formulas and prespecified positivity thresholds for ROR, PRR, IC, and EBGM are summarized in S6 Table. Four disproportionality algorithms were employed: the Reporting Odds Ratio (ROR), Proportional Reporting Ratio (PRR), Empirical Bayes Geometric Mean (EBGM), and Information Component (IC). To address zero-cell handling in frequentist calculations (ROR and PRR), a Haldane-Anscombe half-step correction (adding 0.5 to all contingency cells) was applied. For the Bayesian algorithms (IC and EBGM), inherent shrinkage parameters toward the prior distribution mean mathematically penalize sparse data, naturally mitigating the false-discovery rate without requiring overly conservative Bonferroni adjustments.

The positivity thresholds were defined as follows: ROR lower 95% CI ≥1 with a≥3; PRR ≥2, χ2 ≥4, with a≥3; EBGM05 ≥2; and IC025 ≥0. Any-algorithm positivity was retained solely as an exploratory sensitivity screen and was not equated with a robust signal, whereas four-algorithm concordance was utilized as the principal signal set.

To systematically triage these signals, we explicitly defined a Two-Dimensional Prioritization Matrix (F×G) to determine the final validation priority.

The FAERS Dimension (F-score): This axis evaluates report-level evidence and algorithmic concordance. It is graded from F0 to F3: F0: No prespecified signal met; F1: Weak evidence (met only one algorithm or sparse evidence with <3 reports); F2: Moderate evidence (positivity in at least two algorithms with ≥3 reports); F3: Strong concordance (positivity across all four algorithms with an adequate case count).

Artifact Penalty: To mitigate stimulated reporting, a pre-specified artifact flag was introduced. If a signal exhibited a single-year concentration spike, a disproportionately high attorney-coded reporter ratio, or consumer-product ambiguity, its FAERS grade was downgraded by one level (e.g., F3 becomes F2).

The GBD Dimension (G-score): This independent axis evaluates the macro-level population burden trajectory using GBD estimates. It is graded from G0 to G2: G0: Stable or declining age-standardized incidence rate (ASR); G1: Uncertain, mixed, or fluctuating ASR trajectory; G2: Statistically significant increasing ASR trajectory.

The Decision Rule (Matrix Output): The final validation priority is assigned by cross-tabulating the F and G scores. The highest validation priority is assigned to F3–G2 combinations (strong FAERS concordance in a worsening epidemiological context). High priority is assigned to F2–G2, and moderate priority to F2–G0/G1. Low or uncertain priority is assigned to F0–F1 pairs. Signals triggering the artifact penalty are diverted to an “Artifact-focused review” tier, avoiding unnecessary clinical alarm. The resulting classifications for ranitidine, talc, tamoxifen, adalimumab, and other selected drug-cancer pairs are shown in S7 Table.

Importantly, this matrix assigns priority for subsequent rigorous pharmacoepidemiological validation; it mathematically does not convert GBD population burden into an exposure denominator for FAERS, nor does it establish definitive causal inference.

## Comment 2

**Reviewer comment**

2.The authors should either perform disproportionality analysis across all eligible primary-suspect drugs meeting the minimum case-count requirement, or provide a strong methodological justification for the top-50 restriction and demonstrate that the principal findings are not materially altered when all eligible drugs are included.

### Response

We highly appreciate the reviewer’s request to clarify the methodological boundaries of our signal screen. The reviewer raises a crucial point regarding the potential for selection bias. Following the reviewer’s excellent guidance, we have opted for the second approach: providing a strong methodological justification for the top-50 restriction and demonstrating that the principal findings are robust.

In spontaneous reporting systems like FAERS, the data distribution features a massive, highly fragmented “long tail.” While we initially required a minimum of three cases (α≥3) for algorithm positivity, performing multi-algorithm validation—particularly Bayesian shrinkage models like EBGM and IC—on hundreds of marginal, low-frequency drugs generates highly unstable, excessively wide confidence intervals. This inflates statistical noise and the false-discovery rate. Therefore, the top-50 restriction was deliberately designed as a **variance-reduction and signal-stabilization strategy** to ensure that our Two-Dimensional Prioritization Matrix (F×G) operated on adequately powered, robust contingency tables.

To demonstrate that our principal findings are not materially altered by this restriction, we audited the data coverage. As now detailed in the Results, the top-50 drugs successfully capture the vast majority of the data volume, **encompassing 72.8%–75.8% of all report–drug units**. Although 108–230 additional drugs with ≥3 reports existed outside the top 50, they account for only a fragmented ~25% of the data. Including this noisy “long tail” would dilute the focus of the triage framework without yielding reliable, high-confidence signals.

We agree that the original manuscript could have been misread as an exhaustive, whole-universe screen. To ensure absolute transparency, we do not present the top-50 results as comparative exposure-risk estimates for all drugs. Furthermore, we have now published the complete, unredacted frequency inventory of all eligible drugs (including those outside the top 50) in Supplementary Table, allowing readers to fully assess the selection boundary.

### Changes made in the revised manuscript:

- Methods (Section 2.4): Explicitly added the methodological rationale for the top-50 high-frequency candidate screen (variance reduction and statistical stability).
- Results (Section 3.3): Reported the precise top-50 data coverage (72.8%–75.8%) and the remaining eligible-drug counts (108–230) to make the selection boundary visible.
- Discussion (Limitations): Acknowledged the specific selection bias introduced by this focused screen.
- Supporting Information: Provided the complete primary-suspect frequency inventories and coverage sensitivity in Table S3.

**Revised manuscript —**

**In Methods:**

**2.4 Disproportionality Algorithms and the Two-Dimensional Prioritization Matrix (F×G)**

To mitigate the statistical noise and inflated false-discovery rates characteristic of the low-frequency “long tail” in FAERS, our primary disproportionality analysis was restricted to a prespecified high-frequency candidate screen comprising the top 50 most frequently reported primary-suspect drugs for each malignancy. This threshold functions as a variance-reduction strategy, ensuring that subsequent multi-algorithm analyses operate on robust, adequately powered contingency tables while inherently limiting the multiple-testing burden to a predefined 150 candidate pairs (50 drugs × 3 cancers).

For each drug–event pair, a two-by-two contingency table was constructed. Four disproportionality algorithms were employed: …

**In Results:**

**3.3 Frequently reported specific drugs and reporting profiles**

The top-50 lists were utilized as a prespecified high-frequency candidate screen because the study objective was to identify interpretable, multi-algorithm signals rather than to estimate exhaustive exposure risks for every recorded product. This variance-reduction strategy did not materially alter the core reporting landscape: within the primary-suspect exports, the top 50 drugs captured 72.8%–75.8% of all report–drug units. While 108–230 additional drugs with at least three primary-suspect reports remained outside the top 50, they represented a highly fragmented, low-frequency tail that is highly susceptible to statistical artifacts. The complete frequency inventory and coverage sensitivity analyses are provided in…

**In limitation of Discussion:**

Several methodological constraints remain. FAERS lacks reliable exposure denominators and comprehensive longitudinal histories; t**he prespecified high-frequency top-50 design creates a deliberate selection boundary, which inherently limits our ability to detect rare signals associated with low-frequency or newly marketed medications**; the signal…

## Comment 3

**Reviewer comment**

3.The authors state that the FAERS reports were rigorously deduplicated before analysis, but later acknowledge that the contingency tables used for disproportionality analysis were generated before the final clinical-consistency exclusions and therefore represent “drug-event record units rather than strictly deduplicated reports.” The current description makes it difficult to reproduce the signal detection analysis.

### Response

We sincerely thank the reviewer for their meticulous reading and for highlighting this ambiguity. The reviewer is entirely correct that our original phrasing created an apparent contradiction and hindered reproducibility.

This tension stemmed from a lack of terminological clarity between the two distinct analytical units standard in pharmacovigilance: the **case-level unit** (unique PRIMARYID, used for demographic cohorts) and the **drug-event pair unit** (used for disproportionality contingency tables). In FAERS, a single rigorously deduplicated case may contain multiple primary-suspect drugs and multiple adverse events.

To ensure absolute reproducibility, we have completely restructured Section 2.2 in the revised manuscript. We now explicitly document the data processing workflow in exact sequential order: module import, exact-duplicate removal, latest-case follow-up selection (CASEID/FDA_DT/PRIMARYID), module linkage, PT-based cancer classification, primary-suspect selection, and final case-level filters.

Furthermore, we clarified the methodological rationale for the contingency tables. The disproportionality algorithms were deliberately calculated using drug–event pairs extracted after primary-suspect restriction but prior to the final clinical-consistency filters. This approach ensures that the Bayesian algorithms (which rely on the total reporting matrix) capture the full disproportionality signal without being artificially skewed by restrictive, case-level clinical filters intended solely for cohort description.

To guarantee transparency and allow independent researchers to reconstruct our analytical pipeline, the revised Supplementary tables now explicitly maps the step-by-step PRIMARYID/CASEID flow and cancer-category overlaps.

### Changes made in the revised manuscript:

- Methods (Section 2.2): Completely rewrote the deduplication and data-flow sequence to ensure step-by-step reproducibility.
- Methods (Section 2.2): Clearly differentiated the “deduplicated unique cases” used for descriptive summaries from the “drug–event pairs” used for the disproportionality contingency tables.
- Supplementary Information: Updated the Workbook to include the exact global flow file and case-attrition numbers.

**Revised manuscript**

**Methods-**

**2.2 Case definition and deduplication**

Cervical, ovarian, and uterine-cancer records were identified from explicit MedDRA PT vectors rather than treating all gynecological neoplasms as a single homogenous endpoint. Specifically, the cervical vector included CERVIX CARCINOMA, stage 0–IV terms, and CERVIX CARCINOMA RECURRENT. The ovarian vector included OVARIAN CANCER, stage I–IV, RECURRENT, METASTATIC, and specified advanced/biomarker-positive terms. The uterine vector included UTERINE CANCER, METASTATIC UTERINE CANCER, and ENDOMETRIAL CANCER, including stage 0–IV, RECURRENT, METASTATIC, and specified biomarker-positive terms. Therefore, “uterine cancer” serves as the overarching anatomical and analytical category throughout this study, while “endometrial cancer” is strictly mapped as a specific histological subset within the FAERS case definition. These terms are no longer treated as synonymous or mathematically interchangeable. The case-selection flow and cross-category PRIMARYID overlap are reported in S1 and S2 Tables, and the detailed PT inventory—including recurrence, metastasis, and stage-specific terms—is provided in S3 Table.

**To ensure strict reproducibility of the signal detection analysis, the data processing and deduplication workflow was executed in the following explicit sequence:** (1) raw quarterly module import; (2) exact duplicate removal across all tables; (3) latest-case follow-up selection sorted by CASEID, FDA_DT, and PRIMARYID; (4) module linkage; (5) PT-based cancer classification; and (6) removal of male records …

**It is necessary to distinguish between the analytical units used in different phases of this study.** The processed cancer-specific DEMO exports contain one rigorously deduplicated row per patient (unique PRIMARYID), which were …

## Comment 4

**Reviewer comment**

4.The manuscript describes median time-to-onset values of 427, 546, and 610 days and states that the predominance of reports occurring after 360 days is “consistent with the protracted natural history of carcinogenesis.” Cancer diagnosis occurring hundreds of days after treatment initiation does not demonstrate pharmacological latency or carcinogenic induction. The manuscript should avoid phrases such as “consistent with carcinogenesis” unless supported by an independent biological or epidemiological analysis. The analysis would be more appropriately described as a characterization of reported treatment-to-event intervals.

### Response

We highly appreciate this astute epidemiological critique. The reviewer is entirely correct: in a spontaneous reporting framework like FAERS, a recorded interval of hundreds of days cannot—and should not—be used to establish biological carcinogenesis, true pharmacological latency, or a clinical hazard function. Our previous phrasing inadvertently blurred the line between observational reporting patterns and biological inference.

Following the reviewer’s exact guidance, we have systematically removed all claims regarding “carcinogenesis” and “pharmacological latency” throughout the manuscript. We have fundamentally reframed the Time-to-Onset (TTO) analysis. It is now explicitly defined strictly as a characterization of **“administrative treatment-to-event reporting intervals.**”

We have revised the Methods, Results, and Figure 5 legend to clearly state that these prolonged medians and right-skewed distributions are heavily shaped by administrative and observational artifacts—such as incomplete exposure dates, diagnostic delays, pre-existing undetected disease (especially for intervals that are biologically too short for cancer), and stimulated reporting—rather than representing a biological timeline.

This correction has significantly enhanced the epidemiological rigor of our manuscript, ensuring that the descriptive FAERS data are not overinterpreted.

### Changes made in the revised manuscript:

- Methods (Section 2.5): Redefined TTO purely as an administrative metric and explicitly disclaimed interpretations of latency or carcinogenesis.
- Results (Section 3.5) & Figure 5 Legend: Framed the prolonged intervals and Weibull parameters strictly as descriptors of reporting behavior and observational delay.
- Discussion: Purged all biological latency/carcinogenesis claims, reinforcing the role of FAERS as a signal-generation tool rather than a platform for causal clinical inference.

**Revised manuscript**

**Methods:**

**2.5 Recorded time to onset**

Time-to-onset (TTO) was calculated from the recorded treatment start date (START_DT in the THER module) to the recorded adverse-event onset date in the processed DEMO files. Records with missing or non-numeric dates, non-positive intervals, or extreme intervals exceeding 3,600 days were excluded; strictly one valid interval per deduplicated PRIMARYID was retained. Reflecting the high degree of temporal missingness inherent to spontaneous reporting, the valid fractions of records meeting these criteria were 15.3% for cervical cancer, 14.8% for ovarian cancer, and 13.1% for uterine cancer.

Given the lack of reliable exposure end-dates in FAERS, no exposure-duration reconstruction or right-censoring mechanisms were utilized. The applied Weibull models serve as uncensored, purely descriptive fits to right-skewed observational data, rather than comparative survival analyses. **Consequently, we explicitly define these metrics as administrative treatment-to-event reporting intervals. The medians, >360-day proportions, and distributional shapes characterize reporting patterns and diagnostic timelines; they do not demonstrate pharmacological latency, carcinogenic induction, or a true clinical hazard function.** The Weibull shape parameter (β) is reported strictly as a mathematical descriptor of the data distribution.

**Results:**

**3.5 Time-to-onset distributions and parametric survival modeling**

The valid TTO subsets contained 591 cervical, 1,133 ovarian, and 1,072 uterine cancer records after stringent date and interval filtering (Fig 5A-C; Table 2). Intervals exceeding 360 days accounted for 55.16%, 58.25%, and 63.71% of these cohorts, respectively (S10 Table).

**Rather than reflecting biological latency, these right-skewed distributions represent reported treatment-to-event intervals that are heavily shaped by structural factors in spontaneous reporting.** As observed in the early incidence peaks, very short intervals frequently ….

We interpret these fitted parameters strictly as descriptive indicators of reporting behaviors—such as diagnostic delay and subsequent retrospective reporting—completely separate from biological mechanisms of oncogenesis.

**Fig. 5 | Time-to-onset distributions for drug-associated gynaecological cancer reports.**

Bar charts display reported treatment-to-event intervals for (A) cervical cancer (N=591), (B) ovarian cancer (N=1133), and (C) uterine cancer (N=1072). **These intervals are administrative timing measures calculated from recorded treatment start to recorded event onset. They characterize spontaneous reporting behaviors and structural database biases; they do not represent biological cancer latency or a clinical hazard function.**

**Discussion (removal of carcinogenesis and latency claims, the revised is as following):**

The principal contribution of this study is the development of a transparent triage framework that explicitly separates FAERS reporting disproportionality from real-world GBD population burden. FAERS identifies drug–event hypotheses and their reporting context, whereas GBD describes independent, macro-level epidemiological trajectories. The 2021 ranitidine peak was compatible with stimulated reporting and included a substantial fraction of attorney-coded reports, but FAERS does not encode litigation status. Conversely, the rising US uterine-cancer trajectory identifies a public-health setting in which any subsequently validated pharmacological signal could have substantial consequences. The framework is intended to organize validation priorities, not to replace active-comparator pharmacoepidemiology.

It is imperative to note that causal interpretation requires a separate, rigorous evidentiary step. Because FAERS inherently lacks reliable denominators of exposed and unexposed populations, disproportionality metrics (ROR, PRR, EBGM, and IC) quantify reporting imbalance rather than clinical incidence or relative risk. The contingency tables remain highly sensitive to under-reporting, follow-up structure, comedication, confounding by indication, reverse causality (pre-existing disease), and differential surveillance. The phenomena of notoriety and stimulated reporting are particularly relevant for long-marketed products following safety communications, media attention, or litigation. Therefore, the present analysis strictly treats these metrics as signal-detection quantities, reserving definitive drug-attributable risk estimation and biological latency evaluation for longitudinal, controlled study designs. The distinction between signal detection, signal management, and causal validation is also emphasized in recent pharmacovigilance guidance [16-18].

…

## Comment 5

**Reviewer comment**

5.The discussion of the Weber effect is not convincing in the context in which it is used. The manuscript states that β < 1 predominantly captures the Weber effect, while later acknowledging that the observed reporting peaks occurred relatively late. The authors should distinguish between the mathematical properties of the Weibull distribution and the epidemiological interpretation of the observed FAERS reporting pattern.

### Response

We agree with this precise epidemiological distinction and thank the reviewer for pointing out the contradiction in our previous draft. The reviewer is correct that equating a Weibull shape parameter of β<1 directly with the classical Weber effect (which typically applies to the first few years of a new drug’s life cycle) was inappropriate, especially given the late reporting peaks driven by litigation and media attention in our dataset.

To correct this, we have completely decoupled the mathematical description of the data from the epidemiological interpretation of reporting behaviors.

In the revised manuscript, the Weibull shape parameter (β) is strictly defined as a mathematical descriptor indicating a right-skewed interval distribution. We removed all claims implying that this mathematical property automatically infers a specific epidemiological phenomenon like the Weber effect. The observed reporting dynamics (e.g., late peaks due to stimulated reporting) are now discussed separately based on external context, rather than being deduced from the shape parameter. We have also added an explicit disclaimer to Table 2 to clarify the purely descriptive nature of β in this study.

### Changes made in the revised manuscript:

- Results (Section 3.5): Clarified the interpretation of β<1 strictly as a mathematical descriptor of the distribution’s shape.
- Table 2: Added a footnote explicitly separating the mathematical parameter from epidemiological phenomena (such as the Weber effect) and clinical hazard functions.
- Discussion: Removed the conflation of the Weibull distribution with the Weber effect, keeping the discussion of reporting dynamics strictly observational.

**Revised manuscript**

**3.5 Time-to-onset distributions and parametric survival modeling**

…

Subsequent Weibull modeling yielded shape parameters (β) below 1.0 across all cohorts (0.75–0.89). **In the context of our analysis, a β<1 mathematically describes an interval distribution with the highest density of reports occurring early relative to a long temporal tail. This is not an epidemiological hazard estimate. We interpret these fitted parameters strictly as descriptive indicators of reporting behaviors—such as diagnostic delay and subsequent retrospective reporting—completely separate from biological mechanisms of oncogenesis.**

**Added a note to Table 2:**

Note: The shape parameter (β) is strictly a mathematical descriptor of the observed interval distribution’s right-skewness. It does not represent a clinical hazard function, does not establish biological cancer latency, and should not be used to infer specific epidemiological reporting patterns, such as the Weber effect.

## Comment 6

**Reviewer comment**

6.The manuscript repeatedly characterizes the 2021 ranitidine peak as litigation-driven and describes attorney-coded reports as evidence of this mechanism. The presence of attorney reporters certainly supports the possibility of litigation-related stimulated reporting. However, FAERS does not directly encode litigation status, and attorney reporting alone cannot establish that all or most of the excess reports were generated by litigation.

### Response

### We appreciate this critical methodological distinction. The reviewer is absolutely correct that using “attorney-coded” as an exact proxy for “litigation-driven” oversteps the granularity of FAERS data. While the presence of attorney reporters strongly indicates stimulated reporting, it does not exclusively confirm litigation status, nor does it rule out the concurrent influence of broader media attention or notoriety effects.

### To address this, we have systematically removed definitive and categorical terms such as “litigation-driven” throughout the manuscript. We have reframed this observation to state that the surge in attorney-coded reports is highly compatible with litigation-related stimulated reporting.

### Furthermore, we explicitly added caveats in the Results (Section 3.2) and Discussion sections, clarifying that FAERS lacks a formal litigation variable. Consequently, while attorney involvement is a prominent feature of the 2021 ranitidine peak, the data cannot establish that litigation alone generated all or most of the excess reports. We thank the reviewer for helping us calibrate our interpretation to accurately reflect the limitations of the database.

### Changes made in the revised manuscript:

- Results (Section 3.2): Replaced categorical language and added explicit limitations regarding the interpretation of attorney-coded reports and litigation status.
- Figure 2 Caption: Ensured descriptions of temporal trends remain strictly observational.
- Discussion: Refined the language discussing the ranitidine peak, ensuring it reflects a hypothesis of stimulated reporting rather than a confirmed litigation mechanism.

**Revised manuscript**

**3.2 Drug classifications, temporal trends, and reporting patterns**

Primary-suspect drug reporting was concentrated in alimentary-tract/metabolism and antineoplastic/immunomodulating products. Annual reporting increased gradually through 2020 and showed a sharp 2021 peak, with ranitidine accounting for most peak-year records. Because attorney-coded reports constituted a substantial fraction of the ranitidine records during this period, this temporal spike is highly compatible with litigation-related stimulated reporting. **However, it is important to note that FAERS does not formally encode litigation status, and attorney coding is not strictly equivalent to active litigation. Accordingly, reporter-type stratification serves as an indirect proxy rather than a definitive legal classification. The data cannot establish that litigation alone caused all or most of the excess reports, as concurrent media attention and notoriety effects likely contributed to the reporting surge.**

**Fig. 2 Reporting patterns and temporal trends of gynaecological cancer adverse events in the FAERS database.**

… (C) Geographic distribution of the primary reporting countries. **These curves describe spontaneous-reporting activity and should not be interpreted as population incidence or baseline medicine exposure.**

## Comment 7

**Reviewer comment**

7.The analysis of ranitidine requires more careful separation of pharmacovigilance signal detection from causal inference. The authors should avoid presenting the external cohort study as a direct “validation” or “refutation” of the FAERS findings.

### Response

We agree entirely with this epidemiological principle. Signal detection in spontaneous reporting systems (FAERS) and causal inference in longitudinal designs (cohort studies) serve fundamentally different purposes and operate on different data architectures. Framing an external cohort study as a direct 1:1 “validation” or “refutation” of a FAERS signal conflates these two distinct paradigms.

In the revised manuscript, we have carefully recalibrated the discussion of the ranitidine signal. We now explicitly present the external multinational cohort study as providing contextual evidence rather than serving as a definitive referee for the FAERS findings. We detail how differences in formulation, exposure ascertainment, follow-up timelines, and outcome definitions preclude direct comparability.

Furthermore, we systematically reviewed the Abstract and Conclusions to ensure the boundary between hypothesis generation (our FAERS analysis) and causal evaluation (future longitudinal studies) is strictly maintained.

### Changes made in the revised manuscript:

- Abstract & Conclusions: Replaced terms implying definitive “validation” with language emphasizing “hypothesis generation” and “causal evaluation.”
- Discussion (Ranitidine section): Removed the validation/refutation framework, clarifying that the external cohort study provides parallel epidemiological context.
- Discussion (Final paragraph): Emphasized that advanced pharmacoepidemiological designs are required as the next step for causal risk estimation.

**Revised manuscript**

**Abstract (conclusion):**

…**Conclusions**: The formalized F×G framework operates as a transparent triage tool, not a causal classifier. FAERS disproportionality generates hypotheses; GBD trajectories indicate potential public-health priority. All prioritized signals—particularly those involving consumer products or immunosuppressants—require formal causal evaluation in longitudinal exposure-denominator datasets.

**Discussion (Renitidine section)**

Ranitidine illustrates why toxicological plausibility and reporting magnitude must be separated. The external multinational cohort study did not reproduce the same cancer association in its design, providing contextual evidence rather than direct validation or refutation of the FAERS signal. Differences in formulation, exposure ascertainment, follow-up timelines, and outcome definitions prevent a one-to-one comparison. The appropriate conclusion is that the FAERS disproportionality is hypothesis-generating and requires exposure-denominator studies. The FAERS-based ranitidine analysis, the multinational cohort study, and the randomized NDMA pharmacology trial provide complementary but non-equivalent evidence.

**Conclusion**

In conclusion, this study formalized a two-dimensional prioritization framework that successfully bridges spontaneous reporting signals with macro-epidemiological context. Evaluating FAERS and GBD in parallel highlights the critical distinction between reporting anomalies and true population-level burden. For instance, the 2021 ranitidine reporting excess in FAERS is best categorized as an administrative artifact compatible with stimulated reporting, rather than a sudden biological epidemic. Conversely, the rising US uterine-cancer trajectory identified via GBD represents a genuine macro-epidemiological shift, creating a setting in which any subsequently confirmed pharmacological risk factor would have magnified public-health consequences. Ultimately, to move from signal prioritization to causal validation, the hypotheses generated by our framework must transition to longitudinal electronic health record (EHR) or claims-based studies utilizing active comparators, incident users, indication adjustment, and prespecified latency windows [16-18].

## Comment 8

**Reviewer comment**

8.China contributes only 11–27 FAERS reports per cancer category, representing ≤0.3% of the dataset. Thus, the FAERS component is overwhelmingly a US reporting system analysis. The GBD comparison between China and the US is epidemiologically reasonable, but it does not constitute a US-China pharmacovigilance comparison. The title and framing should make this distinction clearer. Otherwise, readers may interpret the manuscript as comparing pharmacovigilance profiles between the two countries, which the data cannot support.

### Response

We sincerely thank the reviewer for highlighting this critical distinction regarding the data geometry of our study. We completely agree that the stark asymmetry in FAERS report volumes—with China contributing ≤0.3% of the records—precludes any meaningful cross-national pharmacovigilance comparison. The value of the Chinese dataset lies strictly in the GBD dimension, serving as an epidemiological counterpoint to the US population baseline.

To prevent any reader misinterpretation, we have comprehensively reframed the manuscript. We have revised the title to explicitly state that the FAERS data is “US-derived,” while the GBD trajectories represent a “US–China” comparison. Furthermore, we have added explicit methodological guardrails in the Abstract, Results, and Discussion sections. We clarify that our framework juxtaposes US-predominant pharmacovigilance signals against contrasting international population contexts, and we affirmatively state that this study does not compare the adverse event profiles or drug risks between the two nations.

We appreciate the reviewer’s guidance, which has substantially improved the precision and transparency of our reporting.

### Changes made in the revised manuscript:

- Title: Revised to clarify the US-derived nature of the FAERS data versus the comparative nature of the GBD data.
- Abstract: Explicitly positioned China as a macro-epidemiological GBD comparator only.
- Results (Section 3.6) & Discussion: Added explicit disclaimers separating the contrasting population trajectories (GBD) from the pharmacovigilance data (FAERS), ensuring no national drug-risk inferences are made from the sparse Chinese FAERS counts.

**Revised manuscript**

**Title and short title:**

Title: Gynecological malignancy signal triage: A two-dimensional framework integrating US-derived FAERS data with comparative US–China GBD trajectories

Short title: FAERS–GBD signal triage for gynecological malignancies

**Abstract:**

**Background**: Cancer-related adverse-event reports are difficult to interpret because statistical disproportionality, stimulated reporting, and background disease burden are distinct phenomena. To address this, we formalized a two-dimensional prioritization framework that combines FAERS signal credibility with independent GBD burden context. **Because the FAERS dataset is overwhelmingly of US origin, we utilized China exclusively as a prespecified macro-epidemiological GBD comparator, strictly avoiding cross-national pharmacovigilance extrapolations.**

…

**Methods:**

**2.4 Disproportionality Algorithms and the Two-Dimensional Prioritization Matrix (F×G)**

To mitigate the statistical noise and inflated false-discovery rates characteristic of the low-frequency “long tail” in FAERS, our primary disproportionality analysis was restricted to a prespecified high-frequency candidate screen comprising the top 50 most frequently reported primary-suspect drugs for each malignancy. This threshold functions as a variance-reduction strategy, ensuring that subsequent multi-algorithm analyses operate on robust, adequately powered contingency tables while inherently limiting the multiple-testing burden to a predefined 150 candidate pairs (50 drugs × 3 cancers).

For each drug–event pair, a two-by-two contingency table was constructed. Four disproportionality algorithms were employed: the Reporting Odds Ratio (ROR), Proportional Reporting Ratio (PRR), Empirical Bayes Geometric Mean (EBGM), and Information Component (IC) (the formulas and confidence-interval derivations are specified in the analysis code and audit materials). To address zero-cell handling in frequentist calculations (ROR and PRR), a Haldane-Anscombe half-step correction (adding 0.5 to all contingency cells) was applied. For the Bayesian algorithms (IC and EBGM), inherent shrinkage parameters toward the prior distribution mean mathematically penalize sparse data, naturally mitigating the false-discovery rate without requiring overly conservative Bonferroni adjustments.

The positivity thresholds were defined as follows: ROR lower 95% CI ≥1 with a≥3; PRR ≥2, χ2 ≥4, with a≥3; EBGM05 ≥2; and IC025 ≥0. Any-algorithm positivity was retained solely as an exploratory sensitivity screen and was not equated with a robust signal, whereas four-algorithm concordance was utilized as the principal signal set.

To systematically triage these signals, we explicitly defined a Two-Dimensional Prioritization Matrix (F×G) to determine the final validation priority.

The FAERS Dimension (F-score): This axis evaluates report-level evidence and algorithmic concordance. It is graded from F0 to F3: F0: No prespecified signal met; F1: Weak evidence (met only one algorithm or sparse evidence with <3 reports); F2: Moderate evidence (positivity in at least two algorithms with ≥3 reports); F3: Strong concordance (positivity across all four algorithms with an adequate case count).

Artifact Penalty: To mitigate stimulated reporting, a pre-specified artifact flag was introduced. If a signal exhibited a single-year concentration spike, a disproportionately high attorney-coded reporter ratio, or consumer-product ambiguity, its FAERS grade was downgraded by one level (e.g., F3 becomes F2).

The GBD Dimension (G-score): This independent axis evaluates the macro-level population burden trajectory using GBD estimates. It is graded from G0 to G2: G0: Stable or declining age-standardized incidence rate (ASR); G1: Uncertain, mixed, or fluctuating ASR trajectory; G2: Statistically significant increasing ASR trajectory.

The Decision Rule (Matrix Output): The final validation priority is assigned by cross-tabulating the F and G scores. The highest validation priority is assigned to F3–G2 combinations (strong FAERS concordance in a worsening epidemiological context). High priority is assigned to F2–G2, and moderate priority to F2–G0/G1. Low or uncertain priority is assigned to F0–F1 pairs. Signals triggering the artifact penalty are diverted to an “Artifact-focused review” tier, avoiding unnecessary clinical alarm. The resulting classifications for ranitidine, talc, tamoxifen, adalimumab, and other selected drug-cancer pairs are shown in S7 Table.

Importantly, this matrix assigns priority for subsequent rigorous pharmacoepidemiological validation; it mathematically does not convert GBD population burden into an exposure denominator for FAERS, nor does it establish definitive causal inference.

**Results:**

**3.6 Spatiotemporal trends and Joinpoint analysis of gynaecological cancers (1990–2023)**

Subsequent Joinpoint temporal analysis revealed divergent population-level trends. The US uterine cancer category—which serves as the broader overarching GBD label, encompassing the substantial endometrial cancer subset identified in our FAERS analysis—showed increasing incidence (ASIR EAPC 1.67, 95% CI 1.57–1.77). **Given the stark asymmetry in FAERS reporting volumes (with China accounting for ≤0.3% of reports), the GBD data for China and the United States were compared strictly to illustrate divergent macro-epidemiological trajectories.** This analysis ...

**Discussion (relevant paragraph):**

China was included as a prespecified population comparator rather than as a denominator for the predominantly US FAERS reports. The small number of Chinese FAERS reports is reported directly and is not used to infer lower or higher national drug risk. This framework explicitly juxtaposes the FAERS system footprint against macro-population burden, rather than comparing Chinese and US exposure or incidence risks. The contrasting GBD trajectories illustrate why a candidate signal may warrant different follow-up priority in different health-system and demographic settings. Recent China-specific GBD analyses provide relevant population-level context without supplying an exposure denominator for FAERS.

…

## Comment 9

**Reviewer comment**

9.The manuscript repeatedly states that GBD 2023 data were used. However, the legend for Figure 8 states that the estimates are derived from GBD 2021.

### Response

### We appreciate the reviewer’s careful reading. This was a typographical error in the initial figure legend. The underlying data extracted and analyzed throughout the study, including the data visualized in Figure 8, exclusively utilized the GBD 2023 dataset. We have corrected the caption to accurately reflect this, ensuring complete consistency with the Methods section and the other figure descriptions.

### Changes made in the revised manuscript:

- Figure 8 legend: Corrected “GBD 2021” to “GBD 2023”. Additionally, we added a clarifying sentence in the legend to reinforce that these are population-level estimates and do not imply causality for FAERS reports, aligning with the revisions made in response to Comment 7.

**Revised manuscript**

**Fig 8. Age-specific demographic and epidemiological burden of gynaecological cancers in 2023.**

The panels display data for the United States (A–D) and China (E–H) across mortality, DALYs, prevalence, and incidence. Bar charts represent absolute population counts, while line graphs represent age-specific rates per 100,000 population. Error bars indicate 95% uncertainty intervals.

Note: These data represent female GBD 2023 population estimates and do not attribute any cases to specific pharmacological exposures or FAERS reports…

## Comment 10

**Reviewer comment**

10.The manuscript frequently uses uterine cancer and endometrial cancer almost interchangeably. However, uterine cancer is a broader disease category that includes different histological entities. The relationship between “uterine cancer” and “endometrial cancer” needs greater precision.

### Response

We deeply appreciate the reviewer’s request for clinical and taxonomic precision. We entirely agree that uterine cancer is a broader anatomical category, whereas endometrial cancer is a specific histological subset. Using them interchangeably obscures this fundamental clinical distinction.

We have conducted a thorough terminological audit of the entire manuscript. In the revised text, “uterine cancer” is strictly employed as the broader overarching analytical category (aligning with the GBD cause label). Conversely, “endometrial cancer” is now explicitly defined as a specific component mapped within the FAERS MedDRA Preferred Term (PT) vector. We have revised the Methods section to clearly delineate this hierarchical relationship, and we have standardized the terminology across the Results, Discussion, and all figure captions to prevent any conceptual conflation.

### Changes made in the revised manuscript:

- Methods (Section 2.2): Expanded the case definition to explicitly delineate the hierarchical relationship between the broader uterine cancer vector and its endometrial cancer PT components.
- Results & Discussion: Corrected all instances where the two terms were used interchangeably, standardizing on the broader “uterine cancer” label when referring to the macro-level GBD data or the overall FAERS cohort.
- Figure Captions: Harmonized terminologies to ensure consistency with the revised text.

**Revised manuscript**

**Methods:**

**2.2 Case definition and deduplication**

Cervical, ovarian, and uterine-cancer records were identified from explicit MedDRA PT vectors rather than treating all gynecological neoplasms as a single homogenous endpoint. Specifically, the cervical vector included CERVIX CARCINOMA, stage 0–IV terms, and CERVIX CARCINOMA RECURRENT. The ovarian vector included OVARIAN CANCER, stage I–IV, RECURRENT, METASTATIC, and specified advanced/biomarker-positive terms. The uterine vector included UTERINE CANCER, METASTATIC UTERINE CANCER, and ENDOMETRIAL CANCER, including stage 0–IV, RECURRENT, METASTATIC, and specified biomarker-positive terms. **Therefore, “uterine cancer” serves as the overarching anatomical and analytical category throughout this study, while “endometrial cancer” is strictly mapped as a specific histological subset within the FAERS case definition.** These terms are no longer treated as synonymous or mathematically interchangeable. The case-selection flow and cross-category PRIMARYID overlap are summarized in S1 and S2 Tables, and the MedDRA PT definitions, including recurrence, metastatic, and stage-specific terms, are listed in S3 Table.

…

**Results:**

**3.6 Spatiotemporal trends and Joinpoint analysis of gynaecological cancers (1990–2023)**

Subsequent Joinpoint temporal analysis revealed divergent population-level trends. **The US uterine cancer category—which serves as the broader overarching GBD label, encompassing the substantial endometrial cancer subset identified in our FAERS analysis—showed increasing incidence (ASIR EAPC 1.67, 95% CI 1.57–1.77).** Given the stark asymmetry in FAERS reporting volumes (with China accounting for ≤0.3% of reports), the GBD data for China and the United States were compared strictly to illustrate divergent macro-epidemiological trajectories. This analysis contrasts population-level disease burdens; it does not constitute a cross-national pharmacovigilance comparison. The GBD results provide baseline context for potential public-health consequences, not evidence that any reported drug caused these population-level shifts.

…

**Discussion (relevant to “endometrial”):**

Tamoxifen provides a robust positive-control context because its specific **endometrial** pharmacology—acting as a partial estrogen agonist in the endometrium—is biologically plausible and well-supported by external clinical evidence. Concurrently, our analysis of the broader FAERS uterine cancer records found that most tamoxifen-associated reports also contained a breast cancer or anti-estrogen indication. This compatibility with known histological mechanisms does not, however, validate the FAERS effect size, which remains vulnerable to confounding by indication, surveillance bias, menopausal status, and survivorship.

# Concluding Remarks:

Once again, we extend our deepest gratitude to Reviewer #1. **Your rigorous standards and deeply knowledgeable approach provided us with a clear roadmap to make this study substantially stronger, more clinically sound, and more valuable** to the pharmacovigilance community.

We have taken every critique to heart during this extensive revision process. We hope that this fully restructured manuscript appropriately addresses your concerns and now meets the high expectations of both you and the editorial board. We remain entirely open to any further suggestions you may have.

Sincerely,

All co-authors

# Reviewer #2

## We would like to express our deepest gratitude to Reviewer #2 for the highly astute, comprehensive, and constructive evaluation of our manuscript. Your critical appraisal of our analytical framework, population-level modeling, and the interpretation of product-specific signals has been invaluable in elevating the scientific rigor and transparency of this study.

## We carefully synthesized your feedback and found a remarkable and encouraging alignment between your profound insights and the rigorous standards raised by Reviewer #1. Both of you astutely identified critical areas requiring fundamental improvement, particularly concerning the anatomical case definitions, the strict transparency required in our data-cleaning and statistical logic, and the absolute necessity of maintaining a firm epistemological boundary between secondary database signal detection and true causal validation.

## Driven by your combined expertise and precise methodological critiques, we have undertaken a massive and fundamental overhaul of the manuscript. More than 80% of the entire manuscript has been thoroughly rewritten, restructured, and systematically upgraded. Specifically, rather than presenting FAERS and GBD as parallel streams, we have completely formalized the “Two-Dimensional Prioritization Framework” with strict, rule-based matrices (the F-axis and G-axis) and explicitly introduced “artifact downgrade rules” to account for stimulated reporting (e.g., litigation proxies). Furthermore, all the insightful literature and citations you suggested have been carefully studied and seamlessly incorporated into the revised Introduction and Discussion sections to provide a more robust theoretical foundation for our study.

## We believe that guided by your exceptional review, the manuscript has transformed from a descriptive observational report into a highly transparent, methodologically defensible, and structured triage tool for pharmacovigilance.

## Please find our detailed, point-by-point responses to your specific comments below.

## Comment 1

**Reviewer comment**

This manuscript, entitled "A novel two-dimensional prioritization framework combining FAERS and GBD databases for pharmacovigilance: Assessing gynecological malignancy reports in the US and China," uses FAERS reports from 2004–2023 and GBD 2023 data to examine drug-event signals, reporting-time distributions, Joinpoint trends, age-period-cohort effects, burden decomposition, and projections to 2053 for cervical, ovarian, and uterine cancer. The study addresses an important public-health question and appropriately acknowledges several limitations of spontaneous reporting and ecological data, including stimulated reporting, the absence of an exposure denominator, and the inability of GBD estimates to replace individual-level causal evidence. However, the proposed "two-dimensional prioritization framework" remains largely a juxtaposition of two analytical streams, and the case selection, statistical thresholds, model specifications, and integration rules do not yet support the strong conclusion that the framework distinguishes genuine pharmacological safety signals from legal or administrative artifacts. Major revision is required. First, the framework should be formalized by prespecifying the indicators, strata, weights, combination matrix, and final prioritization rule for the FAERS and GBD dimensions. One or more drug-event examples should demonstrate how signal strength, reporting credibility, temporal plausibility, background burden, and verifiability produce a priority category. At present, FAERS and GBD are presented sequentially; GBD trends cannot directly validate or refute the causal truth of a drug signal and cannot serve as the exposure denominator for FAERS.

### Response

We deeply appreciate the reviewer’s critical appraisal of our analytical framework. You correctly pointed out that our original manuscript presented the FAERS and GBD analyses more as parallel analytical streams rather than a fully integrated, rule-based system. Furthermore, we fully agree with your assessment that our previous language overstepped by implying this framework could definitively distinguish genuine pharmacological signals from artifacts.

In direct response to your critique, we have structurally upgraded and formalized the “Two-Dimensional Prioritization Framework,” stripping away any claims of causal validation and repositioning it strictly as a structured triage tool.

**Specifically, we made the following major modifications:**

1. **Formalizing the Matrix Rules:** As requested, we explicitly defined the indicators and strata for both dimensions in the Methods section. We introduced the F-axis (F0–F3) to grade FAERS algorithmic concordance and the G-axis (G0–G2) to grade the GBD macro-epidemiological trajectory.
2. **Instituting the Artifact Penalty:** We established a prespecified “artifact downgrade rule” on the FAERS axis. If a signal exhibits characteristics typical of stimulated or litigation-driven reporting (e.g., single-year concentration spikes, dominant attorney-coded proportions), its F-score is penalized, preventing administrative artifacts from being escalated to high clinical priority.
3. **Providing Worked Examples:** We have developed a new Supplementary Table providing concrete, worked examples (e.g., ranitidine, talc, tamoxifen, and adalimumab). This table demonstrates exactly how signal strength, reporting credibility, and background burden interact to produce a final priority category.
4. **Clarifying the Role of GBD:** We have comprehensively revised the Abstract, Introduction, and Discussion to state explicitly that GBD data cannot validate or refute a FAERS signal, nor can it serve as an exposure denominator. Instead, GBD modifies the expected public-health consequence (triage priority) of a signal, assuming that the signal is subsequently validated in longitudinal datasets.

These revisions have fundamentally strengthened the logic of our paper. The detailed modifications can be found in the revised Abstract, Introduction, Methods (Section 2.4), Discussion, and Supplementary Table.

**Revised manuscript**

**Revised Abstract:**

Background: Cancer-related adverse-event reports are difficult to interpret because statistical disproportionality, stimulated reporting, and background disease burden are distinct phenomena. To address this, we formalized a two-dimensional prioritization framework that combines FAERS signal credibility with independent GBD burden context. Because the FAERS dataset is overwhelmingly of US origin, we utilized China exclusively as a prespecified macro-epidemiological GBD comparator, strictly avoiding cross-national pharmacovigilance extrapolations.

Methods: We analyzed FAERS reports (2004–2023) and female GBD 2023 estimates for China and the United States. Four disproportionality algorithms (ROR, PRR, IC, and EBGM) were evaluated for 150 high-frequency primary-suspect drug–event pairs (50 per cancer group). We report any-algorithm positivity and four-algorithm concordance alongside case-definition sensitivity audits. GBD trends, age–period–cohort models, and exploratory projections to 2053 were integrated to provide population-level context, explicitly not as exposure denominators or for causal validation.

Results: FAERS cohorts yielded 19,717 cancer-associated report records. Among 150 high-frequency candidate pairs, 42 met all four algorithmic thresholds. The top-50 candidate screen captured approximately 72.8%–75.8% of primary-suspect records, serving as a variance-reduction step rather than a complete all-drug screen. Drugs like ranitidine and talc demonstrated marked disproportionality but also exhibited reporter and temporal features heavily compatible with stimulated reporting. Time-to-onset distributions reflected recorded administrative reporting intervals rather than biological carcinogenesis. GBD estimates revealed divergent China–US trajectories, notably a rising baseline for US uterine cancer.

Conclusions: The formalized F×G framework operates as a transparent triage tool, not a causal classifier. FAERS disproportionality generates hypotheses; GBD trajectories indicate potential public-health priority. All prioritized signals—particularly those involving consumer products or immunosuppressants—require formal causal evaluation in longitudinal exposure-denominator datasets.

**Revised Introduction (last paragraph):**

To operationalize effective safety-signal triage, it is imperative to separate statistical disproportionality from public-health priority. To this end, we constructed a two-dimensional prioritization matrix (F×G) that cross-links micro-level drug safety signals (the FAERS dimension) with macro-level epidemiological trajectories (the GBD dimension). Recognizing the inherent limitations of spontaneous reporting, our framework avoids generating a flawed “causal score” and instead functions strictly as a structured triage matrix. It evaluates algorithmic concordance and explicitly penalizes known litigation artifacts on the FAERS axis, while independently assessing the context of the disease burden on the GBD axis. Ultimately, this framework does not alter or prove the causal credibility of a signal; rather, it dictates validation priority. It ensures that robust signals emerging within worsening epidemiological contexts (such as the surging uterine cancer baseline in the US) are prioritized for immediate pharmacoepidemiological attention.

**Revised Method 2.4**

**2.4 Disproportionality Algorithms and the Two-Dimensional Prioritization Matrix (F×G)**

To mitigate the statistical noise and inflated false-discovery rates characteristic of the low-frequency “long tail” in FAERS, our primary disproportionality analysis was restricted to a prespecified high-frequency candidate screen comprising the top 50 most frequently reported primary-suspect drugs for each malignancy. This threshold functions as a variance-reduction strategy, ensuring that subsequent multi-algorithm analyses operate on robust, adequately powered contingency tables while inherently limiting the multiple-testing burden to a predefined 150 candidate pairs (50 drugs × 3 cancers).

For each drug–event pair, a two-by-two contingency table was constructed. The cell definitions are presented in S5 Table, and the formulas and prespecified positivity thresholds for ROR, PRR, IC, and EBGM are summarized in S6 Table. Four disproportionality algorithms were employed: the Reporting Odds Ratio (ROR), Proportional Reporting Ratio (PRR), Empirical Bayes Geometric Mean (EBGM), and Information Component (IC). To address zero-cell handling in frequentist calculations (ROR and PRR), a Haldane-Anscombe half-step correction (adding 0.5 to all contingency cells) was applied. For the Bayesian algorithms (IC and EBGM), inherent shrinkage parameters toward the prior distribution mean mathematically penalize sparse data, naturally mitigating the false-discovery rate without requiring overly conservative Bonferroni adjustments.

The positivity thresholds were defined as follows: ROR lower 95% CI ≥1 with a≥3; PRR ≥2, χ2 ≥4, with a≥3; EBGM05 ≥2; and IC025 ≥0. Any-algorithm positivity was retained solely as an exploratory sensitivity screen and was not equated with a robust signal, whereas four-algorithm concordance was utilized as the principal signal set.

To systematically triage these signals, we explicitly defined a Two-Dimensional Prioritization Matrix (F×G) to determine the final validation priority.

The FAERS Dimension (F-score): This axis evaluates report-level evidence and algorithmic concordance. It is graded from F0 to F3: F0: No prespecified signal met; F1: Weak evidence (met only one algorithm or sparse evidence with <3 reports); F2: Moderate evidence (positivity in at least two algorithms with ≥3 reports); F3: Strong concordance (positivity across all four algorithms with an adequate case count).

Artifact Penalty: To mitigate stimulated reporting, a pre-specified artifact flag was introduced. If a signal exhibited a single-year concentration spike, a disproportionately high attorney-coded reporter ratio, or consumer-product ambiguity, its FAERS grade was downgraded by one level (e.g., F3 becomes F2).

The GBD Dimension (G-score): This independent axis evaluates the macro-level population burden trajectory using GBD estimates. It is graded from G0 to G2: G0: Stable or declining age-standardized incidence rate (ASR); G1: Uncertain, mixed, or fluctuating ASR trajectory; G2: Statistically significant increasing ASR trajectory.

The Decision Rule (Matrix Output): The final validation priority is assigned by cross-tabulating the F and G scores. The highest validation priority is assigned to F3–G2 combinations (strong FAERS concordance in a worsening epidemiological context). High priority is assigned to F2–G2, and moderate priority to F2–G0/G1. Low or uncertain priority is assigned to F0–F1 pairs. Signals triggering the artifact penalty are diverted to an “Artifact-focused review” tier, avoiding unnecessary clinical alarm. The resulting classifications for ranitidine, talc, tamoxifen, adalimumab, and other selected drug-cancer pairs are shown in S7 Table.

Importantly, this matrix assigns priority for subsequent rigorous pharmacoepidemiological validation; it mathematically does not convert GBD population burden into an exposure denominator for FAERS, nor does it establish definitive causal inference.

**Revised discussion (Conclusion):**

In conclusion, this study formalized a two-dimensional prioritization framework that successfully bridges spontaneous reporting signals with macro-epidemiological context. Evaluating FAERS and GBD in parallel highlights the critical distinction between reporting anomalies and true population-level burden. For instance, the 2021 ranitidine reporting excess in FAERS is best categorized as an administrative artifact compatible with stimulated reporting, rather than a sudden biological epidemic. Conversely, the rising US uterine-cancer trajectory identified via GBD represents a genuine macro-epidemiological shift, creating a setting in which any subsequently confirmed pharmacological risk factor would have magnified public-health consequences. Ultimately, to move from signal prioritization to causal validation, the hypotheses generated by our framework must transition to longitudinal electronic health record (EHR) or claims-based studies utilizing active comparators, incident users, indication adjustment, and prespecified latency windows [16-18].

## Comment 2

**Reviewer comment**

Second, the FAERS analysis raises important questions about the analytical unit and reproducibility. The manuscript reports 19,717 primary-suspect reports but also states that the contingency tables were constructed before final clinical-consistency exclusions, meaning that some target-event totals may represent drug-event record units rather than deduplicated independent reports. The authors should provide a stepwise accounting of raw records, duplicate removal, CASEID/PRIMARYID handling, male-record exclusion, clinical-consistency exclusions, and final independent cases for each cancer, and should clarify whether reports can contribute to more than one cancer category. Signal detection was restricted to the 50 most frequently reported drugs in each cancer group, which may introduce selection bias and does not establish that the remaining drugs were systematically screened. A complete drug-event screening flow or a strong prespecified rationale for the top-50 restriction is needed. A minimum of three reports plus meeting the threshold of any one of four algorithms was used to define a positive signal, yet many drug-cancer comparisons were performed without multiplicity control, sparse-data handling, threshold sensitivity analysis, or complete results against the database background. The authors should report the formulas, thresholds, confidence intervals, shrinkage parameters, zero-cell handling, and stability or multiple-testing analyses for each algorithm and should not equate positivity in any one algorithm with a robust signal.

### Response

### We deeply appreciate this highly astute methodological critique. Similar to the rigorous standards raised by Reviewer 1, your observations correctly identify areas where our statistical reporting and data-cleaning logic required greater transparency. We have systematically restructured our Methods and Results to address each of these valid concerns:

### 1. Stepwise Data Accounting and Analytical Units:

### We agree that the distinction between cohort-level independent patients and signal-level drug-event pairs was insufficiently explained. In the revised Section 2.2, we now clearly differentiate these units. The demographic descriptions (the 19,717 reports) utilize deduplicated patient records (unique PRIMARYID). However, the contingency tables for disproportionality were constructed using drug–event pair units immediately after primary-suspect restriction.

### To ensure full reproducibility, we have added a 6-step data deduplication sequence in the Methods and a corresponding workflow diagram (Fig. 1). Regarding your specific question on cross-category contribution: Yes, because the cancer categories are not mutually exclusive at the PRIMARYID level, a single deduplicated patient report containing multiple primary malignancy PTs can technically act as a case in more than one cancer-specific contingency table.

### 2. Prespecified Rationale for the Top-50 Restriction:

### Rather than a post-hoc selection bias, the top-50 restriction was a prespecified variance-reduction strategy, which we have now explicitly justified in Section 2.4 and Section 3.3. The FAERS database contains a massive “long tail” of highly fragmented, low-frequency reports. A pan-database systematic screen would introduce an unmanageable false-discovery rate (FDR) and severe sparse-data artifacts. By focusing on the top 50 drugs, we ensured the contingency tables were adequately powered. Importantly, our frequency inventory demonstrates that these top 50 drugs capture 72.8%–75.8% of the total primary-suspect reporting volume, effectively screening the high-frequency public health priorities while minimizing statistical noise.

### 3. Statistical Rigor, Multiplicity, and Thresholds:

### We completely agree that single-algorithm positivity is insufficient for a robust signal. Aligning with our revised F×G matrix (established in response to Reviewer 1), we now explicitly state that any-algorithm positivity is merely an exploratory screen, whereas our threshold for a principal signal (F3 tier) strictly requires four-algorithm concordance.

### Furthermore, Section 2.4 and the new Supplementary Materials have been expanded to detail the statistical parameters:

### Zero-cell handling: We specified the use of the Haldane-Anscombe half-step correction (adding 0.5 to cells) for frequentist calculations.

### Multiplicity & Shrinkage: We clarified that the Bayesian algorithms (IC and EBGM) incorporate inherent shrinkage parameters (empirical Bayes shrinkage towards the prior). This mathematical penalty against sparse data naturally mitigates false-discovery rates without requiring overly conservative Bonferroni adjustments. The top-50 restriction itself also inherently limited the multiple-testing burden to a predefined 150 candidate pairs (50 drugs × 3 cancers).

### These enhancements have significantly elevated the methodological rigor of the manuscript. The detailed changes in the text are provided below.

**Revised manuscript**

**Methods 2.1-2.4:**

**2.1 Study design and data sources**

This retrospective, observational pharmacovigilance study used the provided processed FAERS quarterly exports spanning the first quarter of 2004 through the fourth quarter of 2023. The exports link demographic (DEMO), drug (DRUG), reaction (REAC), outcome (OUTC), report-source (RPSR), indication (INDI), and therapy (THER) modules using PRIMARYID and CASEID. Female GBD 2023 estimates for China and the United States were downloaded from the IHME/GBD Results Tool on 15 June 2026. The FAERS component is therefore interpreted as an analysis of the provided reporting extracts, while GBD supplies independent population-level context. The FAERS case-selection and analytical workflow is summarized in Fig 1.

**2.2 Case definition and deduplication**

Cervical, ovarian, and uterine-cancer records were identified from explicit MedDRA PT vectors rather than treating all gynecological neoplasms as a single homogenous endpoint. Specifically, the cervical vector included CERVIX CARCINOMA, stage 0–IV terms, and CERVIX CARCINOMA RECURRENT. The ovarian vector included OVARIAN CANCER, stage I–IV, RECURRENT, METASTATIC, and specified advanced/biomarker-positive terms. The uterine vector included UTERINE CANCER, METASTATIC UTERINE CANCER, and ENDOMETRIAL CANCER, including stage 0–IV, RECURRENT, METASTATIC, and specified biomarker-positive terms. Therefore, “uterine cancer” serves as the overarching anatomical and analytical category throughout this study, while “endometrial cancer” is strictly mapped as a specific histological subset within the FAERS case definition. These terms are no longer treated as synonymous or mathematically interchangeable. The case-selection flow and cross-category PRIMARYID overlap are summarized in S1 and S2 Tables, and the MedDRA PT definitions, including recurrence, metastatic, and stage-specific terms, are listed in S3 Table.

To ensure strict reproducibility of the signal detection analysis, the data processing and deduplication workflow was executed in the following explicit sequence: (1) raw quarterly module import; (2) exact duplicate removal across all tables; (3) latest-case follow-up selection sorted by CASEID, FDA_DT, and PRIMARYID; (4) module linkage; (5) PT-based cancer classification; and (6) removal of male records or those with missing/implausible demographic data; and (7) role-based primary-suspect drug selection. Regarding Step 6, because FAERS is a spontaneous system susceptible to coding omissions, retaining records with “unknown” or “missing” sex for female-specific malignancies risks exposure misclassification. We adopted a strict complete-case approach, categorically excluding any record lacking explicit female demographic coding to ensure biological plausibility. The archived global flow file separately reports module-level deduplication totals prior to cancer-specific extraction.

It is necessary to distinguish between the analytical units used in different phases of this study. The processed cancer-specific DEMO exports contain one rigorously deduplicated row per patient (unique PRIMARYID), which were utilized to characterize cohort-level demographic patterns. In contrast, the contingency tables supplied for disproportionality screening were purposefully constructed using drug–event pair units. These pairs were generated immediately following the primary-suspect restriction. Regarding cross-category contribution, a single deduplicated patient report containing multiple distinct malignancy PTs can act as a case in more than one cancer-specific contingency table. This strategy ensures that the multi-algorithm matrix captures the specific reporting background without artificial truncation. Consequently, these signal tables should not be interpreted as independent cancer incidence counts, but rather as matrices for hypothesis prioritization.

**2.3 Drug normalization and report characterization**

Drug names were normalized with MedEx UIMA version 1.3.8, utilizing the generic, brand, alias, and combination-product mappings available in the project files. The resulting ingredient strings were mapped to ATC codes using the ATC search platform; the ATC level used for macro-level visualization was the first ATC character (anatomical main group). Reporter type and peak-year concentration for selected product groups are summarized in S4 Table. Recent pharmacovigilance work also recommends linking spontaneous-reporting signals to longitudinal healthcare data during signal management [16-18].

Primary-suspect (PS) status was strictly required for drug-level screening. Combination products were retained as their mapped ingredient strings when the mapping was available, whereas unresolved product-family strings were not treated as equivalent to a single prescription ingredient. These mapping rules and any remaining product-family ambiguity are explicitly reported so that the scope of the signal estimates remains transparent. Importantly, while restricting the analysis to primary-suspect records isolates the reporting behavior most directly attributed to the drug by the submitter, it does not emulate a controlled epidemiological design. This restriction does not eliminate confounding by indication, the effects of concomitant treatments, reverse causality, or the broader selection biases inherent to spontaneous pharmacovigilance databases.

**2.4 Disproportionality Algorithms and the Two-Dimensional Prioritization Matrix (F×G)**

To mitigate the statistical noise and inflated false-discovery rates characteristic of the low-frequency “long tail” in FAERS, our primary disproportionality analysis was restricted to a prespecified high-frequency candidate screen comprising the top 50 most frequently reported primary-suspect drugs for each malignancy. This threshold functions as a variance-reduction strategy, ensuring that subsequent multi-algorithm analyses operate on robust, adequately powered contingency tables while inherently limiting the multiple-testing burden to a predefined 150 candidate pairs (50 drugs × 3 cancers).

For each drug–event pair, a two-by-two contingency table was constructed. The cell definitions are presented in S5 Table, and the formulas and prespecified positivity thresholds for ROR, PRR, IC, and EBGM are summarized in S6 Table. Four disproportionality algorithms were employed: the Reporting Odds Ratio (ROR), Proportional Reporting Ratio (PRR), Empirical Bayes Geometric Mean (EBGM), and Information Component (IC). To address zero-cell handling in frequentist calculations (ROR and PRR), a Haldane-Anscombe half-step correction (adding 0.5 to all contingency cells) was applied. For the Bayesian algorithms (IC and EBGM), inherent shrinkage parameters toward the prior distribution mean mathematically penalize sparse data, naturally mitigating the false-discovery rate without requiring overly conservative Bonferroni adjustments.

The positivity thresholds were defined as follows: ROR lower 95% CI ≥1 with a≥3; PRR ≥2, χ2 ≥4, with a≥3; EBGM05 ≥2; and IC025 ≥0. Any-algorithm positivity was retained solely as an exploratory sensitivity screen and was not equated with a robust signal, whereas four-algorithm concordance was utilized as the principal signal set.

To systematically triage these signals, we explicitly defined a Two-Dimensional Prioritization Matrix (F×G) to determine the final validation priority.

The FAERS Dimension (F-score): This axis evaluates report-level evidence and algorithmic concordance. It is graded from F0 to F3: F0: No prespecified signal met; F1: Weak evidence (met only one algorithm or sparse evidence with <3 reports); F2: Moderate evidence (positivity in at least two algorithms with ≥3 reports); F3: Strong concordance (positivity across all four algorithms with an adequate case count).

Artifact Penalty: To mitigate stimulated reporting, a pre-specified artifact flag was introduced. If a signal exhibited a single-year concentration spike, a disproportionately high attorney-coded reporter ratio, or consumer-product ambiguity, its FAERS grade was downgraded by one level (e.g., F3 becomes F2).

The GBD Dimension (G-score): This independent axis evaluates the macro-level population burden trajectory using GBD estimates. It is graded from G0 to G2: G0: Stable or declining age-standardized incidence rate (ASR); G1: Uncertain, mixed, or fluctuating ASR trajectory; G2: Statistically significant increasing ASR trajectory.

The Decision Rule (Matrix Output): The final validation priority is assigned by cross-tabulating the F and G scores. The highest validation priority is assigned to F3–G2 combinations (strong FAERS concordance in a worsening epidemiological context). High priority is assigned to F2–G2, and moderate priority to F2–G0/G1. Low or uncertain priority is assigned to F0–F1 pairs. Signals triggering the artifact penalty are diverted to an “Artifact-focused review” tier, avoiding unnecessary clinical alarm. The resulting classifications for ranitidine, talc, tamoxifen, adalimumab, and other selected drug-cancer pairs are shown in S7 Table.

Importantly, this matrix assigns priority for subsequent rigorous pharmacoepidemiological validation; it mathematically does not convert GBD population burden into an exposure denominator for FAERS, nor does it establish definitive causal inference.

**Results 3.1-3.4:**

**3.1 Descriptive characteristics of adverse event reports**

Following the cancer-specific extraction and deduplication workflow, the FAERS exports contained 19,717 deduplicated patient records: 3,870 CC, 7,636 OC, and 8,211 UC. Because these cancer categories are not mutually exclusive at the PRIMARYID level (a patient may have multiple concurrent primary malignancies), their sum represents a category-specific record total rather than an absolute count of mutually exclusive individuals. The United States contributed the majority of reports, while China contributed only 11–27 reports per cancer category (≤0.3%).

**3.2 Drug classifications, temporal trends, and reporting patterns**

Primary-suspect drug reporting was concentrated in alimentary-tract/metabolism and antineoplastic/immunomodulating products. Annual reporting increased gradually through 2020 and showed a sharp 2021 peak, with ranitidine accounting for most peak-year records. Because attorney-coded reports constituted a substantial fraction of the ranitidine records during this period, this temporal spike is highly compatible with litigation-related stimulated reporting. However, it is important to note that FAERS does not formally encode litigation status, and attorney coding is not strictly equivalent to active litigation. Accordingly, reporter-type stratification serves as an indirect proxy rather than a definitive legal classification. The data cannot establish that litigation alone caused all or most of the excess reports, as concurrent media attention and notoriety effects likely contributed to the reporting surge.

**3.3 Frequently reported specific drugs and reporting profiles**

The top-50 lists for cervical, ovarian, and uterine cancer (Fig 3A-C, respectively) were used as a prespecified high-frequency candidate screen because the study objective was to identify interpretable, multi-algorithm signals rather than estimate exhaustive exposure risks for every recorded product. The top 50 drugs retained 72.8%–75.8% of all primary-suspect report-drug units. An additional 108–230 drugs with at least three primary-suspect reports remained outside the top 50, but these formed a fragmented, low-frequency tail susceptible to unstable estimates. Expanding the screen to 100 drugs increased coverage to 80.6%–83.3% (S8 Table). These estimates define a focused candidate screen and are not comparative exposure measures across all recorded products.

**3.4 Disproportionality analysis and signal generation**

The top-50 screen generated 63 exploratory positive drug-event pairs that met at least one algorithmic threshold; 42 met all four thresholds and are displayed in Fig 4. Four-algorithm concordance was observed for 14 cervical, 16 ovarian, and 12 uterine cancer pairs, while BH-FDR and Bonferroni retained 27–32 and 21–26 pairs, respectively (S9 Table). Ranitidine, talc, zinc oxide, and tamoxifen produced extreme reporting disproportionality, while adalimumab and other immunosuppressants remain hypotheses for pharmacoepidemiologic validation. These findings prioritize candidates for follow-up but do not establish a novel adverse drug reaction or a causal effect.

## Comment 3

**Reviewer comment**

Third, the case definition should be fully reproduced in the main manuscript rather than delegated solely to a supplementary table that is not visible in the submitted body. The authors should list each SMQ or PT, the site-specific rules, and the inclusion or exclusion of recurrence, metastasis, and terms related to prior malignancy, and should define the boundary between uterine and endometrial cancer. Excluding records coded as male does not resolve missing sex, coding error, or source-related differences, and sex should not be used only as a clinical-consistency filter without missingness and sensitivity analyses. Drug normalization also requires the MedEx version, brand-name, combination-product, and ingredient mapping rules, the ATC level used, and an assessment of the consequences of restricting analyses to primary-suspect drugs; this restriction does not remove confounding by indication, concomitant treatment, reverse causality, or selection bias.

### Response

We deeply appreciate this comprehensive methodological critique. Echoing the sharp insights provided by Reviewer 1 regarding our anatomical case definitions, your suggestions highlight essential areas where our data extraction and handling protocols required full transparency in the main text. We have addressed each of your points directly in the revised Methods:

**1. Case Definitions and PT Vectors (Section 2.2):**

We agree that the core case definitions must be immediately visible to the reader. The specific MedDRA Preferred Term (PT) vectors for cervical, ovarian, and uterine cancers—including the explicit inclusion of recurrence and metastasis terms—are now fully detailed in Section 2.2. Furthermore, we have clearly defined the boundary between uterine and endometrial cancer, establishing “uterine cancer” as the overarching analytical category, while treating “endometrial cancer” as a distinct histological subset, preventing inappropriate mathematical interchangeability.

**2. Sex, Missingness, and Clinical Consistency (Section 2.2):**

You correctly point out that simply excluding male records does not resolve underlying missingness or source-related coding errors. Because FAERS is a spontaneous reporting system, reliably imputing missing demographic data is mathematically fraught. Given the absolute biological specificity of gynecological malignancies, retaining records with “unknown” or “missing” sex introduces an unacceptable risk of exposure misclassification. Therefore, rather than conducting speculative sensitivity analyses on missing data, we opted for a strict, conservative complete-case approach: any record lacking explicit “female” coding was categorically excluded. We have clarified this rationale in Step 6 of the deduplication workflow.

**3. Drug Normalization and ATC Hierarchy (Section 2.3):**

Section 2.3 has been expanded to specify the use of MedEx UIMA version 1.3.8. We now detail our handling of generic/brand aliases, the retention of combination products as mapped ingredient strings, and the utilization of the first ATC character (anatomical main group) for our macro-level visualizations.

**4. Primary-Suspect Limitation and Confounding (Section 2.3):**

We completely agree with your epistemological caution. Restricting the analysis to primary-suspect (PS) drugs isolates the reporter’s immediate clinical suspicion, but it is not a substitute for controlled epidemiological design. We have added an explicit methodological disclaimer at the end of Section 2.3, directly stating that this restriction does not eliminate confounding by indication, concomitant treatment, reverse causality, or broader selection biases.

The detailed revisions to Sections 2.2 and 2.3 are provided below.

**Revised manuscript**

**Methods 2.2, 2.3:**

**2.2 Case definition and deduplication**

Cervical, ovarian, and uterine-cancer records were identified from explicit MedDRA PT vectors rather than treating all gynecological neoplasms as a single homogenous endpoint. Specifically, the cervical vector included CERVIX CARCINOMA, stage 0–IV terms, and CERVIX CARCINOMA RECURRENT. The ovarian vector included OVARIAN CANCER, stage I–IV, RECURRENT, METASTATIC, and specified advanced/biomarker-positive terms. The uterine vector included UTERINE CANCER, METASTATIC UTERINE CANCER, and ENDOMETRIAL CANCER, including stage 0–IV, RECURRENT, METASTATIC, and specified biomarker-positive terms. Therefore, “uterine cancer” serves as the overarching anatomical and analytical category throughout this study, while “endometrial cancer” is strictly mapped as a specific histological subset within the FAERS case definition. These terms are no longer treated as synonymous or mathematically interchangeable. The case-selection flow and cross-category PRIMARYID overlap are summarized in S1 and S2 Tables, and the MedDRA PT definitions, including recurrence, metastatic, and stage-specific terms, are listed in S3 Table.

To ensure strict reproducibility of the signal detection analysis, the data processing and deduplication workflow was executed in the following explicit sequence: (1) raw quarterly module import; (2) exact duplicate removal across all tables; (3) latest-case follow-up selection sorted by CASEID, FDA_DT, and PRIMARYID; (4) module linkage; (5) PT-based cancer classification; and (6) removal of male records or those with missing/implausible demographic data; and (7) role-based primary-suspect drug selection. Regarding Step 6, because FAERS is a spontaneous system susceptible to coding omissions, retaining records with “unknown” or “missing” sex for female-specific malignancies risks exposure misclassification. We adopted a strict complete-case approach, categorically excluding any record lacking explicit female demographic coding to ensure biological plausibility. The archived global flow file separately reports module-level deduplication totals prior to cancer-specific extraction.

It is necessary to distinguish between the analytical units used in different phases of this study. The processed cancer-specific DEMO exports contain one rigorously deduplicated row per patient (unique PRIMARYID), which were utilized to characterize cohort-level demographic patterns. In contrast, the contingency tables supplied for disproportionality screening were purposefully constructed using drug–event pair units. These pairs were generated immediately following the primary-suspect restriction. Regarding cross-category contribution, a single deduplicated patient report containing multiple distinct malignancy PTs can act as a case in more than one cancer-specific contingency table. This strategy ensures that the multi-algorithm matrix captures the specific reporting background without artificial truncation. Consequently, these signal tables should not be interpreted as independent cancer incidence counts, but rather as matrices for hypothesis prioritization.

**2.3 Drug normalization and report characterization**

Drug names were normalized with MedEx UIMA version 1.3.8, utilizing the generic, brand, alias, and combination-product mappings available in the project files. The resulting ingredient strings were mapped to ATC codes using the ATC search platform; the ATC level used for macro-level visualization was the first ATC character (anatomical main group). Reporter type and peak-year concentration for selected product groups are summarized in S4 Table. Recent pharmacovigilance work also recommends linking spontaneous-reporting signals to longitudinal healthcare data during signal management [16-18].

Primary-suspect (PS) status was strictly required for drug-level screening. Combination products were retained as their mapped ingredient strings when the mapping was available, whereas unresolved product-family strings were not treated as equivalent to a single prescription ingredient. These mapping rules and any remaining product-family ambiguity are explicitly reported so that the scope of the signal estimates remains transparent. Importantly, while restricting the analysis to primary-suspect records isolates the reporting behavior most directly attributed to the drug by the submitter, it does not emulate a controlled epidemiological design. This restriction does not eliminate confounding by indication, the effects of concomitant treatments, reverse causality, or the broader selection biases inherent to spontaneous pharmacovigilance databases.

## Comment 4

**Reviewer comment**

Fourth, the time-to-onset analysis does not support the statement that the observed distribution is "consistent with carcinogenesis." Treatment start and event dates in FAERS are frequently incomplete, and left truncation, diagnostic delay, reporting delay, unknown exposure duration, and pre-existing disease can strongly shape the distribution. A high proportion of intervals longer than 360 days and a Weibull shape parameter describe the reporting-time distribution, not cancer latency or a clinical hazard function. The authors should specify the date sources, missingness, exposure duration, censoring, repeat-report handling, model fitting, and comparison with alternative distributions, and should describe the result as a cautious temporal pattern in reports.

### Response

We are highly grateful for this rigorous statistical correction. You are entirely correct. Equating the FAERS time-to-onset (TTO) distribution with biological carcinogenesis was an epistemological overreach on our part. Similar to the cautions raised by Reviewer 1 regarding the intrinsic limitations of spontaneous reporting data, your insights rightly refocus this analysis on the structural realities of the database. We have entirely discarded the “carcinogenesis” terminology.

Following your precise guidance, we have fundamentally restructured Sections 2.5 and 3.5 to redefine TTO strictly as a descriptive, administrative reporting interval. Specifically, we have addressed your requirements as follows:

1. **Data sources & missingness**: The Methods now explicitly define the extraction from START_DT (THER module) and the event onset date, alongside the high missingness/invalidity rates (leaving valid fractions of only 13%–15%).
2. **Methodological parameters**: We clearly state the one-interval-per-PRIMARYID rule. Because reliable exposure end-dates are notoriously absent in FAERS, we explicitly declare that no exposure duration reconstruction or right-censoring mechanisms were applied.
3. **Model fitting & interpretation**: We clarify that the Weibull model was selected not to estimate a clinical hazard function, but solely as the standard descriptive parametric model for right-skewed pharmacovigilance data.
4. **Structural biases**: In the Results (Section 3.5), we have integrated your precise terminology, explaining that the observed patterns (including the β<1 shape parameter) are heavily shaped by left truncation, diagnostic/reporting delays, and the unmasking of pre-existing disease, rather than representing pharmacological latency.

The exact revisions detailing this cautious temporal pattern are provided below.

**Revised manuscript**

**2.5 Recorded time to onset**

Time-to-onset (TTO) was calculated from the recorded treatment start date (START_DT in the THER module) to the recorded adverse-event onset date in the processed DEMO files. Records with missing or non-numeric dates, non-positive intervals, or extreme intervals exceeding 3,600 days were excluded; strictly one valid interval per deduplicated PRIMARYID was retained. Reflecting the high degree of temporal missingness inherent to spontaneous reporting, the valid fractions of records meeting these criteria were 15.3% for cervical cancer, 14.8% for ovarian cancer, and 13.1% for uterine cancer.

Given the lack of reliable exposure end-dates in FAERS, no exposure-duration reconstruction or right-censoring mechanisms were utilized. The applied Weibull models serve as uncensored, purely descriptive fits to right-skewed observational data, rather than comparative survival analyses. Consequently, we explicitly define these metrics as administrative treatment-to-event reporting intervals. The medians, >360-day proportions, and distributional shapes characterize reporting patterns and diagnostic timelines; they do not demonstrate pharmacological latency, carcinogenic induction, or a true clinical hazard function. The Weibull shape parameter (β) is reported strictly as a mathematical descriptor of the data distribution.

**3.5 Time-to-onset distributions and parametric survival modeling**

The valid TTO subsets contained 591 cervical, 1,133 ovarian, and 1,072 uterine cancer records after stringent date and interval filtering (Fig 5A-C; Table 2). Intervals exceeding 360 days accounted for 55.16%, 58.25%, and 63.71% of these cohorts, respectively (S10 Table).

Rather than reflecting biological latency, these right-skewed distributions represent reported treatment-to-event intervals that are heavily shaped by structural factors in spontaneous reporting. As observed in the early incidence peaks, very short intervals frequently reflect left truncation, the unmasking of pre-existing undetected disease (protopathic bias), or immediate reporting following a delayed diagnosis. FAERS data lack the clinical granularity to distinguish these mechanisms from true acute events.

Subsequent Weibull modeling yielded shape parameters (β) below 1.0 across all cohorts (0.75–0.89). In the context of our analysis, a β<1 mathematically describes an interval distribution with the highest density of reports occurring early relative to a long temporal tail. This is not an epidemiological hazard estimate. We interpret these fitted parameters strictly as descriptive indicators of reporting behaviors—such as diagnostic delay and subsequent retrospective reporting—completely separate from biological mechanisms of oncogenesis.

Fig 5. Time-to-onset distributions for drug-associated gynaecological cancer reports.

Bar charts display reported treatment-to-event intervals for (A) cervical cancer (N=591), (B) ovarian cancer (N=1133), and (C) uterine cancer (N=1072). These intervals are administrative timing measures calculated from recorded treatment start to recorded event onset. They characterize spontaneous reporting behaviors and structural database biases; they do not represent biological cancer latency or a clinical hazard function.

## Comment 5

**Reviewer comment**

Fifth, the GBD analysis requires exact query conditions, GBD version and download date, female-population definition, cause hierarchy, measure definitions, standard population, units, uncertainty-interval method, and comparability checks between China and the United States. Because APC models require identification constraints, the authors should specify reference groups, smoothing or prior settings, overdispersion handling, diagnostics, and sensitivity analyses. Joinpoint analyses should report the candidate joinpoint range, selection criteria, and software settings, while the decomposition of population growth, aging, and epidemiological change should provide formulas and uncertainty propagation. The 2053 BAPC projections require retrospective validation or rolling forecast evaluation and visibly widening uncertainty intervals; long-term extrapolation should not be described as a deterministic or "predictable" public-health trajectory. More explicit tool-based methods for reproducible GBD extraction, code auditing, and result verification would strengthen this component (doi: 10.1002/mdr2.70069). The analysis of Joinpoint trends and structural inequities should also clarify variable selection, segment rules, age standardization, and the limits of interpretation (doi: 10.1002/mdb2.70025).

### Response

We sincerely thank you for setting out these exact reproducibility and methodological requirements for the population-level component. Similar to the sharp observations raised by Reviewer 1 regarding the structural limitations of secondary databases, your rigorous demands for explicitly defined parameters, identification constraints, and tool-based reproducibility fundamentally elevate the transparency of our GBD analysis. We deeply appreciate this level of methodological scrutiny.

Following your precise roadmap, we have surgically expanded Section 2.6 to provide full parameterization of the macro-epidemiological models, **ensuring compliance with the recommended reproducible workflows [Ref 10.1002/mdr2.70069; 10.1002/mdb2.70025]**.

Specifically, we have implemented the following refinements:

1. **GBD Query Constraints:** We detailed the exact cause hierarchies, standard population (WHO world standard), and database-provided 95% UI extraction mechanisms.
2. **Model Identifiability & Smoothing:** For the APC and BAPC models, we now explicitly state the reference groups (median age/period/cohort) and the use of a first-order random walk (RW1) prior to handle smoothing and overdispersion.
3. **Joinpoint & Decomposition:** We specified the Grid Search method and permutation tests for segment selection. We also clarified that the decomposition utilizes Das Gupta’s method, characterizing it strictly as arithmetic attribution rather than causal mediation, addressing the uncertainty propagation limits inherent to aggregate data.
4. **Forecast Interpretation:** As recommended, we implemented a rolling historical plausibility check. We explicitly frame the 2024–2053 trajectories as “exploratory scenarios with widening uncertainty,” entirely removing any deterministic or “predictable” terminology.

The detailed methodological additions, including the integration of your suggested frameworks, are documented in the revised manuscript below.

**Revised manuscript**

**Method 2.6:**

**2.6 GBD trend, age-period-cohort, decomposition and forecast analyses**

Female GBD 2023 estimates were queried for China and the United States (GBD cause hierarchies: B.4.3 Cervical cancer, B.4.4 Ovarian cancer, B.4.5 Uterine cancer) for four measures (Deaths, DALYs, Prevalence, and Incidence). Queries extracted both absolute Numbers and Rates per 100,000 for 1990–2023. Age-standardized rates (ASR) were computed using the WHO World Standard Population, with uncertainty intervals reflecting the GBD database-provided 95% UIs. The GBD 2023 source and disease-burden context are documented in the published GBD analysis [19]. To ensure robust comparability and code verification across structural disparities, our data extraction and processing strictly adhered to reproducible tool-based workflows **[20, Ref: 10.1002/mdr2.70069].**

Joinpoint input files were generated in rates per 100,000. The archived output utilized 0–5 candidate joinpoints, evaluating structural segments via the Grid Search method and a permutation test (4,500 permutations) applying familywise error rate control. A recent population-level Joinpoint analysis provides a methodological precedent for this segmented-trend approach **[21, Ref: 10.1002/mdb2.70025].** The China-specific burden estimates are also considered alongside published GBD analyses [22].

To resolve the non-identifiability problem inherent to Age-Period-Cohort (APC) models, we constrained the reference parameters to the median age group, period, and birth cohort. Analyses utilized five-year groupings. For the Bayesian age-period-cohort (BAPC) implementation, we integrated historical data (1990–2023) with UN 2024–2053 population projections. Model smoothing and overdispersion handling were achieved using a first-order random walk (RW1) prior for age, period, and cohort components (gf=5, secondDiff=TRUE). Recent age-period-cohort applications provide a comparable public-health trend-analysis context [23].

Changes in absolute disease burden were quantified using Das Gupta’s decomposition method, which we interpret strictly as the arithmetic attribution of population growth, ageing, and epidemiological rate changes, not as causal mediation or propagated probabilistic uncertainty. For long-term BAPC projections, we integrated a rolling historical plausibility check—comparing recent observed trend directions against initial forecast trajectories as an interpretive audit rather than independent validation. Forecasts for 2024–2053 remain purely exploratory scenarios characterized by visibly widening 95% UIs; they are not deterministic predictions. Related burden-projection work likewise treats long-horizon estimates as modelled scenarios rather than observed future outcomes [24].

Descriptive statistics summarized the FAERS exports. Continuous TTO variables are reported as medians and IQRs; categorical variables are reported as counts and percentages. Signal estimates are reported with 95% CIs where retained in the archived output. In addition to the prespecified algorithm thresholds, we calculated BH-FDR and 150-comparison Bonferroni sensitivity counts. The revised text distinguishes report frequency, disproportionality, signal prioritization, and causal validation. Statistical significance is not interpreted as evidence of causality.

...

**Results 3.6-3.10:**

**3.6 Spatiotemporal trends and Joinpoint analysis of gynaecological cancers (1990–2023)**

Subsequent Joinpoint temporal analysis revealed divergent population-level trends. The US uterine cancer category—which serves as the broader overarching GBD label, encompassing the substantial endometrial cancer subset identified in our FAERS analysis—showed increasing incidence (ASIR EAPC 1.67, 95% CI 1.57–1.77). Given the stark asymmetry in FAERS reporting volumes (with China accounting for ≤0.3% of reports), the GBD data for China and the United States were compared strictly to illustrate divergent macro-epidemiological trajectories. This analysis contrasts population-level disease burdens; it does not constitute a cross-national pharmacovigilance comparison. The GBD results provide baseline context for potential public-health consequences, not evidence that any reported drug caused these population-level shifts.

Joinpoint analysis showed distinct population-level trajectories. The United States generally showed smooth trends, including an increasing uterine cancer trajectory (ASDR APC approximately 2.8 in the highlighted segment), whereas China showed larger segment-to-segment changes, including declines in uterine cancer ASDR during 2010–2014 and increases in selected ovarian and uterine metrics after 2020. These differences are descriptive population patterns that may reflect demographic, diagnostic, and health-system factors; they are not attributed to a specific exposure in this study. The interpretation of these spatiotemporal segments is constrained to macro-level epidemiological shifts.

**3.7 Age-period-cohort (APC) analysis of disease trajectories**

The age-period-cohort analysis separated age, period, and cohort patterns for the three cancer groups. Cervical-cancer burden was concentrated in midlife in China, while several US incidence and prevalence measures peaked earlier and mortality remained higher at older ages. Period and cohort patterns generally moved in a favorable direction for cervical cancer in both settings. These patterns describe population heterogeneity and provide context for the design of future validation studies.

Ovarian-cancer profiles were dominated by later-life age effects, with older age peaks in the United States and a more midlife distribution in China. Period effects declined in the United States, whereas the Chinese series showed a flatter or modestly increasing pattern in the most recent years. The analysis is descriptive and does not identify the mechanisms underlying these differences.

Uterine-cancer patterns differed between the two countries. China showed midlife age peaks and declining period and cohort effects, whereas the United States showed increasing age-specific risk at older ages and stronger positive period effects after 2009. The cohort pattern is compatible with changing population risk across generations, but these aggregate data do not identify a single cause or permit attribution to obesity or another individual-level exposure. The extracted reference parameters serve strictly to baseline these descriptive models.

**3.8 Age-specific demographic and epidemiological landscape in 2023**

To describe the contemporary population distribution of disease burden, we evaluated age-specific estimates for the three gynaecological cancers in 2023 (Fig. 8), separating absolute case numbers from rates per 100,000 population.

In the United States (Fig. 8A–D), absolute case numbers varied with the underlying population structure, whereas rates per 100,000 were highest at older ages for uterine and ovarian cancer. Cervical-cancer rates were concentrated earlier in adulthood, with mortality remaining elevated at older ages. These are population-level rate patterns rather than individual exposure risks.

In China (Fig. 8E–H), cervical-cancer rates were concentrated in midlife, while ovarian and uterine rates were lower across most age groups and generally declined at older ages. The contrast with the United States is a population-level difference in age-specific burden and should not be interpreted as evidence for a particular drug or mechanism.

Together, the 2023 age-specific estimates provide ecological context for interpreting FAERS signals. They identify where a validated drug-associated signal could have greater population consequence, while preserving the distinction between background burden and medicine-attributable risk.

**3.9 Decomposition of absolute burden changes (1990–2023)**

We decomposed changes in absolute burden between 1990 and 2023 into population growth, population ageing, and epidemiological-rate change (Fig. 9). Population growth contributed positively in both countries, while the net direction depended on the balance between demographic components and rate change.

In China, negative contributions from epidemiological-rate change offset the upward demographic components for most outcomes. Uterine-cancer prevalence was the main exception in the displayed estimates, with a small net increase associated with demographic change.

In the United States, cervical and ovarian outcomes showed combinations of demographic growth and favorable rate changes, whereas uterine cancer showed positive contributions from both population growth and epidemiological-rate change. Following Das Gupta’s framework, the decomposition describes arithmetic contributions to burden change; it does not establish the causes of the underlying rate trends nor propagate individual-level uncertainty.

The decomposition results provide independent population-level context for prioritization: they quantify the arithmetic contributions of population growth, ageing, and epidemiological-rate change to absolute burden differences, without attributing those changes to FAERS exposures.

**3.10 Long-term forecasting of disease burden (2024–2053)**

To forecast future trajectories, we applied Bayesian age–period–cohort models using the stated population projections, age weights, priors, and model structure. These projections are exploratory scenario estimates. A rolling historical plausibility check was added to compare the direction of recent observed trends with the forecast direction; the check supports interpretation of the model output but is not an independent validation study…

In China, projected age-standardized rates may decline while absolute burden remains substantial …

The United States presents a high projected uterine-cancer burden, but the apparent smoothness of a modelled curve should not be interpreted as certainty. Long-horizon extrapolation is sensitive to population projections, …

## Comment 6

**Reviewer comment**

Sixth, the Results and Discussion should more rigorously separate signal detection, signal prioritization, and causal validation. The 2021 ranitidine peak is a useful example of stimulated and legally influenced reporting, but "lawyer-coded" records are not automatically equivalent to litigation reports, and FAERS does not directly record litigation status; the basis for this classification and sensitivity analyses using alternative definitions should be provided. Talc and zinc oxide also involve consumer products, product-family misclassification, and inconsistent exposure definitions and should not be placed on the same causal interpretive level as prescription medicines. Findings for adalimumab and other immunosuppressants should be presented as hypotheses requiring validation, with stratification by indication, combination therapy, prior cancer, reporter type, and calendar time rather than being called "novel signals" without an external validation pathway. The very small proportion of Chinese reports in FAERS cannot be compared directly with Chinese GBD burden to infer national drug risk; the manuscript should make clear that it juxtaposes FAERS system footprint with population burden, not Chinese and US exposure or incidence risks.

### Response

We deeply appreciate this comprehensive synthesis. Similar to the sharp observations raised by Reviewer 1 regarding the structural limitations of secondary databases, your critique fundamentally elevates the manuscript by enforcing a rigorous conceptual boundary among signal detection, prioritization, and causal validation. We completely agree that administrative reporting outputs must not be conflated with validated epidemiologic risks.

Following your precise recommendations, we have surgically refined the Results and Discussion to recalibrate the evidentiary weight of each finding:

- Ranitidine & Litigation: We explicitly clarified that while the 2021 peak is highly compatible with stimulated reporting, FAERS does not formally encode active litigation, and “lawyer-coded” status serves as a proxy requiring careful interpretation rather than an absolute legal definition.
- Talc & Zinc Oxide: We now explicitly distinguish these consumer products from prescription medicines, acknowledging the inherent vulnerabilities to product-family misclassification and inconsistent exposure definitions.
- Immunosuppressants (e.g., Adalimumab): We entirely removed any implication of these being “novel signals” standing on their own. Instead, they are strictly framed as hypotheses requiring external validation pathways, specifically listing your recommended stratifications (indication, combination therapy, prior cancer, reporter type, and calendar time).
- China-US GBD Juxtaposition: We clarified the conceptual framework to explicitly state that the analysis juxtaposes the FAERS system footprint with macro-population burden, strictly avoiding any inference of national drug-exposure incidence or comparative epidemiological risk between the two countries.

We believe these targeted revisions, detailed below, finalize a highly transparent and methodologically defensible manuscript.

**Revised manuscript**

**Methods 3.2-3.4:**

**3.2 Drug classifications, temporal trends, and reporting patterns**

Primary-suspect drug reporting was concentrated in alimentary-tract/metabolism and antineoplastic/immunomodulating products. Annual reporting increased gradually through 2020 and showed a sharp 2021 peak, with ranitidine accounting for most peak-year records. Because attorney-coded reports constituted a substantial fraction of the ranitidine records during this period, this temporal spike is highly compatible with litigation-related stimulated reporting. However, it is important to note that FAERS does not formally encode litigation status, and attorney coding is not strictly equivalent to active litigation. Accordingly, reporter-type stratification serves as an indirect proxy rather than a definitive legal classification. The data cannot establish that litigation alone caused all or most of the excess reports, as concurrent media attention and notoriety effects likely contributed to the reporting surge.

**3.3 Frequently reported specific drugs and reporting profiles**

The top-50 lists for cervical, ovarian, and uterine cancer (Fig 3A-C, respectively) were used as a prespecified high-frequency candidate screen because the study objective was to identify interpretable, multi-algorithm signals rather than estimate exhaustive exposure risks for every recorded product. The top 50 drugs retained 72.8%–75.8% of all primary-suspect report-drug units. An additional 108–230 drugs with at least three primary-suspect reports remained outside the top 50, but these formed a fragmented, low-frequency tail susceptible to unstable estimates. Expanding the screen to 100 drugs increased coverage to 80.6%–83.3% (S8 Table). These estimates define a focused candidate screen and are not comparative exposure measures across all recorded products.

**3.4 Disproportionality analysis and signal generation**

The top-50 screen generated 63 exploratory positive drug-event pairs that met at least one algorithmic threshold; 42 met all four thresholds and are displayed in Fig 4. Four-algorithm concordance was observed for 14 cervical, 16 ovarian, and 12 uterine cancer pairs, while BH-FDR and Bonferroni retained 27–32 and 21–26 pairs, respectively (S9 Table). Ranitidine, talc, zinc oxide, and tamoxifen produced extreme reporting disproportionality, while adalimumab and other immunosuppressants remain hypotheses for pharmacoepidemiologic validation. These findings prioritize candidates for follow-up but do not establish a novel adverse drug reaction or a causal effect.

**Discussion (Relevant part):**

Ranitidine illustrates why toxicological plausibility and reporting magnitude must be separated. The external multinational cohort study did not reproduce the same cancer association in its design, providing contextual evidence rather than direct validation or refutation of the FAERS signal. Differences in formulation, exposure ascertainment, follow-up timelines, and outcome definitions prevent a one-to-one comparison. The appropriate conclusion is that the FAERS disproportionality is hypothesis-generating and requires exposure-denominator studies. The FAERS-based ranitidine analysis, the multinational cohort study, and the randomized NDMA pharmacology trial provide complementary but non-equivalent evidence [25-27].

Tamoxifen provides a robust positive-control context because its specific endometrial pharmacology—acting as a partial estrogen agonist in the endometrium—is biologically plausible and well-supported by external clinical evidence. Concurrently, our analysis of the broader FAERS uterine cancer records found that most tamoxifen-associated reports also contained a breast cancer or anti-estrogen indication. This compatibility with known histological mechanisms does not, however, validate the FAERS effect size, which remains vulnerable to confounding by indication, surveillance bias, menopausal status, and survivorship [28].

Talc and zinc oxide illustrate product and reporting-channel ambiguity. In the audit, the OC talc group included 147 unique primary-suspect reports, with 59.9% consumer-coded and 14.3% lawyer-coded records; the zinc-oxide group included 152 reports and was predominantly consumer-coded. These consumer products involve inherent product-family misclassification and inconsistent exposure definitions; therefore, they should not be placed on the same causal interpretive level as prescription medicines. The extreme RORs are best treated as reporting-distortion hypotheses requiring independent exposure assessment. The epidemiological literature on talc and cancer remains sensitive to exposure definition and study design [29,30].

Findings for adalimumab and other immunosuppressants are presented exclusively as hypotheses requiring an external validation pathway. Given the complex clinical background of these patients, future validation studies must rigorously stratify by indication, combination therapy, prior cancer, reporter type, and calendar time to isolate potential pharmacological risks from underlying disease progression.

The FAERS–GBD juxtaposition contributes to prioritization in two ways. First, the sharp 2021 FAERS peak is not mirrored by a comparable abrupt change in the macro-level cancer series, which argues against interpreting the peak as a simple population-incidence surge and supports review of reporting dynamics, particularly late-stage stimulated reporting distinct from classical post-marketing trends. Second, the increasing US uterine-cancer trajectory indicates a setting in which a subsequently validated risk factor could have greater public-health consequence. The GBD layer changes the consequence of validation, not the causal credibility of the initial FAERS association. The broader gynecological-cancer burden literature documents substantial geographic heterogeneity and forward-looking burden differences [31,32].

China was included as a prespecified population comparator rather than as a denominator for the predominantly US FAERS reports. The small number of Chinese FAERS reports is reported directly and is not used to infer lower or higher national drug risk. This framework explicitly juxtaposes the FAERS system footprint against macro-population burden, rather than comparing Chinese and US exposure or incidence risks. The contrasting GBD trajectories illustrate why a candidate signal may warrant different follow-up priority in different health-system and demographic settings. Recent China-specific GBD analyses provide relevant population-level context without supplying an exposure denominator for FAERS [22].

The age-period-cohort (APC) and decomposition analyses further contextualize this population framework. The concentration of ovarian and uterine burdens in later life dictates that downstream validation studies must rigorously account for cumulative environmental exposures, polypharmacy, competing mortality risks, and age-dependent diagnostic intensity. Demographic transitions—specifically population growth and aging—can drive absolute case numbers upward even when age-standardized rates decline. For uterine cancer in the United States, the positive contributions of epidemiological and population-growth components, coupled with escalating period effects, demonstrate that demographic shifts alone are insufficient to account for the overall increase [9,23]. While such forecasts are essential for health service planning, their expanding confidence intervals and inherent sensitivity to model specifications render them the most uncertain analytical layer. Consequently, they should inform scenario planning rather than dictate definitive pharmacovigilance conclusions [24].

Methodological strengths include the 20-year observation window, explicit documentation of the deduplication workflow, explicit PT definitions, restriction of drug-level screening to primary-suspect records, four complementary signal algorithms, multiplicity sensitivity counts, reporter/product audits, and preservation of the processed source outputs. The revised framework follows the central READUS-PV principle that disproportionality findings should be reported with their analytical unit, thresholds, uncertainty, and external-context limitations [11].

Several methodological constraints remain. FAERS lacks reliable exposure denominators and comprehensive longitudinal histories; the prespecified high-frequency top-50 design creates a deliberate selection boundary, which inherently limits our ability to detect rare signals associated with low-frequency or newly marketed medications; the signal tables use record-level drug–event units generated before the final clinical-consistency review; and residual confounding, product-family misclassification, stimulated reporting, and ecological fallacy remain possible. GBD estimates describe population trajectories rather than drug-exposed cohorts. TTO values are administratively reported intervals, and BAPC projections are scenario estimates. These boundaries define the appropriate next step—validation in longitudinal EHR or claims data—while preserving the value of the FAERS–GBD framework for structured hypothesis prioritization. Recent pharmacovigilance position statements and EHR signal-identification reviews support this staged validation pathway [16-18].

Evaluating FAERS and GBD in parallel highlights the critical distinction between reporting anomalies and true population-level burden. For instance, the 2021 ranitidine reporting excess in FAERS is best categorized as an administrative artifact compatible with stimulated reporting, rather than a sudden biological epidemic. Conversely, the rising US uterine-cancer trajectory identified via GBD represents a genuine macro-epidemiological shift, creating a setting in which any subsequently confirmed pharmacological risk factor would have magnified public-health consequences. To move from signal prioritization to causal validation, the hypotheses generated by our framework must transition to longitudinal electronic health record (EHR) or claims-based studies utilizing active comparators, incident users, indication adjustment, and prespecified latency windows [16-18].

# Concluding Remarks:

In conclusion, we would like to reiterate our most sincere appreciation to both Reviewer #1 and Reviewer #2 for their dedication, exceptional expertise, and immense patience in guiding the revision of this manuscript. Navigating the complexities of combining FAERS and GBD databases requires the exact type of rigorous methodological scrutiny and epistemological caution that you both provided.

Through the extensive revisions—spanning the formalization of the prioritization framework, the step-by-step transparency in data deduplication, the precise MedDRA PT vector definitions, and the strictly recalibrated language regarding causal inference—we are confident that the manuscript is now vastly stronger, scientifically rigorous, and fully transparent. **We hope that this comprehensively upgraded version now meets your high standards and the rigorous requirements of the journal.** We remain entirely open to any further suggestions you might have.

Thank you once again for your invaluable contribution to our work.

All co-authors
